# Fishing out AIEC with FimH capturing microgels for inflammatory bowel disease treatment

**DOI:** 10.1038/s41467-025-63276-7

**Published:** 2025-08-25

**Authors:** Jialin Wu, Yutao Liu, Ruiying Liu, Changyi Xiao, Leyan Xuan, Lili Wu, Jiamin Qian, Xudong Qin, Yingying Hou, Maobin Xie, Xiyong Yu, Bin Liu, Guosheng Tang

**Affiliations:** 1 Guangzhou Municipal and Guangdong Provincial Key Laboratory of Molecular Target & Clinical Pharmacology, the NMPA and State Key Laboratory of Respiratory Disease, School of Pharmaceutical Sciences and the Fifth Affiliated Hospital, Guangzhou Medical University, Guangzhou, Guangdong, P. R. China; 2 School of Life Sciences, Faculty of Medicine, Tianjin University, Tianjin, China; 3 National Key Laboratory of Intelligent Tracking and Forecasting for Infectious Diseases, TEDA Institute of Biological Sciences and Biotechnology, Nankai University, Tianjin, P. R. China; 4 The Fourth Affiliated Hospital of Guangzhou Medical University; School of Biomedical Engineering, Guangzhou Medical University, Guangzhou, P. R. China; 5 Key Laboratory of Molecular Microbiology and Technology, Nankai University, Ministry of Education, Tianjin, P. R. China; 6 Nankai International Advanced Research Institute, Shenzhen, P. R. China

**Keywords:** Drug delivery, Ulcerative colitis, Bacterial pathogenesis

## Abstract

Inflammatory bowel disease (IBD) is a chronic immune-mediated condition with rising global incidence and limited treatment options. Current therapies often have poor efficacy and undesirable side effects. Here we present a drug-free strategy that targets bacterial adhesion to manage IBD. We develop porous microgels loaded with mannan oligosaccharides (MOS) that mimic the natural binding sites of intestinal cells. These microgels attract adherent-invasive Escherichia coli (AIEC) by interacting with FimH, a bacterial protein used for attachment, thereby preventing AIEC from colonizing the gut lining. The microgels are fabricated using an all-aqueous two-phase system, enabling biocompatibility and structural control. In a mouse model of IBD, this competitive adsorption approach alleviates intestinal inflammation, reduces harmful Enterobacteriaceae, and enhances gut microbial diversity. This work introduces a non-antibiotic, bioinspired method that intercepts pathogenic bacteria and restores microbial balance, offering a promising therapeutic strategy for IBD.

## Abstract (web summary)

Inflammatory bowel disease (IBD) is a chronic immune-mediated condition. This study presents a drug-free treatment strategy for IBD that targets bacterial adhesion, utilizing mannan oligosaccharide-loaded porous microgels to selectively capture adherent-invasive E. coli and competitively inhibit bacterial colonization on the gut epithelium.

## Introduction

Inflammatory bowel disease (IBD) is an immune-mediated intestinal disorder with complex pathophysiological mechanisms involving genetic, environmental, microbial, and immunological factors^1,2^. The rapidly increasing worldwide prevalence of IBD, driven by rising incidence and improved survival, necessitates urgent implementation of preventive measures and therapeutic advancements to mitigate its expanding public health impact^3^. The primary types of IBD include ulcerative colitis (UC), Crohn’s disease (CD), and indeterminate colitis (IC). Unlike common intestinal inflammation, which is typically acute and curable with anti-infective treatment, IBD is characterized by a prolonged course with recurrent episodes and remains incurable to date^4,5^. The clinical manifestations of IBD are varied, encompassing gastrointestinal symptoms such as diarrhea, abdominal pain, bloody stools, and perianal abscesses^6^. The etiology of IBD is multifactorial, including psychological stress, autonomic nervous system dysfunction, dysbiosis of gut microbiota, and immune regulation associated with disease activity^7^. The primary characteristics of gut microbiota imbalance are an increase in mucosa-associated bacteria and a decrease in overall biodiversity^8^. Although no single pathogenic microorganism has been definitively identified, many studies have reported that alterations in pathogens and commensal bacteria may be involved in the disease’s pathogenesis^9^.

Among these bacteria, adherent-invasive *Escherichia coli* (AIEC) is increasingly recognized as a major player in the pathogenesis of CD^10–12^. AIEC plays a crucial role in the pathogenesis of CD through its ability to adhere to and invade intestinal cells, survive within macrophages, induce pro-inflammatory responses, disrupt the intestinal barrier, and contribute to gut dysbiosis^13^. Upon entering the intestine, AIEC strains adhere and invade epithelial cells via type I pili adhesin FimH, which has important clinical significance in the pathogenesis of CD^14^. Specifically, FimH directly binds to mannose residues on carcinoembryonic antigen-related cell adhesion molecule 6 (CEACAM6) glycoproteins present on the surface of intestinal epithelial cells, which facilitates the initial attachment of AIEC to the host cells^15,16^.

The treatment of IBD still faces significant challenges, as most current targeted strategies exhibit limited therapeutic efficacy and are often associated with adverse effects and the emergence of drug resistance^17^. Recent studies have shown that disrupting the crucial interactions among CEACAM6, FimH, and mannose can effectively reduce the pathogenicity of AIEC, offering a promising approach for the management of CD^18^. However, these therapeutics often suffer from poor target selectivity, limited bioavailability, or the need for repeated administration, and their stability and safety in the complex intestinal environment remain insufficient^19^.

Microgels, micron-scale granular hydrogels, have gained significant attention due to their small size, high flexibility, good injectability, porosity, and large specific surface area. Such unique characteristics make them a promising option for use as carriers in drug delivery and cell therapy. However, traditional microfluidic fabrication often involves oils and surfactants, limiting biocompatibility. The ATPS-based strategy offers an oil-free, surfactant-free alternative, enabling the biofabrication of microgels for applications in tissue engineering, drug screening, and disease modeling.

**Fig. 1:**
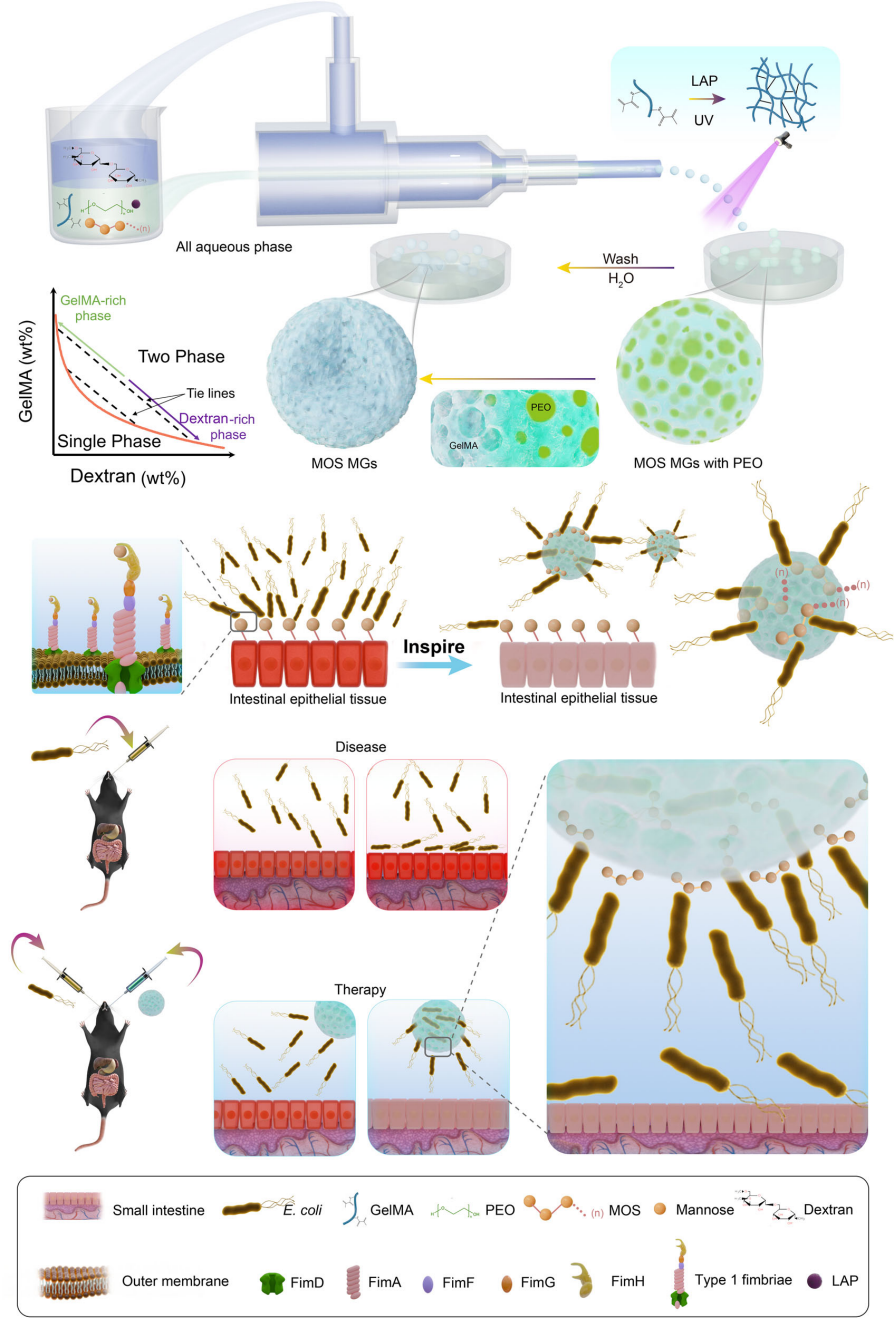
Schematic overview of porous MOS microgels for IBD treatment. Schematic illustration of porous MOS MGs fabrication by merging all-aqueous and co-axial microfluidic technologies and prompting microgels competitively absorbing AIEC over intestinal epithelial cells for the treatment of IBD by leveraging the binding properties of FimH with mannose.

Here, as schematically briefed in Fig. 1, we present a robust method for fabricating porous microgels loaded with oligosaccharides (MOS, an oligomer of mannose) by merging all-aqueous and coaxial microfluidic technologies. Instead of directly delivering drugs, which may lead to toxicity or resistance, we propose a competitive adsorption strategy, where microgels, by leveraging the specific binding affinity of FimH to mannose, selectively capture AIEC and reduce its colonization of intestinal epithelial cells. By lowering AIEC abundance while sparing beneficial microbes, this approach helps restore gut microbiota balance and potentially alleviates IBD.

## Results

### Fabrication and characterization of MOS MGs

The MOS MGs (MOS-loaded porous GelMA microgels) were fabricated through the ATPS-based strategy. To achieve stable stratification with GelMA, after experimenting with various biomaterials, we selected dextran, which can form stable interfaces with GelMA (Supplementary Fig. 1A). To further fabricate porous microspherical microgels, GelMA and Polyethylene oxide (PEO) were mixed as dispersed phases in the fabrication systems. We have demonstrated previously that GelMA and PEO aqueous solutions can be utilized to induce the formation of an ATPS-based strategy allowing facile bioprinting of micropore-forming hydrogels^20,21^. Accordingly, we hypothesized that this same system, with further optimizations, may be able to enable the direct fabrication of porous microspherical microgels.

**Fig. 2:**
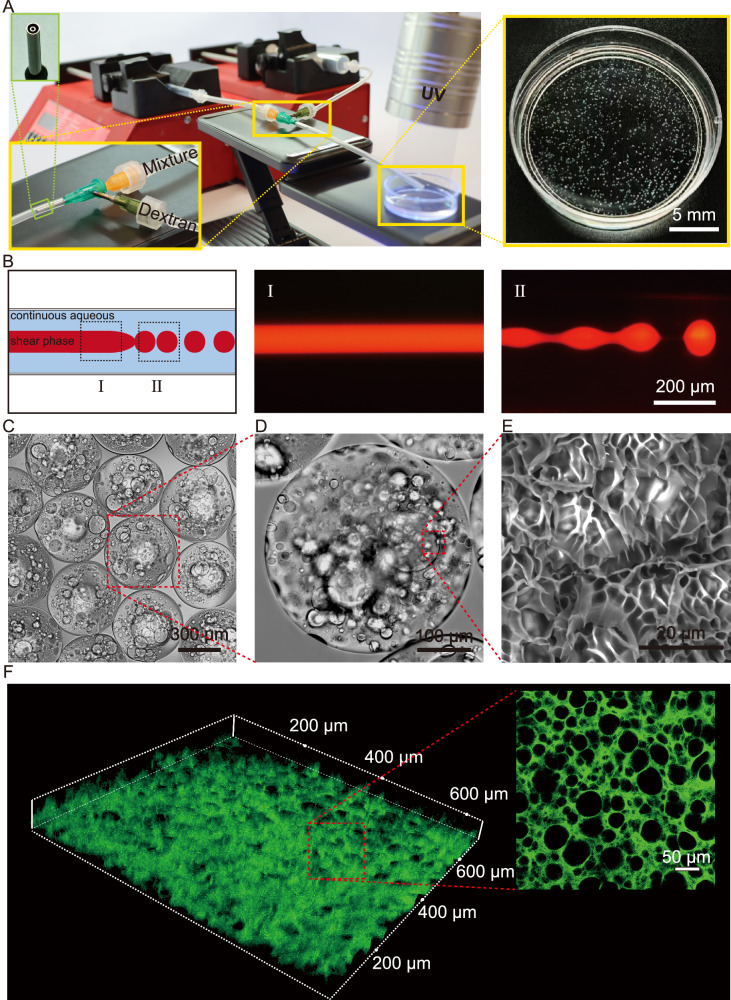
The fabrication of porous MOS MGs by all-aqueous co-axial microfluidics. **A** Images of the equipment to generate porous microgels. **B** Schematic illustration and corresponding fluorescence microscopy images of the fabrication procedure of the fibers (I) and microspheres (II). **C**, **D** Bright-field microscopy images of MOS MGs. (*n *= 3 biological replicates). **E** SEM image of MOS MGs. (*n *= 3 biological replicates).** F** Confocal *Z*-axis scanning images of MOS MGs cross-sections. Source data are provided as a Source Data file.

As shown in double-phase diagrams, within the concentration ranges employed in this study, they are capable of forming stable phase-separated systems (Supplementary Fig. 1A, B). The setup for biofabrication consisted of an injection digital pump, a custom-designed coaxial needle system with an extended soft channel, and a collecting bath (Fig. 2A). As shown in Fig. 2B, in this process, utilizing dextran to shear a mixture of GelMA phase (involved GelMA, MOS, and Lithium phenyl-2,4,6-trimethylbenzoylphosphinate (LAP)) and PEO solution into spherical microdroplets, followed forming microspheres by UV curing. Finally, remove uncured PEO via water washing, resulting in porous MOS GelMA microgels. As shown in Fig. 2C–E, microspherical microgels were attainable. Interestingly, in addition to microspherical microgels, microfibers can be fabricated by simply selecting the specific curing positions in the channel without changing any parameters or setups (Fig. 2B**and** Supplementary Fig. 2). Microgel carriers should allow efficient bacterial adsorption. To illustrate such a key structure, we further investigate the penetration of pore structure in microgels. As shown in Fig. 2F, fluorescence images indicated the interconnected pore structures inside microgels. Subsequently, we conducted a characterization of the microsphere dimensions and porosity. Microgels with different sizes could be prepared simply by increasing the flow rate of the continuous phase. In addition, all the resultant microgels maintain a very low polydispersity (Supplementary Fig. 3A–H). Moreover, by altering the volume ratio between the porogen (PEO) and GelMA (e.g., changing the GelMA-to-PEO volume ratio from 10:1 to 5:1 and 2:1), we successfully achieved tunable control over the porosity of the microgels (Supplementary Fig. 3I, J). Compared to conventional microgels, porous microgels have a larger specific surface area for bacterial adsorption and exhibit a longer residence time in the body. To interpret the benefit of the porous microgels, porous and solid microgels are all fabricated using this strategy (Supplementary Fig. 4A). We further compared the adsorption effects of solid microgels and porous microgel on wild type AIEC strain LF82 (WT) (Supplementary Fig. 4B), compared with solid microgels, the porous microgel group shows ultra-high bacterial adsorption ability. We also used In Vivo Imaging Systems (IVIS) to compare the in vivo retention times of different microgels in mice. Similar to our expectations, porous microgels indeed have a longer residence time in the body (Supplementary Fig. 4C).

### Mechanical mechanics of MOS MGs

The effect of the micro-porous structure on the mechanical properties of the hydrogel constructs was also investigated. We evaluated the self-recovery behaviors of the MOS MGs with different initiation systems using a compression cycle test. First, we tested the mechanical properties of MOS MGs. The Young’s modulus of the microgel is 22.7 ± 0.7 kPa, and it has good compressive resilience (Supplementary Fig. 5A). The elastic recovery rate, the cohesiveness ratio, and the resilience of the microsphere are 96.9% ± 0.9%, 85.4% ± 4.8%, and 84.9% ± 2.5%, and it has good recovery ability (Supplementary Fig. 5B–D). The prepared hydrogels can reach over 50% strain deformation and immediately recover their original shape after the removal of compression force (Supplementary Fig. 5E–G), showing good flexibility. Furthermore, to assess whether the adsorption of AIEC affected the mechanical integrity of the microgels, we analyzed the stress–strain curves of microgels after co-incubation with AIEC. No significant difference was observed compared to the control group without AIEC (Supplementary Fig. 5H), suggesting that the adsorption process did not compromise microgel mechanics. To determine whether the AIEC-bound microgels could be excreted from the body, we collected fecal samples from treated mice and successfully isolated the microgels (Supplementary Fig. 5I). This shows that MOS MGs can maintain their spherical form during intragastric administration, which is an ideal carrier for drug delivery.

### Biocompatibility studies of MOS MGs

**Fig. 3:**
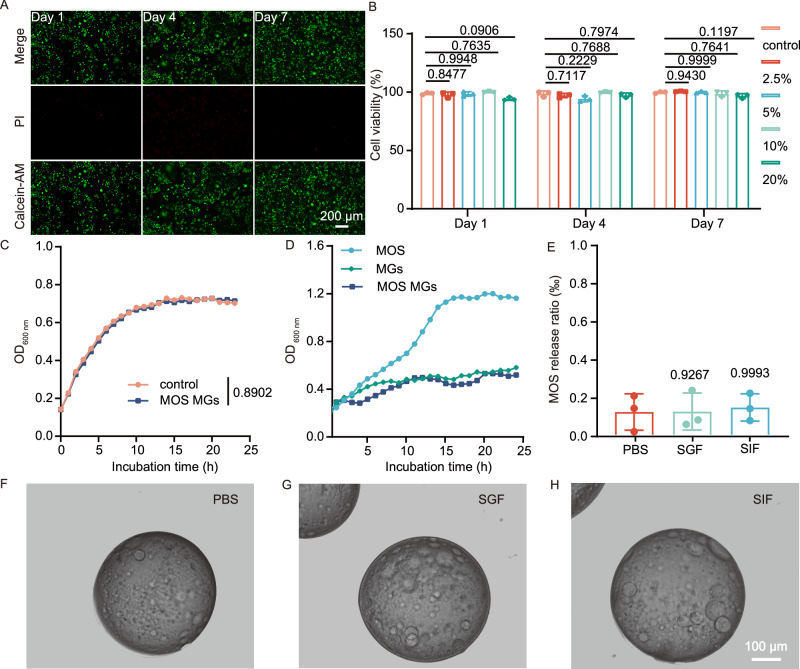
Cellular behaviors and stability of MOS MGs. **A**,** B** Biocompatibility assessment of MOS MGs using Caco-2 cells: Live/Dead cell staining (**A**) and CCK-8 assay (*n* = 3) (**B**).** C** Growth curve of AIEC in LB medium with or without the addition of MOS MGs (*n* = 3). **D** Growth curves of AIEC under conditions where MOS, MGs, and MOS MGs serve as the only carbon sources (*n* = 3). **E** UV-Vis spectroscopy analysis of MOS release into the solution (*n* = 3 biological replicates). **F**–**H** Bright-field microscopy images of MOS MGs immersed for 48 h in PBS, SGF, and SIF. Significance was determined by one-way ANOVA and indicated as the *P*-value (**B**, **E**). * *P* < 0.05, ** *P* < 0.01, *** *P* < 0.001, **** *P* < 0.0001; n.s. no significant difference. Data are presented as mean ± s.d. (**B**, **E**). Source data are provided as a Source Data file.

Subsequently, we evaluated the biocompatibility of MOS MGs both in vitro and in vivo. Initially, an in vitro biocompatibility assessment was performed. Cell viabilities were characterized using Live/Dead staining in Caco-2 and HeLa cells, with green fluorescence indicating live cells and red fluorescence indicating dead cells. The cells were cultivated for up to 7 days. It was found that the cell viability remained as high as 90% during the entire course of culture, and the group of MOS MGs (Fig. 3A and Supplementary Fig. 6A) showed no significant changes with other groups in cell viability over time. Colorimetric assays were conducted on varying concentrations of these MGs in the Caco-2 and HeLa cell lines. The results indicated no significant cellular toxicity across concentrations ranging from 0 to 20% for MOS MGs (Fig. 3B and Supplementary Fig. 6B).

To further emphasize the versatility and superiority of our proposed method, we compared cell culture performance using different types of microgels: conventional homogeneous microgels prepared by traditional oil-phase microfluidics and microgels prepared by the ATPS-based strategy. As shown in Supplementary Fig. 6C, D, the microgels fabricated via our ATPS-based strategy exhibited significantly higher cell viability on days 1, 4, and 7 compared to those produced by conventional oil-shearing microfluidics. In addition, the expression of functional proteins was markedly enhanced. Furthermore, when porous microgels generated via an ATPS-based strategy were used as carriers, both cell viability and functional protein expression were further improved. These results clearly demonstrate that, compared to conventional approaches, our method offers substantial advantages in terms of cytocompatibility and cellular functionality.

Next, to evaluate the systemic biosafety of the MOS MGs in vivo, the major organs of heart, liver, spleen, lung, and kidney were harvested for hematoxylin and eosin (H&E) slices were carried by us. Compared with the PBS group, the H&E staining of the main organs treated with MOS MGs had no noticeable tissue damage and changes in morphology, indicating that MOS MGs had no obvious biological toxicity (Supplementary Fig. 6E).

In addition, to assess the effectiveness of microgels on microbial growth, the growth curves of blank microgels (MGs) and MOS MGs were compared with those of the control group (Fig. 3C), which indicated that MOS MGs had no significant impact on bacterial growth. Since MOS is a carbon source that can be utilized by bacteria for their survival, we aimed to effectively trap MOS within the MGs to prevent its leakage and be further utilized by bacteria. To achieve this, we used a 20% concentration of GelMA with the expectation of effectively trapping MOS. To demonstrate the effective trapping of MOS within the microgels and its prevention from being utilized as a carbon source by bacteria, a series of tests were conducted.

First, the growth curves of bacteria were monitored in M9 medium, with MOS, MGs, and MOS MGs as the sole carbon source. In contrast to the MOS, the MGs group did not exhibit a discernible increase in AIEC growth (Fig. 3D). This indicates that the MOS in the microgels was not utilized as a carbon source by the bacteria. In addition, to simulate the effects of retention of MOS in microgels in vivo environments, we first immersed the microgels in simulated gastric fluid (SGF) and simulated intestinal fluid (SIF) and measured the environmental release of MOS using a UV-Vis spectrophotometer. The results indicated that there was no significant difference in the concentration of MOS in the supernatant compared to the control group (Fig. 3E). Furthermore, we also examined the morphological changes of the MGs in SGF and SIF. After soaking in SGF for 3 h and in SIF for 24 h, there were no significant morphological changes in the microgels compared to the control group (Fig. 3F–H). In all, this section of results demonstrated that the MOS MGs exhibit good biocompatibility and stability, not affecting the growth of cells and bacteria. Simultaneously, the MOS MGs effectively confine to the MOS, preventing it from being utilized as a carbon source for bacterial growth.

### MOS MGs block AIEC adhesion to intestinal epithelial cells by binding with FimH

**Fig. 4:**
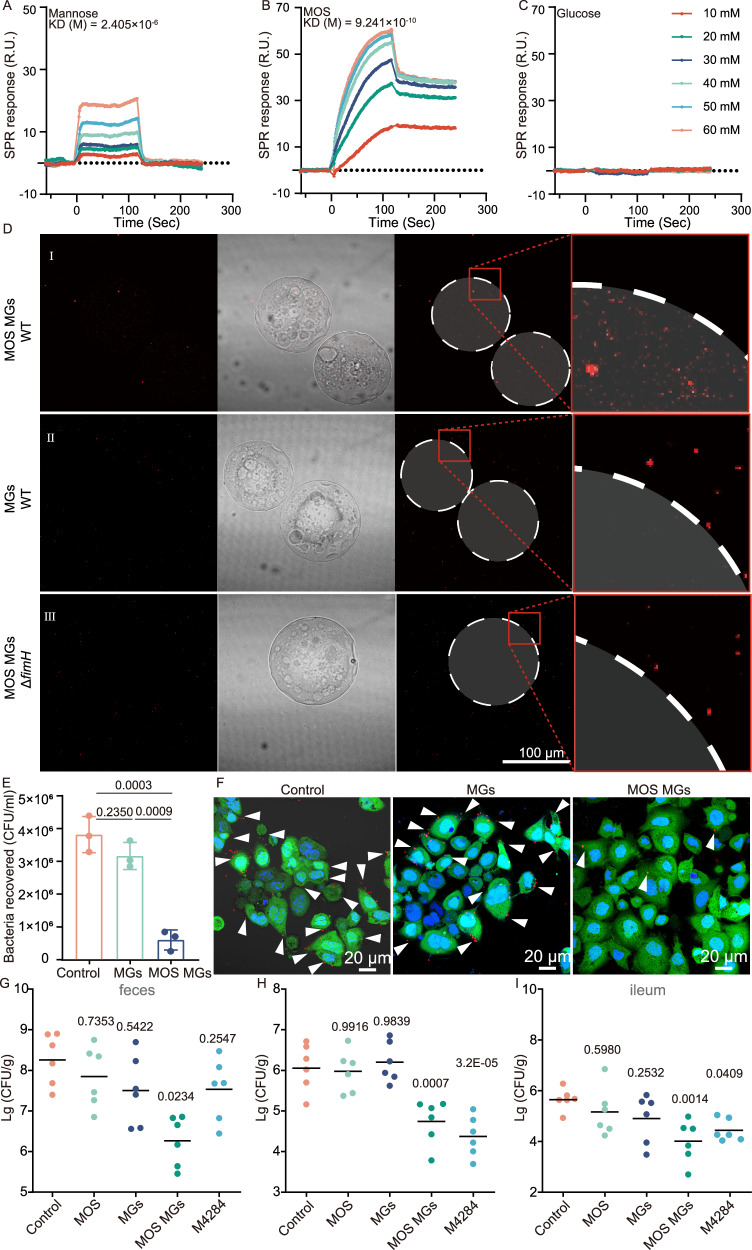
MOS MGs block AIEC adhesion to intestinal epithelial cells by binding with FimH. **A**–**C** The purified FimH protein was subjected to SPR experiments to detect its molecular interaction with mannose (**A**), MOS (**B**), and glucose (**C**). **D** Confocal images of MOS MGs (I) and MGs (II) co-cultured with AIEC, as well as confocal images of MOS MGs co-cultured with ΔfimH (III). **E** Competitive adhesion assay of AIEC between MOS MGs and Caco-2 cells, visualized by colony-forming unit (CFU) plating (*n *= 3 biological replicates). **F** Confocal imaging of competitive adhesion of AIEC between MOS MGs and Caco-2 cells. Red: AIEC; blue: DAPI-stained nuclei; green: Calcein-AM-stained cells; white arrows indicate AIEC. **G**–**I** Colonization levels of WT in feces (**G**), colon (**H**), and ileum (**I**) at 48 hours post-treatment with MOS, MGs, MOS MGs, and M4284. (*n* = 6). Significance was determined by one-way ANOVA (**E**) or two-sided Mann‒Whitney U test (**G**–**I**) and indicated as the *P*-value. * *P* < 0.05, ** *P *< 0.01, *** *P* < 0.001, **** P < 0.0001; n.s. no significant difference. Data are presented as mean ± s.d. (**E**). Source data are provided as a Source Data file.

Next, we investigated the adsorption capacity of the microgels towards AIEC. Initially, surface plasmon resonance (SPR) experiments were conducted to assess the binding affinity of glucose, mannose, and MOS to FimH. We set up three groups of experiments, in which glucose served as the negative control, mannose served as the positive control^22^, and MOS served as the experimental group. The results indicated that MOS exhibited stronger binding capability to FimH than mannose (Fig. 4A–C). Afterward, to further interpret the adsorption ability, WT was incubated with MOS MGs and MGs. Considering the injectability required for subsequent in vivo experiments and to ensure consistency between in vitro and in vivo studies, microgels with a particle size of 100–150 μm were selected for all subsequent experiments. The results showed that WT could be adsorbed by MGs loaded with MOS, whereas MGs without MOS did not adsorb WT (Fig. 4D-I, II). Similarly, we also checked the adsorption of Δ*fimH* with MOS MGs, when *fimH* is mutant, AIEC is no longer adsorbed by MOS MGs (Fig. 4D-III). In addition to microscopic observations, we also conducted a quantitative analysis of the adsorption efficiency. We co-cultured MOS MGs with both WT and Δ*fimH*, and performed plating for each combination. Similarly, we co-cultured WT with MOS MGs and MGs, followed by plating. The results, shown in Supplementary Fig. 7A and B, indicated that the adsorption capacity of MOS MGs for bacteria significantly decreased in the absence of *fimH*, while the microgels without MOS did not exhibit adsorption capability for the bacteria. These findings suggested that the adsorption of MOS MGs to AIEC is mediated by the interaction between the loaded MOS and FimH. To evaluate competitive adhesion inhibition, Caco-2 cells were co-incubated with AIEC in the presence of MOS MGs or MGs. Results from both plate counting (Fig. 4E) and confocal microscopy (Fig. 4F) consistently demonstrated that the number of bacteria adhering to the cell surface was significantly reduced in the MOS MGs group compared to the control group.

We further conducted in vivo experiments for verification using a DSS-induced mouse model of colitis. We established five experimental groups: a control group (administered AIEC by gavage following antibiotic treatment), a MOS treatment group, a MGs treatment group, an MOS MGs treatment group, and a positive control group (M4284^23^, a high-affinity inhibitory mannoside). Following treatment, fecal samples, colon, and ileum tissues were collected for bacterial enumeration using plate counting (Fig. 4G–I). No significant differences in bacterial colonization were observed in the MOS or MGs treatment groups compared to the control group. In contrast, treatment with M4284 led to a significant reduction of AIEC colonization in the ileum and colon, but not in the feces. Notably, mice treated with MOS MGs exhibited a significant decrease in AIEC levels in the ileum, colon, and feces. These results indicate that MOS MGs, beyond mimicking the anti-adhesive function of M4284, also exert an active bacterial-trapping effect, thereby promoting the effective clearance of AIEC from the intestinal tract. However, when Δ*fimH* was administered orally, there were no significant differences between the five groups (Supplementary Fig. 8). The results indicated that in vivo, MOS MGs adsorb AIEC by binding to FimH and reduce its colonization in the small intestine.

In conjunction with the previous results, we found that MOS MGs can securely immobilize MOS, preventing their release in the physiological environment, and competitively adsorb AIEC by binding with FimH, thereby achieving a therapeutic effect comparable to that of M4284.

### Therapeutic efficacy of MOS MGs in the mice model with DSS-induced colitis

**Fig. 5:**
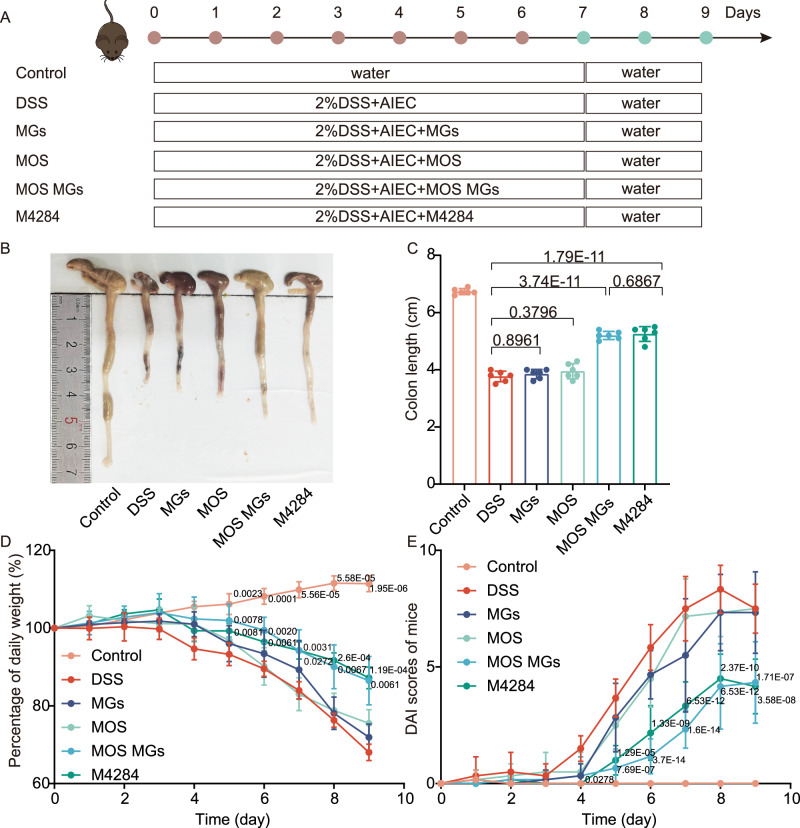
Excellent therapeutic efficacy of MOS MGs in the mice model with DSS-induced colitis. **A** Schematic illustration of model building and intervention. **B** Macroscopic colon appearance of each group was shown. **C** Colon length was measured and analyzed (*n *= 6). **D** Daily changes in body weight were recorded in detail and analyzed (*n* = 6). **E** Daily DAI scores were calculated and analyzed (*n* = 6). Significance was determined by one-way ANOVA (**C**), two-way ANOVA (**D**, **E**) and indicated as the *P*-value. Data are presented as mean ± s.d. (**C**, **D**, **E**). (*n* = 6 biological replicates). Each replicate represents data from an individual mouse. Source data are provided as a Source Data file.

**Fig. 6:**
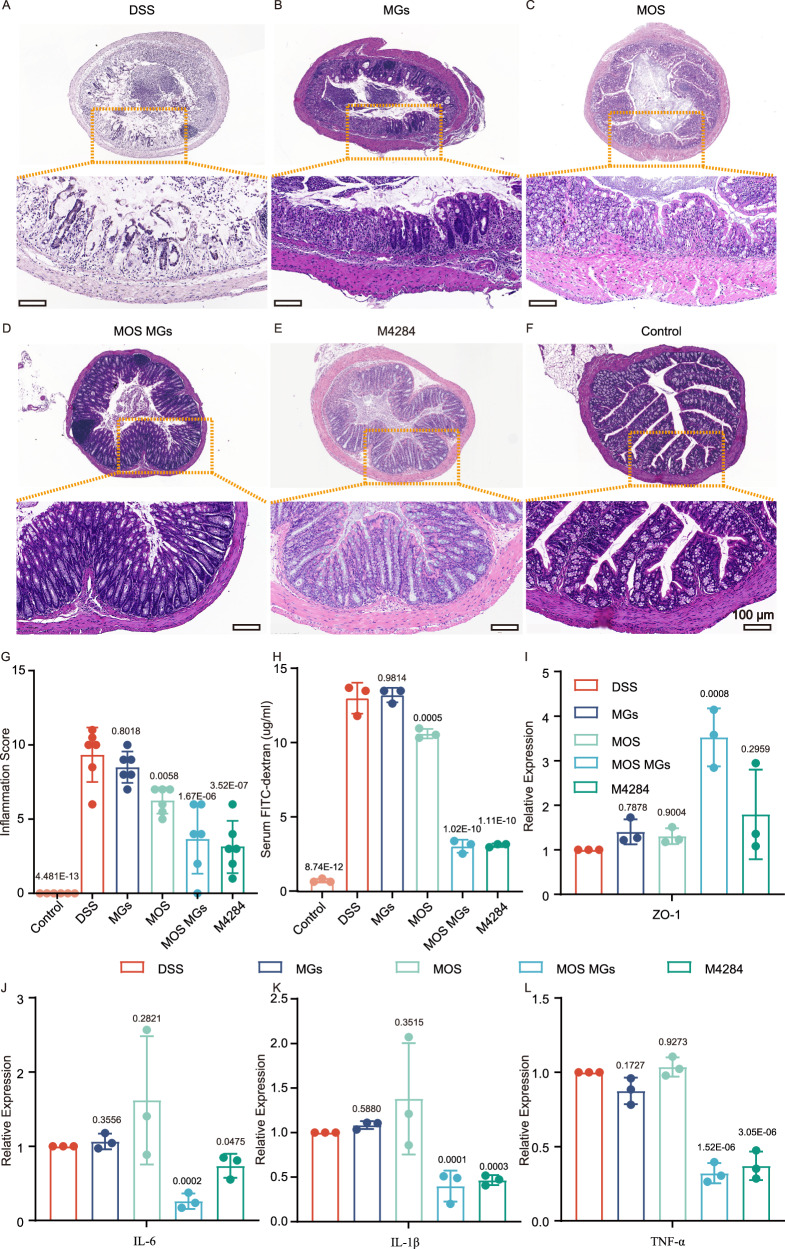
MOS MGs inhibit intestinal inflammation and prompt tissue repair. **A**–**F** Representative hematoxylin and eosin (H&E) staining images of colon tissue of each group. **G** Colonic damage scores according to H&E staining were analyzed in each group (*n* = 6 biological replicates). Each replicate represents data from an individual mouse. **H** Intestinal permeability assessment using FITC-dextran (*n* = 3 biological replicates). Each replicate represents data from an individual mouse. **I**–**L** Colonic mRNA levels of ZO-1, IL-6, IL-1β, and TNF-α, (*n* = 3 biological replicates). Significance was determined by one-way ANOVA (**G**–**L**) and indicated as the *P*-value; ns, not significant; * *p* < 0.05, ** *p* < 0.01, *** *p* < 0.001, **** *p* < 0.0001. Data are presented as mean ± s.d. Source data are provided as a Source Data file.

To further confirm the therapeutic effect of MOS MGs on AIEC-induced colitis in vivo, we proceeded to establish a colitis mouse model, the experimental protocol was presented in Fig. 5A. During the treatment process, we monitored changes in the colon length, body weight, gut bleeding (Supplementary Fig. 9A), stool consistency (Supplementary Fig. 9B), and disease activity index (DAI) data across six groups of mice. Mice treated with dextran sulfate sodium salt (DSS) showed significant weight loss, reduced intestinal length, and very high DAI, as expected^24^. There was no significant difference between the MGs group, MOS group and the DSS group, while both the M4284 group and the MOS MGs group showed varying degrees of therapeutic improvement. Of note, colitis model animals treated with MOS MGs or M4284 exhibited increased colon length than those treated with groups of MOS, MGs, and DSS (Fig. 5B, C) and significantly less weight loss (Fig. 5D), a lower DAI (Fig. 5E). We further verified the efficacy of MOS MGs by H&E staining of colon tissue. According to the colon damage score from the H&E staining in Fig. 6A–F, treatment of MOS MGs also helped maintain the integrity of colon epithelium and lessen the infiltration of pro-inflammatory cells of the mucosa, exhibiting a therapeutic effect comparable to that of M4284 (Fig. 6G).

Next, we subsequently conducted a detailed analysis to elucidate the mechanism by which MOS MGs protect mice from DSS-induced colitis. First, we evaluated whether MOS MGs could improve intestinal epithelial barrier function. To this end, we assessed intestinal permeability using Dextran-FITC and examined the expression of ZO-1. ZO-1 is a tight junction protein that forms tight connections between epithelial cells in the intestine, thereby regulating the permeability of the intercellular space. In patients with IBD, ZO-1 expression and function are often compromised, leading to disruption of the intestinal mucosal barrier and increased permeability to bacteria and toxins^25^. Compared to the DSS group, treatment with MGs led to a certain reduction in intestinal permeability. However, both the MOS MGs and M4284 treatment groups showed a marked decrease in permeability (Fig. 6H). Furthermore, mice treated with MOS MGs exhibited a significant upregulation of ZO-1 gene expression, indicating that MOS MGs effectively enhance intestinal epithelial barrier function (Fig. 6I).

Moreover, we selected IL-6, IL-1β, and TNF-α as targets for analysis and compared the gene expression levels of these three inflammatory factors in the colons of mice across four different treatment groups. IL-6, IL-1β, and TNF-α play crucial roles in intestinal inflammation by acting as pro-inflammatory cytokines that promote the initiation and persistence of inflammatory responses^26,27^. Dysregulated expression and modulation of these cytokines may contribute to the chronicity and exacerbation of inflammatory reactions. As expected, RT-PCR experimental results are consistent with our previous findings in vitro. Treatment with M4284 or MOS MGs significantly downregulated pro-inflammatory genes (IL-6, IL-1β, TNF-α) compared to the DSS group, with MOS MGs showing the most pronounced effect (Fig. 6J–L). In contrast, neither MOS nor MGs alone significantly altered these inflammatory factors relative to DSS controls.

Given that AIEC can both colonize in ileum and the colon, we further analyzed the effects of MOS MGs in the ileum of mice. RT-PCR analyses of IL-6, IL-1β, TNF-α and ZO-1 expression (Supplementary Fig. 9C–F) and H&E staining of ileal tissue sections (Supplementary Fig. 9G–M) were performed. The results showed trends consistent with those observed in the colon.

In conclusion, MOS MGs demonstrated a superior therapeutic effect in alleviating AIEC-induced colitis compared to MOS alone, and in certain aspects, even outperformed M4284.

### MOS MGs modulate of gut microbiome

To determine whether MOS MGs treatment altered the microbiome, we performed high-throughput gene-sequencing analysis of 16S rRNA in fecal bacterial DNA isolated from the control group, DSS group, MOS group, MGs group, and MOS MGs group mice.

**Fig. 7:**
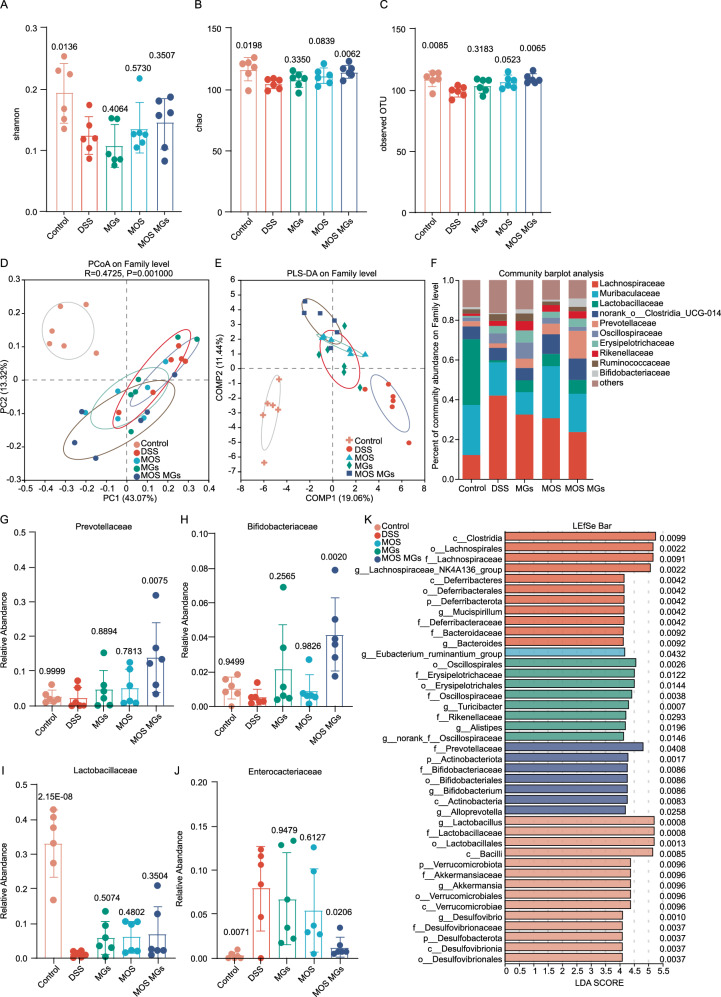
MOS MGs modulate of gut microbiome. **A**–**C** Shannon index (**A**), chao index (**B**), observed OTUs (**C**) of gut microbiota in mice after different treatments. OTUs operational taxonomic units. **D** Principal coordinate analysis (PCoA) using Bray-Curtis metric distances of beta diversity. Samples are colored based on treatment conditions. Each point represents a sample, with distances indicating the degree of dissimilarity between communities. PC1 and PC2 explain 43.07% and 13.32% of the variance, respectively. Bray-Curtis dissimilarities were calculated and subjected to classical multidimensional scaling for ordination. Significant differences between groups were assessed using PERMANOVA (permutational multivariate analysis of variance), performed as a two-sided test with 999 permutations (*p* < 0.05). **E** PLS-DA Analysis of 16S rRNA Sequencing Data. PLS-DA was performed to assess the differences in microbial community composition among samples grouped by treatment conditions. Each point represents a sample, and the clusters indicate significant differences in microbial communities. Partial least squares discriminant analysis was used as a supervised multivariate method. Statistical significance was evaluated using two-sided permutation tests (*n* = 1000 permutations). (*p* < 0.05). **F** Community barplot analysis of microbial composition. Barplots illustrate the relative abundance of microbial taxa across different samples, grouped by treatment conditions. Taxa are represented at the genus level, with colors indicating different taxonomic groups. Each bar represents the relative abundance of different taxa, with the total height indicating overall community composition. Relative abundance of *Enterobacteriaceae* (**G**), *Prevotellaceae* (**H**), *Lactobacillaceae* (**I**) and *Bifidobacteriaceae* (**J**) at family-level taxonomy after different treatments. **K** Taxa listed according to their LDA values determined from comparisons between the five groups using the LEfSe method. LDA (log10)  >  4.0, *P*  <  0.05 indicates a higher relative abundance in the corresponding group than in other groups. Statistical analysis was conducted using the LEfSe pipeline, which includes a two-sided non-parametric Kruskal–Wallis test to detect taxa with significant differences among groups, followed by an unpaired Wilcoxon rank-sum test to assess subclass consistency, and linear discriminant analysis (LDA) to estimate the effect size. No adjustment for multiple comparisons was applied. The number of samples is *n* = 6. Data are presented as mean ± s.d. Source data are provided as a Source Data file.

First, alpha diversity through different methodologies were analyzed. Although the Shannon index did not show difference between the DSS group, MOS group, GelMA group, and MOS MGs group. The Chao1 index and the Number of observed OTU index were significantly increased in the MOS GelMA group compared with the DSS group, indicating MOS MGs treatment effectively prevents dysbiosis in DSS-induced colitis in mice (Fig. 7A–C). To extend our understanding of the role of MOS MGs in microbiota diversity, a principal coordinate analysis (PCoA) using Bray-Curtis metric distance and Partial least squares discriminant analysis (PLS-DA) were performed to evaluate the similarity (β-diversity) of microbial community structure among groups. Notably, we observed a distinct clustering of microbiota composition for the five groups (Fig. 7D, E). These data indicated that MOS MGs treatment significantly altered the intestinal bacterial diversity.

Subsequently, we assessed the relative abundance of the gut microbiota in all available samples in each group. The variations at the family level indicated that MOS MGs treatment has a unique microbial composition compared to the DSS group (Fig. 7F), suggesting that MOS MGs treatment could reshape gut bacteria after DSS treatment. Furthermore, the abundance comparison of predominant families showed that MOS MGs treatment was characterized by a significantly increased relative abundance of *Prevotellaceae*, and *Bifidobacteriaceae* (Fig. 7G, H), when compared to the gut microbiota of the DSS treated group. MOS treatment significantly increased relative abundance of *Lactobacillaceae*, when compared to the gut microbiota of the DSS treated group, although MOS MGs treatment is not statistically significant, it still showed an upward trend (Fig. 7I). Meanwhile, treatment with MOS MGs significantly decreased the relative abundance of the *Enterobacteriaceae* family, which is commonly known for expressing type 1 fimbriae that mediate mannose-sensitive adhesion to host cells (Fig. 7J).

To confirm which bacterium was altered by MOS MGs treatment and, in turn, affected the disease progression against DSS-induced colitis, we performed high-dimensional class comparisons using the linear discriminant analysis (LDA) of effect size (LEfSe) that detected marked differences in the predominance of bacterial communities between five groups. Notably, the relative abundances of *Bifidobacteriaceae* and *Prevotellaceae* increased significantly. *Bifidobacteriaceae*, a well-established probiotic family, is widely recognized for its ability to restore the intestinal barrier and alleviate inflammation, supporting the therapeutic potential of MOS MGs^28,29^. In contrast, although *Prevotellaceae* is often associated to improved carbohydrate metabolism^30,31^ and potential benefits for gut health^32–34^, its role remains controversial, as certain strains have also been implicated in pro-inflammatory conditions depending on the host context and microbial interactions^35–37^. Despite this, the concurrent reduction in *Enterobacteriaceae*—a bacterial family closely linked to intestinal inflammation and dysbiosis—suggests that MOS MGs not only suppress pathogenic bacteria but also contribute to microbiome rebalancing and inflammation alleviation. (Fig. 7K).

In conclusion, the treatment of MOS MGs can significantly enhance the relative abundance of *Bifidobacteriaceae* and reduce the relative abundance of *Enterobacteriaceae*, thereby mitigating inflammation in DSS-induced colitis.

## Discussion

The escalating incidence and mortality rates associated with IBD underscore the urgent need for effective therapeutic strategies. The multifactorial etiology of IBD, involving the interplay of genetic, environmental, and microbial factors, creates a complex treatment landscape. Although significant progress has been made in the treatment of IBD, several challenges and limitations remain, which impact both treatment efficacy and patient quality of life. Traditional therapeutic approaches, such as immunosuppressants, corticosteroids, and biologics, while effective in alleviating symptoms, are associated with notable side effects^38^. For instance, immunosuppressants may suppress the immune system, increasing the risk of infections^39^; prolonged use of corticosteroids may lead to osteoporosis, weight gain, and hyperglycemia^40^; and biologics may trigger allergic reactions or the development of drug resistance^41^. These side effects not only increase the health burden on patients but may also affect their adherence to treatment. Furthermore, current therapies can disrupt the normal balance of the gut microbiome^42^. Long-term use of antibiotics or immunosuppressants can disrupt the gut microbiome balance, causing microbial dysbiosis and a reduction in beneficial bacteria, which leads to immune system dysregulation and intensifies inflammatory responses, ultimately exacerbating disease progression. Therefore, developing new therapeutic strategies, particularly those that are more precise, personalized, and capable of reducing side effects, remains a critical goal in the field of IBD treatment.

To address the limitations of current therapies, researchers have begun exploring more targeted approaches. Compelling evidence suggests that AIEC is closely associated with the pathogenesis of CD, and given its pivotal role in inflammation, FimH, a key adhesin of AIEC, has emerged as a therapeutic target for IBD, gaining increasing attention in the field. Some studies have attempted to develop inhibitors targeting FimH, such as small molecule antagonists or antibodies, aiming to block the interaction between FimH and gut cells. These studies suggest that inhibiting FimH activity could effectively alleviate symptoms in IBD patients and reduce common side effects of current treatments, such as immune system suppression and dysbiosis^18,43^. However, the pharmacokinetics of FimH inhibitors remain poorly understood, including their absorption, metabolic stability, and excretion. Moreover, as most FimH inhibitors are mannoside-based, they may be metabolized by the gut microbiota as a carbon source.

To overcome the limitations of current FimH-targeted therapies, we developed an oral microgel-based engineering carrier for MOS delivery and immobilization. Microgels offer robust protection for MOS against degradation in the harsh gastric environment and prevent its premature metabolism by the gut microbiota as a carbon source, thereby reducing the risk of microbial dysbiosis. The microgels ensure that MOS is delivered intact to the intestinal tract, where it exerts its therapeutic effects and is ultimately excreted without systemic absorption. Importantly, MOS MGs demonstrated therapeutic efficacy comparable to commercially available FimH inhibitors such as M4284. However, unlike traditional inhibitors that merely block AIEC adhesion to intestinal epithelial cells, MOS MGs not only inhibit adhesion but also physically trap and remove adherent AIEC from the gastrointestinal tract, resulting in more effective bacterial clearance.

To implement this oral delivery strategy, we employed an innovative fabrication approach that integrates an ATPS-based strategy with microfluidic technology. In contrast to conventional oil-phase microfluidic methods, this ATPS-based strategy eliminates the use of organic solvents and surfactants, significantly enhancing the biosafety and in vivo applicability of the microgels. The resulting porous microgel exhibits stable structures, precise tunability, and high biocompatibility, providing an ideal platform for MOS encapsulation and targeted intestinal delivery, thereby enhancing its potential for clinical translation.

Moreover, the targeting mechanism of this microgel platform offers broad therapeutic potential, as FimH is a highly conserved adhesin found in a variety of pathogenic bacteria, including *Enterobacter*, *Klebsiella*, *Salmonella*, *Shigella*, and uropathogenic *E. coli*. By interfering with FimH-mediated adhesion, this system has significant potential as a localized, microbiota-friendly, and low-resistance therapeutic alternative for a range of FimH-expressing bacterial infections. Future studies should aim to further optimize the structure of microgels for enhanced targeting efficiency, evaluate potential synergies with existing treatments, and comprehensively assess the effects of this system on mucosa-associated microbiota, ultimately contributing to the development of precise and personalized IBD therapies.

## Methods

### Preparation of MOS MGs

#### Synthesis of GelMA

20 g of gelatin (Sigma-Aldrich CAS:9000-70-8) were dissolved in 200 mL of PBS and stirred at 60 °C at 36 × g. Subsequently, 16 mL of maleic anhydride (MA, Sigma-Aldrich CAS:108-31-6) was added to the gelatin solution using a microfluidic syringe pump at a flow rate of 0.25 mL/min. After reacting for 2 h, 800 mL of pre-warmed PBS was added to stop the reaction. The mixture was then transferred to a dialysis bag with a molecular weight cut-off of 8000–14,000 Da and dialyzed for 1 week. The dialyzed solution was filtered through a 0.22 µm microporous membrane at 60 °C. The filtrate was stored at − 80 °C overnight and then subjected to vacuum freeze-drying for 7 days to obtain the hydrogel material under dry conditions.

#### Synthesis of FITC-Conjugated GelMA

The GelMA used for fluorescence imaging in this study was grafted with FITC. The same GelMA was conjugated to the amine group using the isothiocyanate group of fluorescein isothiocyanate isomer I (FITC), resulting in a stable thiourea derivative under physiological pH conditions. To label GelMA, 0.4 g of GelMA was dissolved in 10 mL of PBS. Four milligrams of FITC (Sigma-Aldrich, St. Louis, MO, USA) were dissolved in 0.4 mL of dimethyl sulfoxide (Sigma-Aldrich) and then mixed into the GelMA solution. The reaction was maintained at 37 °C in the dark overnight. Excess FITC was removed via dialysis at room temperature for one week. The resulting labeled GelMA was then freeze-dried^44^.

#### Preparation of MOS MGs

The ATPS-based strategy, combined with microfluidic techniques, was employed for generating MOS MGs. The microfluidic device consisted of a coaxial needle with an inner diameter of 23 G and an outer diameter of 14 G, along with a collection tube featuring an inner diameter of 1.8 mm. The inner phase was composed of a mixture containing 20% (w/v) GelMA, 1% (w/v) PEO (Sigma-Aldrich, 182001-500 G), and 50% (w/v) MOS (TX21112-500g). Upon injecting the dispersed phase and the continuous phase (20% w/v dextran, (Sigma-Aldrich, 31389-25 G)) into the device, the resulting droplets were exposed to UV light and solidified in the collection dish. The microspheres were then washed with Milli-Q water to remove the dextran.

### Characterization of MOS MGs

#### The morphology of MOS MGs

The optical and fluorescence images of MOS MGs were obtained by fluorescence microscopy (Nikon Corporation, ECLIPSE Ti2-E; MSHOT MF53-N) and a laser scanning confocal microscope (ELYPA P.1, ZEISS, Germany). For Fig. 2B, to enhance morphological visualization, we incorporated red fluorescent nanoparticles (R-PS3μm-R, Ruixibio) into the GelMA. The diameter of MOS MGs was measured by ImageJ software (1.53e; Java 1.8.0_172). The freeze-dried microgels were observed by scanning electron microscopy (SEM) (Phenom pure, Thermo, USA) for the surface appearance. The images were magnified 1000 times to observe the surface morphology of the hydrogel, including pore size and porosity, on at least three occasions.

#### Mechanical testing of MOS MGs

Microgels with a diameter of 500 µm were prepared using the ATPS-based strategy, single emulsion technique. A Texture Analyzer (TA.XT PlusC) was employed to compress the microgels at a rate of 0.01 mm/s with a strain of 50%. After completing the stress compression test, the probe remained stationary for 300 s to record stress changes during this period. In cyclic compression experiments, the Texture Analyzer was used to perform two cycles of compression and release with a strain of 50%. Elasticity was calculated based on the height change of the microspheres after the second compression. Cohesiveness was determined as the ratio of the area under the curve during the second compression to that during the first compression. Resilience was calculated as the ratio of the work done during the first compression to the total work done during the second compression.

### Bacterial strains, plasmids, and growth conditions

The bacterial strains and plasmids used in this study are listed in Supplementary Table 1 and 2. Mutant strains were generated using the λ Red recombinase system^45^, with all strains verified by PCR amplification using PCR mix(A019, GenStar, Beijing, China) and sequencing. For protein overexpression and purification, fimH was amplified, purified using the SPARKeasy Gel DNA Extraction Kit (AE0101, Shandong Sparkjade Biotechnology Co., Ltd.) and cloned into the pET28a expression plasmid and transformed into E. coli BL21 via heat shock transformation^46,47^. All constructs were confirmed by DNA sequencing. Primers used in the experiments are listed in Supplementary Table 2. Bacterial cultures were grown at 37 °C in Luria–Bertani (LB) broth. Antibiotics were added as needed at the following final concentrations: ampicillin, 100 µg mL^−1^; chloramphenicol, 15 µg mL^−1^; and kanamycin, 50 µg mL^−1^.

### In vitro biocompatibility on MOS MGs

To assess cell proliferation on hydrogel materials, the cell counting kit-8 (CCK-8) assay was employed. MOS MGs were incubated in DMEM for 1, 4, and 7 days to obtain leaching solutions. Caco-2 cells (SUNNCELL) and HeLa cells were seeded in a 96-well plate at a density of 10,000 cells/well, cultured with the leaching solution for 1, 4, and 7 days, and subjected to CCK-8 incubation at 37 °C for 40–90 min. Absorbance was measured at 450 nm using a microplate reader.

For evaluating the toxicity of MOS MGs, samples were placed on round glass slides at the bottom of a 12-well plate, Caco-2 cells and HeLa cells were seeded in the plate. Live/Dead staining was performed on days 1, 4, and 7. Images were captured using fluorescence microscopy (ZEISS, Germany) with excitation at 488 nm and 568 nm; green fluorescence indicated live cells and red fluorescence indicated dead cells, allowing for assessment of cell number and viability.

To investigate the effect of MOS MGs on bacterial growth, the growth curve analysis was conducted. AIEC was cultured in 20 mL of LB medium with 1% MGs added and incubated at 37°C with shaking at 180 rpm for 20 h. The absorbance at 600 nm was measured hourly. All experiments were performed independently in triplicate.

### In vitro MOS release assay

MOS MGs were immersed at 37 °C in 2 mL SGF (pH = 1.5) for 3 h, 2 mL SIF (pH = 6.8) for 24 h, and 2 mL PBS (pH = 7.4) for 24 h, respectively. Determine the concentration of MOS released into the solution using a UV-Vis spectrophotometer. The morphology of MGs was examined by confocal laser scanning microscopy (CLSM).

To determine whether the release of MOS from microspheres affects AIEC growth, the growth curve analysis was conducted. AIEC was cultured in 20 mL of M9 medium with the addition of 0.5% MOS and 1% MOS MGs and incubated at 37 °C with shaking at 180 rpm for 20 h. Absorbance at 600 nm was measured hourly. Each experiment was performed independently in triplicate.

### Detection of bacteria adsorbed by MOS MGs in vitro

The WT and ΔfimH strains were incubated in 1% microgel solution for 24 h. The solution was then centrifuged at 900 × *g* for 5 min at 37 °C. The supernatant was discarded, and the microgels were washed five times with PBS, discarding the supernatant after each wash. The microgels were resuspended in PBS. For enumeration, 100 μL of the microgel solution was spread on LB agar and incubated overnight at 37 °C. To assess bacterial and microsphere colocalization, confocal microscopy was performed.

### In vitro cell viability and spreading on MOS MGs

To investigate the effects of fabrication methods and microgel morphologies on cell viability and spreading, human umbilical vein endothelial cells (HUVECs) encapsulated within microgels were subjected to Live/Dead cell staining and F-actin staining. HUVECs were homogeneously suspended at a density of 1 × 10^6^ cells/mL in a 15% (w/v) GelMA solution. Homogeneous and porous microgels were subsequently fabricated via oil-shearing microfluidics and the ATPS-based strategy, respectively. On days 1, 4, and 7 of culture, encapsulated HUVECs were stained with Calcein-AM/PI (to assess viability) and phalloidin (to visualize F-actin cytoskeleton). For F-actin staining, nuclei were counterstained with DAPI, with red and blue fluorescence corresponding to F-actin and nuclei, respectively. Fluorescence images were acquired via CLSM (ZEISS, Germany) using excitation at 488, 568, 581, and 405 nm.

### Cell adhesion assay

Caco-2 cells (5 × 10^4^ cells/well) were seeded into 24-well plates and cultured in a CO_2_ incubator at 37 °C until reaching 90% confluency. Bacteria were cultured to an optical density at OD_600_ = 1.0. Following two washes with PBS, cells were replenished with fresh DMEM medium. Bacteria were then added at a multiplicity of infection (MOI) of 1:10, with MGs or MOS MGs precipitates at volumes of 200 μL, respectively. After 3 h incubation at 37 °C, samples underwent five gentle PBS washes.

To quantify bacteria without adhesion to Caco-2 cells, washed cells were lysed with 0.1% Triton X-100 for 2 min. The resulting lysate was centrifuged at 13,000 × *g* for 1 min to pellet bacteria, followed by two PBS washes of the bacterial pellet. The bacterial pellet was resuspended in 1 mL PBS, plated onto LB agar plates, and incubated for 24 h at 37 °C, after which colonies were enumerated.

Following washing, Caco-2 cells were stained with Calcein-AM and Hoechst 33342. For bacterial visualization, pUC57-Tac-mCherry plasmids were transfected. Fluorescence imaging was performed via CLSM (ZEISS, Germany) using excitation of 488, 405, and 561 nm.

### Surface plasmon resonance screening

Ligand binding and binding kinetics analyses were conducted at 25 °C using a BIAcore X100 (BR110073). All experiments utilized PBS as the running buffer with a constant flow rate of 10 μL/min. FimH, diluted to a final concentration of 10 μM in 10 mM sodium acetate buffer (pH = 5.0), was immobilized on a CM5 Sensor Chip surface via EDC/NHS cross-linking according to the manufacturer’s instructions. The target immobilization level for FimH was set to 3600 RU. Small molecules, diluted in the running buffer from 0.75 to 12.5 mM, were injected into both the reference and FimH channels at a flow rate of 10 μL/min. The association and dissociation times were both 120 s. The Biacore X100 evaluation software was used to fit the affinity curves using the steady-state affinity model (1:1), and the equilibrium dissociation constant (KD) was calculated.

### Ethics statement

All animal experiments were performed according to the standards set forth by the Guide for the Care and Use of Laboratory Animals. The experimental protocols were approved by the Institutional Animal Care Committee of Nankai University.

### Animals

Seven to eight-week-old specific pathogen-free (SPF) female C57BL/6 J mice were purchased from Beijing Vital River Laboratory Animal Technology (Beijing, China). Mice were maintained under specific pathogen-free (SPF) conditions with a 12 h light/dark cycle (lights on at 7:00 a.m.), a temperature of 22 ± 2 °C, and relative humidity of 50–60%. Animals had free access to autoclaved drinking water and a standard rodent chow (SPF-F02-002), which contains approximately 18% crude protein, 5% crude fat, and 5% crude fiber.

### Mouse infection

Seven to eight-week-old SPF female C57BL/6 J mice were orally pretreated with streptomycin (20 mg intragastric per mouse) to disrupt intestinal microbiota. Twenty-four hours later, the mice were challenged with 10^9^ AIEC bacteria. After 48 h, fresh feces and intestinal tissues were collected and homogenized in PBS. Bacteria were numbered by plating appropriate dilutions on LB agar medium containing the appropriate antibiotics to select and isolate AIEC bacteria and incubated overnight at 37 °C.

### Induction of colitis

Chemically-induced colitis was established by providing the mice with 2.0% (w/v) DSS (MP Biomedicals, Santa Ana, CA, USA, molecular weight of 36,000–50,000) dissolved in drinking water for 7 days, followed by regular drinking water for the next 2 days^48^. Starting from the first day of the DSS challenge, 0.5% MOS, 1% MGs, 1% MOS MGs (containing 0.5% MOS) or M4284 (100 mg/kg), along with AIEC, were administered to mice via oral gavage at the indicated dose (CFU = 2 × 10^9^/mouse/day) in a total volume of 100 μL per mouse. Furthermore, 10 a.m. was chosen as a set time for feeding, with a duration of 7 days.

### Assessment of colitis severity

During the DSS treatment cycles, the DAI score was assessed to evaluate the progression of colitis^49^. The DAI was a composite score determined by factors such as relative body weight loss, stool softness, and blood in the rectum or stool^50^. The DAI was calculated based on body weight change, stool consistency, and gut bleeding^49^. Body weight loss was scored as follows: score 0, no weight loss compared to initial weight; 1, weight loss within 1–5%; 2, weight loss within 5–10%; 3, weight loss within 10–15%; 4, greater than 15% weight loss. Stool consistency was determined as follows: score 0, normal; 1, slightly loose stool; 2, loose stool; 3, diarrhea. Rectal bleeding was scored as follows: score 0, normal; score 1, small presence of blood; 2, significant presence of blood; 3, gross bleeding.

### Histological analysis

The small intestines of infected mice were collected for analysis. The small intestine was fixed in 10% neutral buffered formalin overnight and embedded in paraffin. Paraffin-embedded sections were deparaffinized and stained with hematoxylin-eosin. Then, the stained sections were blindly scored for inflammation severity. Colonic histological damage was scored based on the previously published paper. Damage was scored on a scale from 0 to 6, where 0 indicated no damage, 1 represented hyperproliferation with irregular crypts and loss of goblet cells, 2 denoted mild to moderate crypt loss (10–50%), 3 indicated severe crypt loss (50–90%), 4 signified complete crypt loss with intact surface epithelium, 5 reflected small to medium-sized ulcers (less than 10 crypt widths), and 6 represented large ulcers (greater than or equal to 10 crypt widths). Inflammatory cell infiltration was assessed separately for the mucosa, submucosa, and muscle/serosa. The mucosa was scored as 0 for normal, 1 for mild, 2 for moderate, and 3 for severe infiltration. The submucosa was scored as 0 for normal, 1 for moderate to mild infiltration, and 2 for severe infiltration. The muscle/serosa layer was scored as 0 for normal and 1 for moderate to severe infiltration. Scores for epithelial damage and inflammatory cell infiltration were summed, resulting in a total score of 0–12^51^.

### Intestinal permeability assay

Mice were deprived of food and water for 12 h and then orally administered FITC-dextran (average molecular weight 3000-5000; 60 mg per 100 g body weight). After 4 h, blood was collected. Serum fluorescence intensity was measured using a Spark 10 M multimode microplate reader (Tecan, Switzerland) at excitation and emission wavelengths of 485 nm and 525 nm, respectively. Serum FITC-dextran concentration was determined using a standard curve.

### RNA isolation and RT-PCR

To detect gene expression in vivo, collect mouse ileum and colon samples and gently scrape off contents. RNA was purified via precipitation with lithium chloride^52^. Next, the total RNA concentration was determined using a NanoDrop 2000 spectrophotometer (Thermo Fisher Scientific, MA, USA). Three independent experiments were performed. cDNA was synthesized using a PrimeScript™ RT Reagent Kit with gDNA Eraser (Takara, Kusatsu, Japan) according to the manufacturer’s instructions. RT-PCR analysis was conducted with an Applied Biosystems ABI 7500 (Applied Biosystems, Waltham, MA, USA) using SYBR Green fluorescence dye. To normalize sample data, the GAPDH gene was used as a reference control, and relative expression levels were calculated as fold change values using the 2^−ΔΔCT^ method. Each experiment was performed in triplicate.

### DNA extraction, bacterial 16S rRNA gene sequencing, and raw data processing

Total DNA was extracted from frozen stool samples using the FastPure Feces DNA Isolation Kit (MJYH, Shanghai, China), following the manufacturer’s protocols. DNA quality, including integrity, purity, and concentration, was assessed by 1% agarose gel electrophoresis and Nanodrop spectrophotometry (Nanodrop Technologies Inc., Wilmington, DE, USA). The DNA samples were diluted to a concentration of 2 ng/μL for PCR amplification.

The V3-V4 region of the bacterial 16S rRNA gene was amplified using the primer pair 338 F (5′-ACTCCTACGGGAGGCAGCA-3′) and 806 R (5′-GGACTACHVGGGTWTCTAAT-3′). PCR reactions were conducted in triplicate using a Nexseq 2000 (Illumina, USA). The thermocycling conditions were: initial denaturation at 95 °C for 3 min; followed by 30 cycles of denaturation at 95 °C for 30 s, annealing at 55 °C for 30 s, and extension at 72 °C for 45 s; with a final elongation at 72 °C for 10 min.

Triplicate PCR products were combined and purified using the ENZA Gel Extraction Kit (Omega Bio-Tek Inc., Norcross, GA, USA). Sequencing libraries were prepared using the NEXTFLEX Rapid DNA-Seq Kit (Bioo Scientific, USA) according to the manufacturer’s recommendations. Operational taxonomic units (OTUs) were clustered at a 97% similarity cutoff using VSEARCH (version 2.4.2), and representative reads for each OTU were selected with the QIIME package (version 1.8.0). Representative reads were annotated and classified against the SILVA 138/16s_bacteria using the RDP Classifier (confidence threshold set at 70%).

Microbial diversity in the intestine samples was estimated using α-diversity metrics, specifically the Shannon index. Unweighted UniFrac principal coordinate analysis was performed using the UniFrac distance matrix generated by QIIME software.

### Statistical analyses

No statistical methods were used to pre-determine sample sizes, but our sample sizes are similar to those reported in previous publications^53^. Animals were randomly assigned to either the control or manipulation groups, with data collected accordingly. The experimenters conducting the tests were blind to the experimental conditions of the mice. Data collection for other variables was not randomized but was consistently performed alongside the control group. No animals or data points were excluded from the study.

Data were analyzed using *t* tests, two-way ANOVA or Mann–Whitney U tests as indicated in the specific figure legends. Values with *P* < 0.05, 0.01, 0.001, or 0.0001 were considered statistically significant (*), highly significant (**), very high significance (***), or extremely high significance (****), respectively; n.s. represents no significance. Data distribution was assumed to be normal, but this was not formally tested. Figures were drawn using GraphPad 10.0.5.

### Reporting summary

Further information on research design is available in the Nature Portfolio Reporting Summary linked to this article.

## Supplementary information

Supplementary Information

Reporting Summary

Transparent Peer Review file

## Source data

Source Data

## Data Availability

The 16S rRNA gene sequencing data generated in this study have been deposited in the NCBI Sequence Read Archive (SRA) under accession code PRJNA1182980 (https://www.ncbi.nlm.nih.gov/bioproject/PRJNA1182980). All other data supporting the findings of this study are provided in the Source Data file accompanying the manuscript. Source data are provided in this paper.
