# Supplementary material for: Fishing out AIEC with FimH capturing microgels for inflammatory bowel disease treatment
Source: Nat Commun. 2025 Aug 25;16:7924. doi: 10.1038/s41467-025-63276-7 (PMC12378211; doi:10.1038/s41467-025-63276-7)
Supplement: Supplementary file 1 — Supplementary Information [file 41467_2025_63276_MOESM1_ESM.pdf]

Supplementary Information

Fishing Out AIEC with FimH Capturing Microgels for Inflammatory Bowel Disease Treatment

Jialin Wu[1,#], Yutao Liu[2,6,#], Ruiying Liu[2,#], Changyi Xiao[1], Leyan Xuan[1], Lili Wu[1], Jiamin Qian[2], Xudong Qin[1], Yingying Hou[5], Maobin Xie[5], Xiyong Yu[1,*], Bin Liu[2,3,4,*] and Guosheng Tang[1,*]

1. Guangzhou Municipal and Guangdong Provincial Key Laboratory of Molecular Target & Clinical Pharmacology, the NMPA and State Key Laboratory of Respiratory Disease, School of Pharmaceutical Sciences and the Fifth Affiliated Hospital, Guangzhou Medical University, Guangzhou, Guangdong 511436, P. R. China.
2. National Key Laboratory of Intelligent Tracking and Forecasting for Infectious Diseases, TEDA Institute of Biological Sciences and Biotechnology, Nankai University, Tianjin 300457, P. R. China;
3. Key Laboratory of Molecular Microbiology and Technology, Nankai University, Ministry of Education, Tianjin 300457, P. R. China;
4. Nankai International Advanced Research Institute, Shenzhen 518045, P. R. China
5. The Fourth Affiliated Hospital of Guangzhou Medical University; School of Biomedical Engineering, Guangzhou Medical University, Guangzhou 511436, P. R. China
6. School of Life Sciences, Faculty of Medicine, Tianjin University, Tianjin 300072, China;

[#] **Jialin Wu**, **Yutao Liu,** and **Ruiying Liu** contributed equally to this work.

* To whom correspondence may be addressed. E-mail: guoshengtang@gzhmu.edu.cn, liubin1981@nankai.edu.cn, or yuxycn@aliyun.com

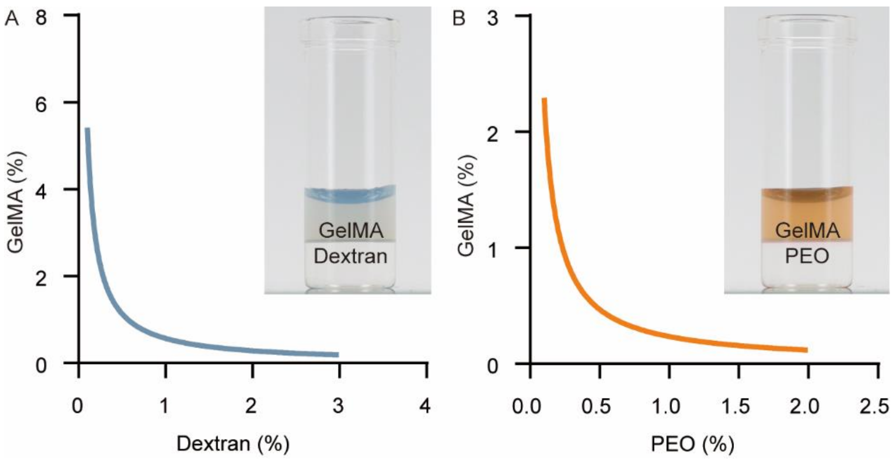

Supplementary Figure 1

A, B) Phase diagrams and stratification images of the GelMA with dextran (A) and GelMA with PEO (B) aqueous two-phase systems.

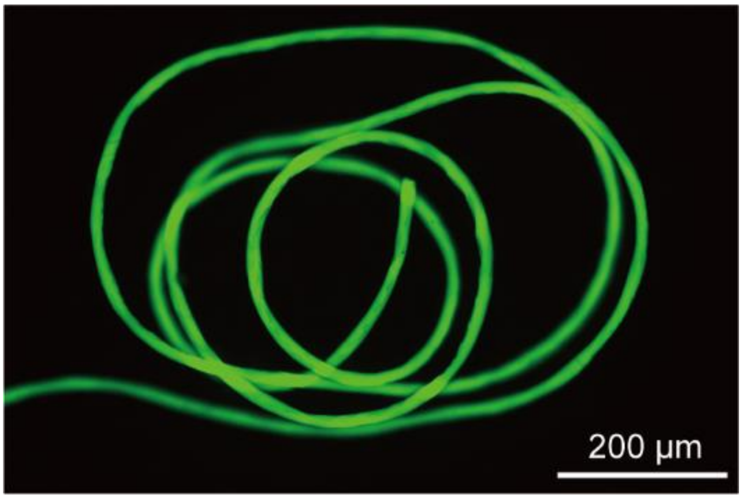

Supplementary Figure 2

Confocal image of fabricated filaments.

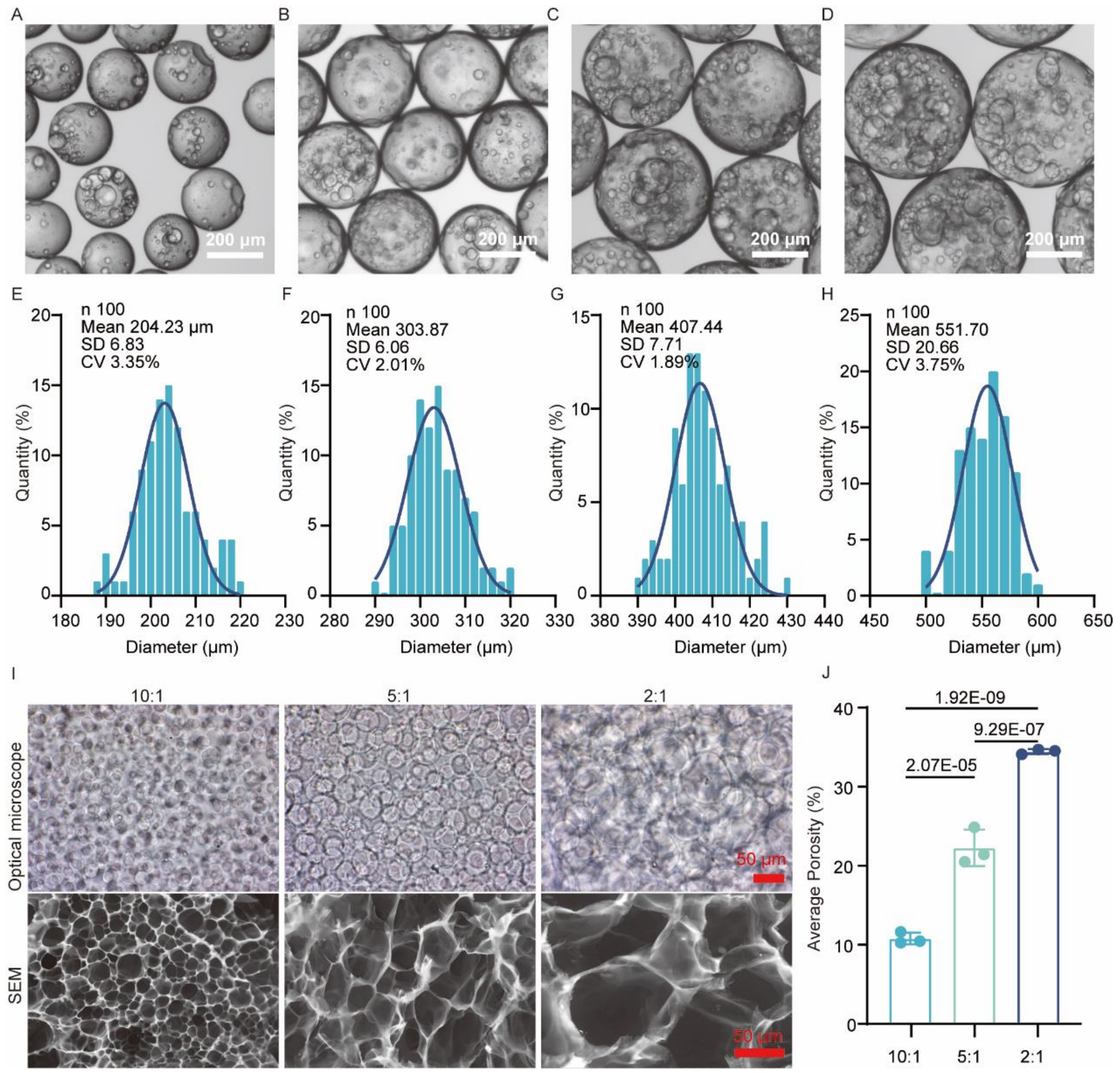

Supplementary Figure 3

A-H) Bright-field microscope images (A-D) of MOS MGs with different particle sizes and the corresponding particle size distribution graphs (n=100) (E-H). I–J) Bright-field and SEM images (I) of porous MGs with different porosities obtained by varying the volume ratio of GelMA to PEO, and the corresponding porosity quantification graph (J) (n = 3). Significance was determined by one-way ANOVA and indicated as the P value. *** $P < 0.001$. Data are presented as mean ± s.d.

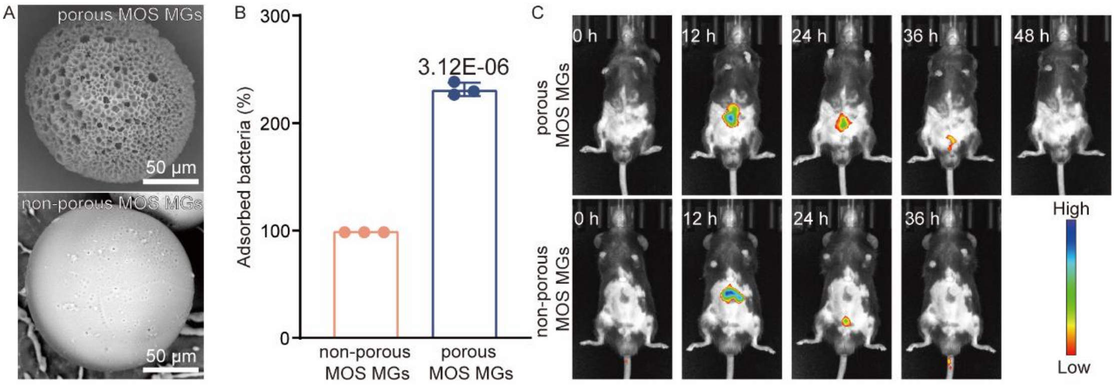

Supplementary Figure 4

A) SEM microscopy images of porous and non-porous MOS MGs. B) Colony counts of AIEC after co-culturing with porous and non-porous MOS MGs for 24 h (n=3). Colony formation was assessed following plating. Significance was determined by two-tailed unpaired Student's t test and indicated as the P value. **** $p < 0.0001$. Data are presented as mean ± s.d. C) IVIS imaging of representative mice at different time points following oral administration of porous or non-porous MOS MGs.

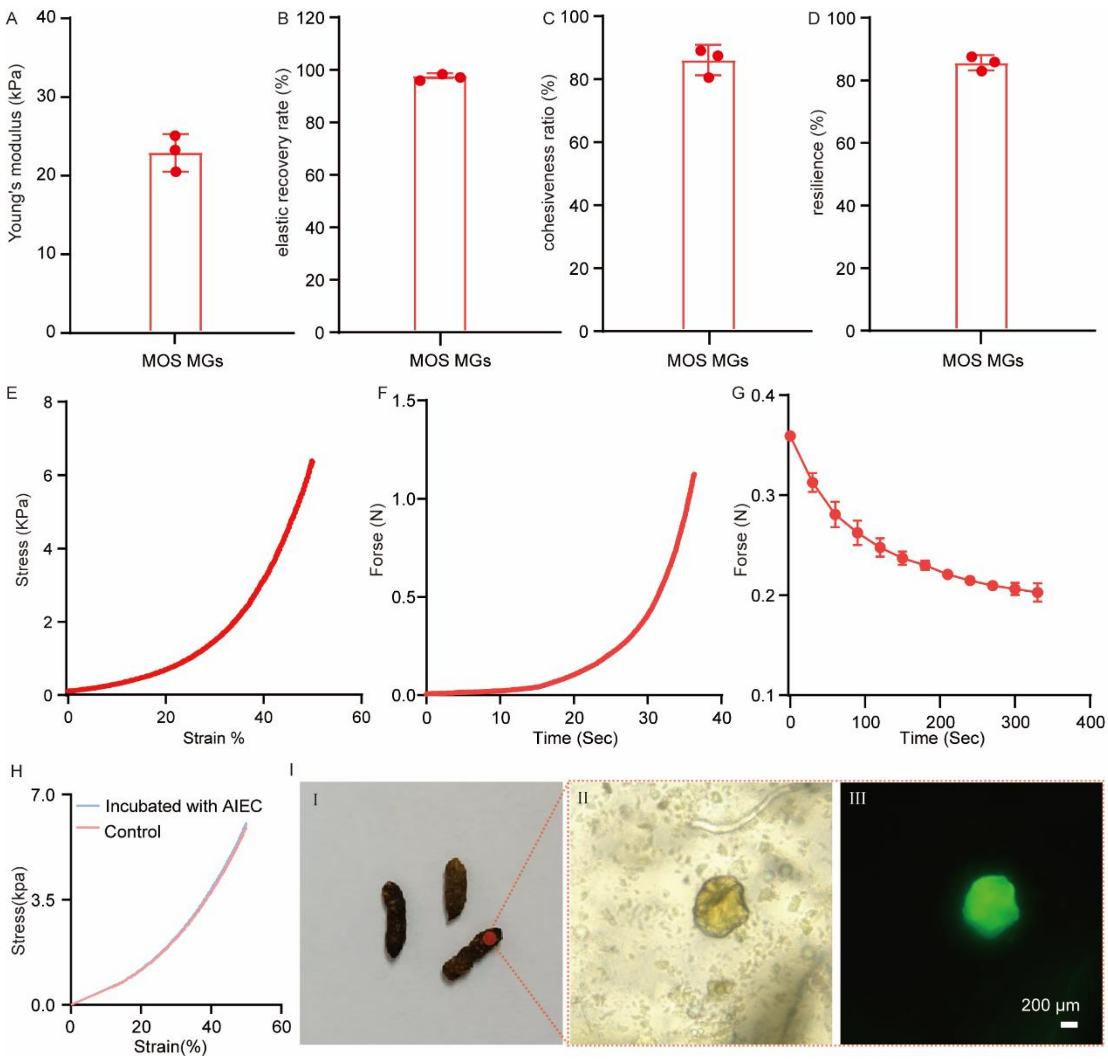

Supplementary Figure 5

A-D) Young's modulus analysis (A), elastic recovery rate (B), cohesiveness ratio (C), and resilience (D) of MOS MGs. E) Stress–strain curves for MOS MGs. F) Compression curve of a single MOS MGs. G) Stress relaxation curve of MOS MGs. H) Stress–strain curves of MOS MGs before and after 24 h co-incubation with AIEC. I) Bright-field and fluorescence images of MOS MGs recovered from mouse feces after administration. The number of samples is n = 3 (A-D, G).

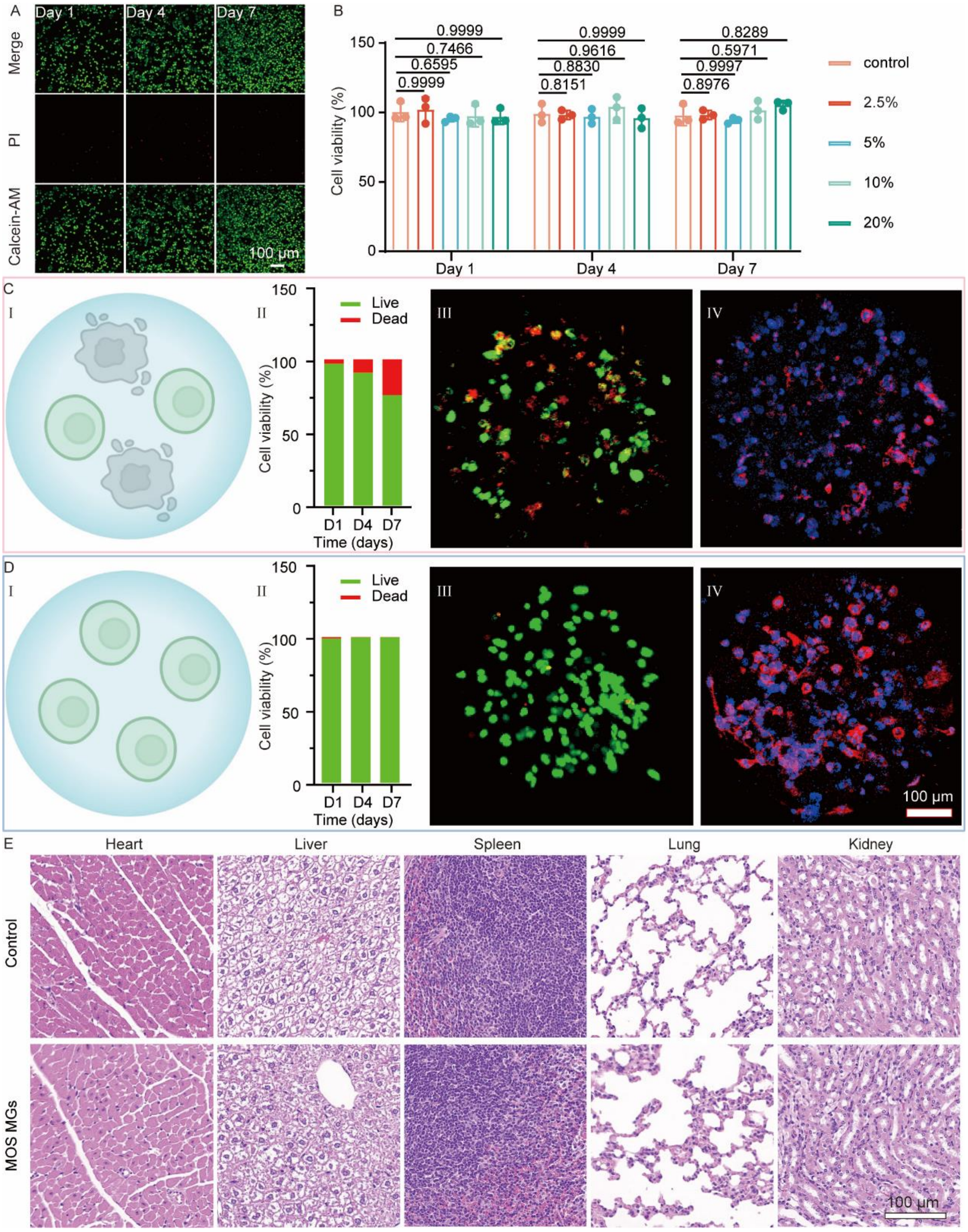

Supplementary Figure 6

A, B) Biocompatibility assessment of MOS MGs using HeLa cells: Live/Dead cell staining (A) and CCK-8 assay (B) (n=3). C–D) Comparative biocompatibility analysis of different cell-laden microgels: homogeneous microgels prepared via conventional oil-phase microfluidics (C), and homogeneous microgels prepared by ATPS-based microfluidics (D). For each group: I. Schematic illustration; II. Quantification of Live/Dead staining at days 1, 4, and 7; III. Confocal images of Live/Dead staining on day 7; IV. Confocal images of F-actin staining. E) H&E staining of the major organ

sections from C57BL/6 at day 21 under control and treatment with MOS MGs. Significance was determined by one-way ANOVA (B) and indicated as the P value. n.s., no significant difference. Data are presented as mean ± s.d. (B).

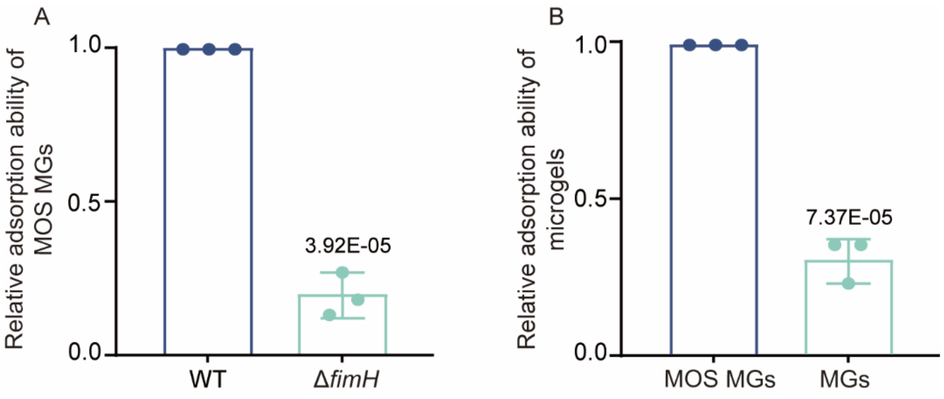

Supplementary Figure 7

A) Colony counts after co-culturing MOS MGs with AIEC and Δ*fimH* (n=3). B) Colony counts after co-culturing AIEC with MOS MGs and MGs (n=3). Significance was determined by two-tailed unpaired Student's t test (A, B) and indicated as the P value. **** $P < 0.0001$; n.s., no significant difference. Data are presented as mean ± s.d. (A, B).

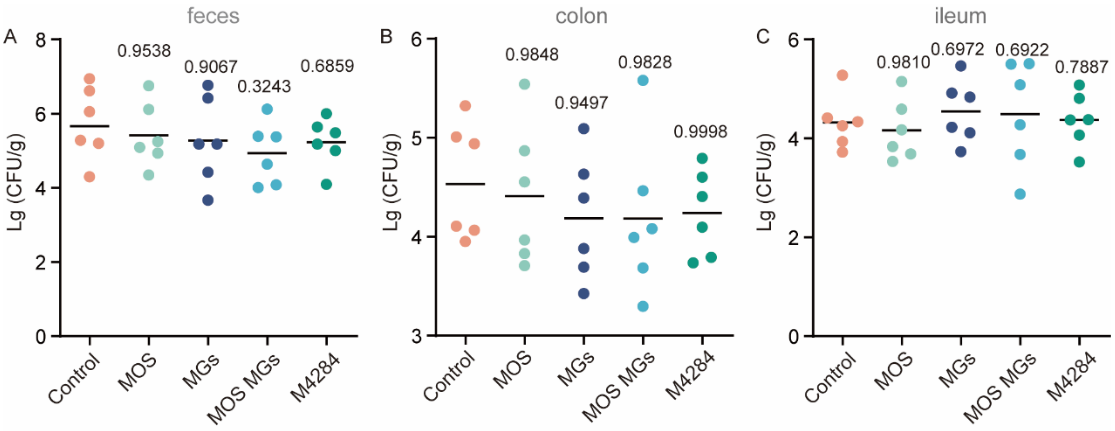

Supplementary Figure 8

A–C) Colonization levels of Δ*fimH* in feces (A), colon (B), and ileum (C) at 48 hours post-treatment with MOS, MGs, MOS MGs, and M4284 (n=6). Significance was determined by a two-sided Mann–Whitney U test, and indicated as the P value; n.s., not significant;. Data are presented as mean ± s.d.

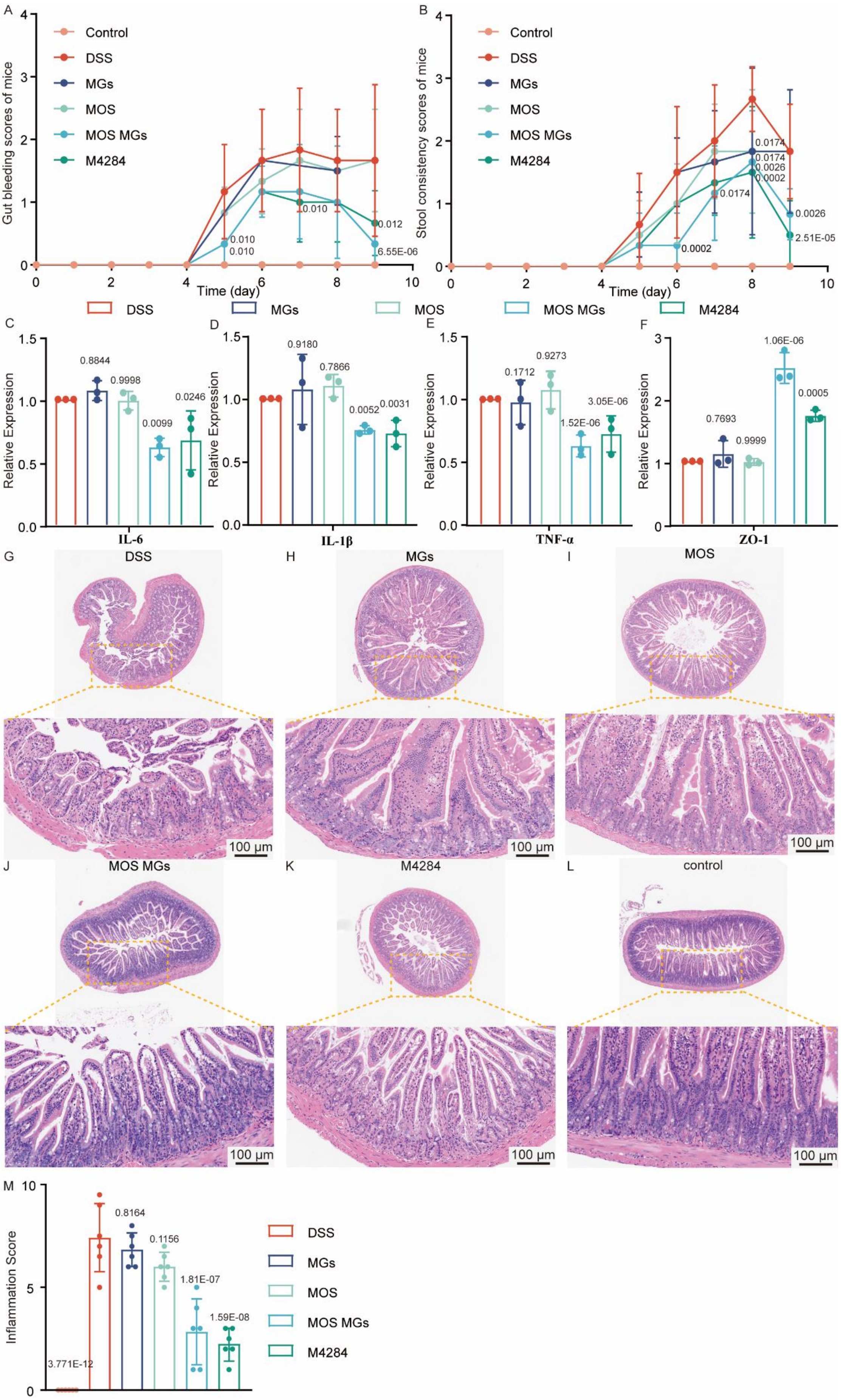

A
B
Control
DSS
MGs
MOS
MOS MGs
M4284
Gut bleeding scores of mice
Stool consistency scores of mice
Time (day)
0.010
0.010
0.010
0.012
6.55E-06
0.0174
0.0174
0.0026
0.0002
0.0174
0.0002
0.0026
2.51E-05
C
D
E
F
Relative Expression
IL-6
IL-1β
TNF-α
ZO-1
0.8844
0.9998
0.0099
0.0246
0.9180
0.7866
0.0052
0.0031
0.1712
0.9273
1.52E-06
3.05E-06
1.06E-06
0.7693
0.9999
0.0005
G
DSS
H
MGs
I
MOS
J
MOS MGs
K
M4284
L
control
100 μm
M
Inflammation Score
3.771E-12
0.8164
0.1156
1.81E-07
1.59E-08

Supplementary Figure 9

A-B) Gut bleeding scores (A) and stool consistency scores (B) were recorded in detail and analyzed (n=6). C-F) mRNA expression levels of IL-6, IL-1β, TNF-α, and ZO-1 in the ileum (n=3). G-L) Representative H&E staining images of ileum tissue of each group. M) Inflammation scores according to H&E staining were analyzed in each group (n=6). Significance was determined by two-way ANOVA (A, B), one-way ANOVA (C-F), and indicated as the P value; n.s., not significant; * $p < 0.05$, ** $p < 0.01$, *** $p < 0.001$, **** $p < 0.0001$. Data are presented as mean ± s.d..

Supplementary Table 1. Strains and plasmids used in this study

Strains	Genotype or description	Source
LF82	Adherent–Invasive *E. coli* LF82 O83:H7 strain	Lab collection
BL21	Recombinant protein expression strain	Lab collection
DH5α	*E. coli* DH5α/λpir strain	Lab collection
Δ*fimH*	*fimH* deletion mutant in LF82	This work
BL21- *fimH*	BL21(DE3) containing pET28a- *fimH*	This work
**Plasmids**		
pKD3	Carries the chloramphenicol resistance gene; Cm^R	This work
pUC57-Tac-mCherry	Carries the *mCherry* fragment under the Tac promoter; Amp^R	Lab collection
pET28a	T7 bacteria expression vector, Km^R	Lab collection
pET28a- *fimH*	pET28a carrying the *fimH* fragment，Km^R	Lab collection

Supplementary Table 2. Primers used in this study (5'-3').

Primers for gene mutation		
fimH	F	TGATTAGCATCACCTATACCTACAGCTGAACCCGAAGAGA TGATTGTAGTGTAGGCTGGAGCTGCTTCG
fimH	R	GCTTCAGGTAATATTGCGTACCTGCATTAGCAATGCCCTG TGATTTCTCATATGAATATCCTCCTTAG
Primers for gene mutation identifying		
fimH	F	CTAATTGTCCGGTGGGAACGTCGA
fimH	R	CCGAAGTCCCTACTGCTCCTAACG
Primers for pET28a identifying		
pET-28a	F	TAATACGACTCACTATAGGG
pET-28a	R	GCTAGTTATTGCTCAGCGG
Primers for protein purification		
FimH	F	CGCGGATCCATGAAACGAGTTATTACCCTGT
FimH	R	CCCAAGCTTTTATTGATAAACAAAAGTCACG
Primers for qRT-PCR		
GAPDH	F	ATGGCCTTCCGTGTTCCTAC
GAPDH	R	CAGATGCCTGCTTCACCAC
IL-1β	F	ATGGCAACTGTTCCTGAACTCAACT
IL-1β	R	CAGGACAGGTATAGATTCTTTCCTTT
IL-6	F	CTAGGTTTGCCGAGTAGATCT
IL-6	R	CACAAAGCCAGAGTCCTTCAGAGA
ZO-1	F	CAACATACAGTGACGCTTCACA
ZO-1	R	CACTATTGACGTTTCCCCACTC
TNF-α	F	GAGGCCAAGCCCTGGTATG
TNF-α	R	CGGGCCGATTGATCTCAGC

Supplementary Table 3. Abbreviations in this study.

Abbreviations	
AIEC	Adherent–Invasive *E. coli* LF82 O83:H7 strain
WT	Wild type LF82 bacteria
IBD	Inflammatory bowel disease
MOS	Mannan oligosaccharides
GelMA	Gelatin methacryloyl
MGs	GelMA microgels
MOS MGs	GelMA microgels containing MOS.
CFU	Colony-forming unit
MOI	Multiplicity of infection
FITC	Fluorescein isothiocyanate isomer I
PBS	Phosphate buffered saline
DSS	Dextran sulfate sodium salt
DAI	Disease activity index
IVIS	In Vivo Imaging Systems
FimH	The adhesion portion of type 1 fimbriae in *Escherichia coli*[1]
ATPS	Aqueous two-phase system
PEO	Polyethylene oxide
LAP	Lithium Phenyl-2,4,6-trimethylbenzoylphosphinate
SGF	Simulated gastric fluid
SIF	Simulated intestinal fluid
HUVECs	human umbilical vein endothelial cells
PCoA	Principal coordinate analysis
PLS-DA	Partial least squares discriminant analysis
OTU	Operational taxonomic units
LEfSe	Linear discriminant analysis effect size
LDA	Linear discriminant analysis
SPR	Surface plasmon resonance
H&E	Hematoxylin and eosin
IL-6	Interleukin-6

**Abbreviations**	
IL-1β	Interleukin-1 beta
TNF-α	Tumor necrosis factor alpha
ZO-1	Zona occludens-1

1 Zhang, W. *et al.* *Escherichia coli* adhesion portion FimH functions as an adjuvant for cancer immunotherapy. *Nat Commun* **11**, 1187, doi:10.1038/s41467-020-15030-4 (2020).
