# Supplementary material for: Fishing out AIEC with FimH capturing microgels for inflammatory bowel disease treatment
Source: Nat Commun. 2025 Aug 25;16:7924. doi: 10.1038/s41467-025-63276-7 (PMC12378211; doi:10.1038/s41467-025-63276-7)
Supplement: Supplementary file 2 — Reporting Summary [file 41467_2025_63276_MOESM2_ESM.pdf]

nature portfolio

Corresponding author(s): Guosheng Tang

Last updated by author(s): Aug 1, 2025

Reporting Summary

Nature Portfolio wishes to improve the reproducibility of the work that we publish. This form provides structure for consistency and transparency in reporting. For further information on Nature Portfolio policies, see our Editorial Policies and the Editorial Policy Checklist.

Statistics

For all statistical analyses, confirm that the following items are present in the figure legend, table legend, main text, or Methods section.

n/a	Confirmed	
☐	☒	The exact sample size (n) for each experimental group/condition, given as a discrete number and unit of measurement
☐	☒	A statement on whether measurements were taken from distinct samples or whether the same sample was measured repeatedly
☐	☒	The statistical test(s) used AND whether they are one- or two-sided<br>*Only common tests should be described solely by name; describe more complex techniques in the Methods section.*
☒	☐	A description of all covariates tested
☐	☒	A description of any assumptions or corrections, such as tests of normality and adjustment for multiple comparisons
☐	☒	A full description of the statistical parameters including central tendency (e.g. means) or other basic estimates (e.g. regression coefficient) AND variation (e.g. standard deviation) or associated estimates of uncertainty (e.g. confidence intervals)
☐	☒	For null hypothesis testing, the test statistic (e.g. F, t, r) with confidence intervals, effect sizes, degrees of freedom and P value noted<br>*Give P values as exact values whenever suitable.*
☒	☐	For Bayesian analysis, information on the choice of priors and Markov chain Monte Carlo settings
☐	☒	For hierarchical and complex designs, identification of the appropriate level for tests and full reporting of outcomes
☒	☐	Estimates of effect sizes (e.g. Cohen's d, Pearson's r), indicating how they were calculated

Our web collection on statistics for biologists contains articles on many of the points above.

Software and code

Policy information about availability of computer code

Data collection	Sequencing data was collected by Illumina HiSeq 2500 (Illumina); SPR data was collected by Biacore X100.
Data analysis	MedCalc (v12.3.0.0); Microsoft Excel 2019 (16.0.14026.20294); GraphPad Prism(10.0.5); ImageJ (v1.8.0);QIIME package (version 1.8.0);VSEARCH (version 2.4.2);Biacore X100 evaluation software.

For manuscripts utilizing custom algorithms or software that are central to the research but not yet described in published literature, software must be made available to editors and reviewers. We strongly encourage code deposition in a community repository (e.g. GitHub). See the Nature Portfolio guidelines for submitting code & software for further information.

Data

Policy information about availability of data

All manuscripts must include a data availability statement. This statement should provide the following information, where applicable:

- Accession codes, unique identifiers, or web links for publicly available datasets
- A description of any restrictions on data availability
- For clinical datasets or third party data, please ensure that the statement adheres to our policy

16S rRNA gene sequencing data have been deposited in the NCBI SRA database under accession code PRJNA1182980.

Research involving human participants, their data, or biological material

Policy information about studies with human participants or human data. See also policy information about sex, gender (identity/presentation), and sexual orientation and race, ethnicity and racism.

Reporting on sex and gender	not applicable
Reporting on race, ethnicity, or other socially relevant groupings	not applicable
Population characteristics	not applicable
Recruitment	not applicable
Ethics oversight	not applicable

Note that full information on the approval of the study protocol must also be provided in the manuscript.

Field-specific reporting

Please select the one below that is the best fit for your research. If you are not sure, read the appropriate sections before making your selection.

[x] Life sciences [ ] Behavioural & social sciences [ ] Ecological, evolutionary & environmental sciences

For a reference copy of the document with all sections, see nature.com/documents/nr-reporting-summary-flat.pdf

Life sciences study design

All studies must disclose on these points even when the disclosure is negative.

Sample size	Sample sizes were determined empirically and according to extensive literature on the IBD infection models, and appropriate statistical analyses were performed. Mice colonization experiments were conducted twice with at least 3 mice ($n \geq 3$) in each group, and the combined data for the two experiments was used for statistical analysis. For 16S rRNA gene sequencing, total DNA was extracted from 6 different biological samples of mouse feces before and after treatment. All other in vitro experiments were repeated at least three times ($n \geq 3$). For immunostaining experiments, three visual fields from each mouse were analyzed, and six mice were examined.
Data exclusions	No data were excluded from the analyses.
Replication	All the reported experiments were reproducible. Data reproducibility was confirmed by three independent experiments.
Randomization	All samples were assigned to groups randomly. All mice were randomized to avoid cage effects. Other data collection was not randomized, but always done in parallel with controls.
Blinding	The study employed a double-blind design, where both the experimenters and the mice were blinded to the treatment allocation. Treatment allocations were concealed in coded containers, and the animals were randomly assigned to treatment groups by an independent researcher who was not involved in the treatment or data analysis. Data analysis was also performed by researchers who were blinded to the group allocation.

Reporting for specific materials, systems and methods

We require information from authors about some types of materials, experimental systems and methods used in many studies. Here, indicate whether each material, system or method listed is relevant to your study. If you are not sure if a list item applies to your research, read the appropriate section before selecting a response.

Materials & experimental systems

n/a	Involved in the study	
[x]	[ ]	Antibodies
[ ]	[x]	Eukaryotic cell lines
[x]	[ ]	Palaeontology and archaeology
[ ]	[x]	Animals and other organisms
[x]	[ ]	Clinical data
[x]	[ ]	Dual use research of concern
[x]	[ ]	Plants

Methods

n/a	Involved in the study	
[x]	[ ]	ChIP-seq
[x]	[ ]	Flow cytometry
[x]	[ ]	MRI-based neuroimaging

Eukaryotic cell lines

Policy information about cell lines and Sex and Gender in Research

Cell line source(s)	ATCC
Authentication	HeLa cells and Caco-2 cells were authenticated by STR (short tandem repeat) profiling.
Mycoplasma contamination	Mycoplasma testing was not performed.
Commonly misidentified lines (See ICLAC register)	Not applicable.

Animals and other research organisms

Policy information about studies involving animals; ARRIVE guidelines recommended for reporting animal research, and Sex and Gender in Research

Laboratory animals	Seven to eight-week-old specific pathogen-free (SPF) female C57BL/6 J mice
Wild animals	This study did not involve wild animals.
Reporting on sex	Female
Field-collected samples	This study did not involve samples collected from the field.
Ethics oversight	All animal studies were conducted according to protocols approved by the Institutional Animal Care Committee of Nankai University (Tianjin, China) and performed under protocol no. IACUC 2016030502.

Note that full information on the approval of the study protocol must also be provided in the manuscript.

Plants

Seed stocks	This study did not involve plants.
Novel plant genotypes	not applicable
Authentication	not applicable
