# Supplementary material for: Fishing out AIEC with FimH capturing microgels for inflammatory bowel disease treatment
Source: Nat Commun. 2025 Aug 25;16:7924. doi: 10.1038/s41467-025-63276-7 (PMC12378211; doi:10.1038/s41467-025-63276-7)
Supplement: Supplementary file 3 — Transparent Peer Review file [file 41467_2025_63276_MOESM3_ESM.pdf]

nature portfolio

Peer Review File

Fishing Out AIEC with FimH Capturing Microgels for Inflammatory Bowel Disease Treatment

Corresponding Author: Professor Guosheng Tang

This file contains all reviewer reports in order by version, followed by all author rebuttals in order by version.

Version 0:

Reviewer comments:

Reviewer #1

(Remarks to the Author)
Dear Editor,
The manuscript by Wu et al entitled “Fishing for AIEC: Using microgels to targeted attract FimH for combating IBD” presents an innovative approach to addressing inflammatory bowel disease (IBD). The study introduces a novel strategy to trap adherent-invasive Escherichia coli (AIEC) bacteria and remove them from the intestinal mucosa of patients with Crohn’s disease. The authors have developed specialized microgels loaded with mannan oligosaccharides to enable competitive absorption of AIEC, both in vitro and in vivo.
It is important to note that, as a reviewer, I am not an expert in the synthesis of microgel structures and therefore cannot critically assess the sections of the paper pertaining to their fabrication or the novelty of this approach. My review will primarily focus on evaluating the study's investigation into the effectiveness of the microgels in trapping AIEC.

Major Comments
1. Title: The title contains grammatical errors and lacks clarity. I recommend reformulating it in correct English to improve its readability and precision.
2. Line 286: The authors state that "MOS GelMA MGs adsorb AIEC and reduce its colonization in the small intestine." However, this claim is not adequately demonstrated in the paper. The only figure addressing AIEC colonization is Figure S7, which merely shows the absence of effects in the ΔfimH AIEC strain. To substantiate their claim, the authors should present data on the colonization levels of the WT strain in stool, as well as its association with the ileum and colon, in mice treated or untreated with MOS GelMA MGs. This data would be critical to support the assertion that the microgel effectively reduces AIEC colonization and not only decrease intestinal inflammation.
3. Did the authors examine the inflammatory response in the small intestine, as they did for the colonic mucosa? Including this analysis would provide a more comprehensive understanding of the microgel's effects.
4. Figure 6 G-J: The authors performed RT-qPCR to quantify inflammatory gene expression, using Trizol for RNA extraction. It is well-documented that DSS significantly inhibits polymerase activity, and specific RNA purification methods should be employed to extract RNA from DSS-treated tissues (see PMID: 24010775). As such, I have reservations about the validity of the results presented in this figure.
5. The authors report an increase in ZO-1 expression following microgel administration. Was this associated with an improvement in the intestinal epithelial barrier function? Measuring intestinal permeability using Dextran-FITC could provide valuable insight into this question.
6. Did the authors investigate the microbiota composition in stool or associated to the mucosa? Please clarify this point in the Results section and provide details in the Materials and Methods section. The analysis of mucosa-associated microbiota would be much more relevant to the study.

Minor Comments
1. Line 73: The authors state that there is a "68.7% increase in total death associated with IBD." As IBD is generally not considered a fatal disease, this statement appears inaccurate. Furthermore, the referenced source (Catch-bond mechanism of the bacterial adhesin FimH) does not support this claim. The reference should be replaced with a more appropriate one.
2. Line 95: References 1 and 16 are not suitable for this context. Instead, the authors should cite PMID: 23358328 and PMID: 17525800, which are more relevant to the sentence.
3. Line 123: The phrase "ATPS is formed without oil-free and surfactant-free conditions" is unclear. It is not evident whether oil and surfactants are used in the formation of ATPS. This should be corrected for clarity and grammatical accuracy.

4. Line 160: The authors claim that "the adsorption of AIEC decreases their abundance without impacting beneficial probiotics." The term probiotics is not appropriate in this context. Please consider revising it to "beneficial microbes" or "beneficial bacteria."
5. Line 169: The manuscript is difficult to follow due to the excessive use of abbreviations. A comprehensive list of abbreviations should be included, and all abbreviations should be spelled out in full the first time they appear (e.g., MOS GelMA MGs on line 169 and in all figure legends).
6. Line 183: Please clarify the meanings of LAP and PEO, as they are not defined. Similarly, define SGF and SIF on line 247.
7. Line 221: Specify which cell types were used in the experiments.
8. Line 266: The term WT is ambiguous. Does this refer to WT LF82 bacteria? The same clarification is needed for line 281.
9. Line 280: Specify the animal model used in the study.
10. Line 298: The phrase "very low DAI" seems contradictory to the context. Did the authors mean "very high DAI"? Please revise.
11. Line 340: The statement "GelMa MG restores dysbiosis" should be replaced with "GelMa MG prevents dysbiosis" or "GelMa MG favors eubiosis."
12. Line 367: The conclusion that "Bifidobacteriaceae were the key type of bacteria contributing to gut microbiota recovery" is unclear. Please elaborate on how this conclusion was reached based on the data presented.
13. Discussion Section: The discussion is underdeveloped and reads more like a conclusion. It should be expanded to compare the authors' findings with relevant studies in the literature that used strategies to clear AIEC bacteria. The authors should also discuss how their approach is advantageous or complementary to existing methods.
14. Line 471: The authors describe the strategy for overexpressing and purifying FimH but it is not clear in which figure this approach is presented. Please ensure this is clearly indicated, and revise the figure legends to include more methodological details to help readers understand what is shown in the panels.
15. Line 537: Clarify whether the microgels were administered to mice via drinking water or oral gavage. If the latter, specify the volume administered to each mouse.

Overall, this manuscript presents findings of interest to the field of IBD research. However, additional data are required to substantiate the authors' conclusions, and several key points need further clarification.

Reviewer #2

(Remarks to the Author)
The manuscript entitled "Fishing for AIEC: Using Microgels to Targeted Attract FimH for Combating IBD" proposed the concept of competitive adsorption by leveraging the binding properties of FimH with mannose, prompting microgels competitively absorbing AIEC over intestinal epithelial cells for the treatment of IBD.
There are a lot of questions worth considering and addressing, so major revision should be conducted before further consideration. The details were as follows:
1. The role of Fig. 1 is very critical, but it still needs to be beautified and visualized by the authors.
2. The full name of AIEC does not appear in the abstract and introduction.
3. In addition to AIEC, FimH is also present on the surface of other Enterobacteriaceae bacteria, including but not limited to Enterobacter, Klebsiella, Salmonella, Shigella, etc. The author need to discuss about this.
4. The introduction section is too long. The author provides too much information on the background and synthesis of the material. The important role of the introduction is to effectively guide the readers into the theme of the paper and help the readers understand the importance and necessity of the research. Therefore, only a brief introduction to the design and synthesis methods is needed.
5. The name of the material "MOS Gelma MGs" is too long and can be appropriately abbreviated.
6. Animal experiment require appropriate positive control groups to judge the actual efficacy of MOS Gelma MGs, which includes treatment with fimH inhibitors or first-line drug 5-ASA.
7. Why was the biosafety experiment conducted using HeLa cells? It should be conducted on intestinal epithelial cell line.
8. According to the scale bar in Figure 4D, the diameter of the microgel is 100 micrometers, but in Figure 2, the diameter of the microgel is 500 micrometers, which is very confusing.
9. The author needs to provide more evidence to prove the competitive adsorption of the microgel on AIEC. In vitro experiments should co-incubate AIEC, intestinal epithelial cells, and MOS Gelma MGs, and then analyze the proportion of AIEC adhering to intestinal epithelial cells and MOS Gelma MGs.
10. In Figure 4G, how did the author detect the colonization of AIEC in the intestinal epithelium? Was the sample feces or intestinal mucosal tissue? The research method for this data was not written in the methods section.
11. In the animal model, oral gavage of a large amount of AIEC (2×10^9/mouse/day) daily was planned. Is the gavage amount of MOS Gelma MGs sufficient to adsorb all the AIEC?
12. Has the author ever detected the baseline abundance of AIEC in the intestine, as the survival rate of AIEC is theoretically significantly reduced after passing through gastric fluid?

Reviewer #3

(Remarks to the Author)
This manuscript by Wu et al designed the porous microgels in order to 'fishing for AIEC' and investigated their advantages and mechanism in inflammatory bowel disease treatment. However, competitively selective bacterial capture is not new,

which have been reported in many other papers (ACS Appl. Mater. Interfaces 2022, 14, 42, 47323–47344). More importantly, this paper requires more solid evidence and improvement to show the fate of microgels after absorbing AIEC. In my opinion, this paper is not suitable for publication in Nature Communications. Please refer to the detailed comments below:

1. The porous microgels were fabricated via an aqueous two-phase system. Please give more evidence to confirm its advantages. And the size and distribution of the microgels' porosity should be estimated.

2. The mechanism of interaction between FimH and mannan oligosaccharides has been reported in the previous work. What's the promotion of microgels in this work?

3. In the in vitro co-culture system of microgels and AIEC, what effects does the adsorption of AIEC onto microgels have on their physicochemical properties?

4. In the in vivo experiment, the fate of microgels that have already absorbed AIEC was important but not investigated. After scavenging the AIEC, the alteration of the microgel complex and how it was drawn out were not fully understood.

5. The microgels contribute to the recovery via the competitive interaction with AIEC when compared with epithelial glycoproteins. Consequently, in vitro experiments probing such competitive interactions, such as co-culturing microgels with AIEC in the presence of colonic epithelial, should be conducted.

6. In this work, the authors only did the experiment in ulcerative colitis model but IBD includes both ulcerative colitis and Crohn's disease. So, the claim that the strategy for managing IBD is not accurate enough.

Version 1:

Reviewer comments:

Reviewer #1

(Remarks to the Author)
The authors have adequately addressed my previous comments and revised the manuscript accordingly. I have only a few minor suggestions:
Figures 4G–I and S8: Please indicate in the figure legend the specific time point post-infection at which the samples were analyzed.

The figure legends state that statistical analyses were performed using a t-test. However, when comparing multiple conditions, a one-way ANOVA should be used. Additionally, for all figures comparing more than two experimental conditions, ANOVA should be used. Please indicate which groups were compared by adding connecting lines to the graphs.

I do not have access to the supplemental tables. If not already included, please provide a table listing the sequences of the primers used for RT-qPCR.

Reviewer #2

(Remarks to the Author)
The authors have carefully considered the reviewers' comments in the revised version of the manuscript, adding new experimental data and references to support their conclusions and improving the methods and discussion sections. Therefore, I recommend publishing the manuscript in Nature Communications.

Reviewer #3

(Remarks to the Author)
I have reviewed the authors' responses and the revisions made to the manuscript. I have no further questions with the technical and experimental aspects of the paper. However, I remain unconvinced about the novelty of the work. The fabrication method and materials used to prepare the porous microgels lack novelty. Crucially, the key design of competitively selective bacterial capture has been previously reported. The only significant contribution appears to be the in vivo therapeutic study, which is insufficient to warrant publication in a high-impact journal of Nature Communications.

Open Access This Peer Review File is licensed under a Creative Commons Attribution 4.0 International License, which permits use, sharing, adaptation, distribution and reproduction in any medium or format, as long as you give appropriate credit to the original author(s) and the source, provide a link to the Creative Commons license, and indicate if changes were made.
In cases where reviewers are anonymous, credit should be given to 'Anonymous Referee' and the source.
The images or other third party material in this Peer Review File are included in the article's Creative Commons license, unless indicated otherwise in a credit line to the material. If material is not included in the article's Creative Commons license and your intended use is not permitted by statutory regulation or exceeds the permitted use, you will need to obtain permission directly from the copyright holder.
To view a copy of this license, visit https://creativecommons.org/licenses/by/4.0/

Response to Referees

Reviewer Comments:

Reviewer #1 (Remarks to the Author):

Dear Editor,

The manuscript by Wu et al entitled "Fishing for AIEC: Using microgels to targeted attract FimH for combating IBD" presents an innovative approach to addressing inflammatory bowel disease (IBD). The study introduces a novel strategy to trap adherent-invasive Escherichia coli (AIEC) bacteria and remove them from the intestinal mucosa of patients with Crohn's disease. The authors have developed specialized microgels loaded with mannan oligosaccharides to enable competitive absorption of AIEC, both in vitro and in vivo.

It is important to note that, as a reviewer, I am not an expert in the synthesis of microgel structures and therefore cannot critically assess the sections of the paper pertaining to their fabrication or the novelty of this approach. My review will primarily focus on evaluating the study's investigation into the effectiveness of the microgels in trapping AIEC.

Response:

We thank the reviewer for the patient and thoughtful reading as well as the constructive comments and advice about our manuscript. We have revised the manuscript based on your comments and suggestions.

Major Comments

1. Title: The title contains grammatical errors and lacks clarity. I recommend reformulating it in correct English to improve its readability and precision.

Response:

We thank the reviewer for the valuable suggestion. We have now polished the title.

Fishing Out AIEC with FimH-Capturing Microgels: Towards IBD Treatment

2. Line 286: The authors state that "MOS GelMA MGs adsorb AIEC and reduce its colonization in the small intestine." However, this claim is not adequately demonstrated in the paper. The only figure addressing AIEC colonization is Figure S7, which merely shows the absence of effects in the ΔfimH AIEC strain. To substantiate their claim, the authors should present data on the colonization levels of the WT strain in stool, as well as its association with the ileum and colon, in mice treated or untreated with MOS GelMA MGs. This data would be critical to support the assertion that the microgel effectively reduces AIEC colonization and not only decrease intestinal inflammation.

Response:

We would like to thank the reviewer for the valuable comment. We have now supplemented data on the colonization levels of the WT and Δ*fimH* strain in stool, as well as its association with the ileum and colon. Additionally, we have compared the colonization levels between mice treated and untreated with MOS MGs. The results demonstrated a significant reduction in bacterial colonization in the feces, colon, and ileum of the treatment group, supporting the effectiveness of the microgels in reducing AIEC colonization, rather than merely alleviating intestinal inflammation. The relative information has been added in the revised manuscript. (lines 266-282 and Extended Data Fig. 4G-I and Fig. S8 of the revised manuscript). Relevant data have also been included in this response below for your reference.

Related content

*We further conducted in vivo experiments for verification using a DSS-induced mouse model of colitis. We established five experimental groups: a control group (administered AIEC by gavage following antibiotic treatment), a MOS treatment group, a MGs treatment group, an MOS MGs treatment group, and a positive control group (M4284[19], a high-affinity inhibitory mannoside). Following treatment, fecal samples, colon, and ileum tissues were collected for bacterial enumeration using plate counting (**Fig. 4G-I**). No significant differences in bacterial colonization were observed in the MOS or MGs treatment groups compared to the control. In contrast, treatment with M4284 led to a significant reduction of AIEC colonization in the ileum and colon, but not in the feces. Notably, mice treated with MOS MGs exhibited a significant decrease*

*in AIEC levels in the ileum, colon, and feces. These results indicate that MOS MGs, beyond mimicking the anti-adhesive function of M4284, also exert an active bacterial-trapping effect, thereby promoting the effective clearance of AIEC from the intestinal tract. However, when ΔfimH was administered orally, there were no significant differences between the five groups (**Fig. S8**). The results indicated that in vivo, MOS MGs adsorb AIEC by binding to FimH and reduce its colonization in the small intestine.*

Fig 4G-I

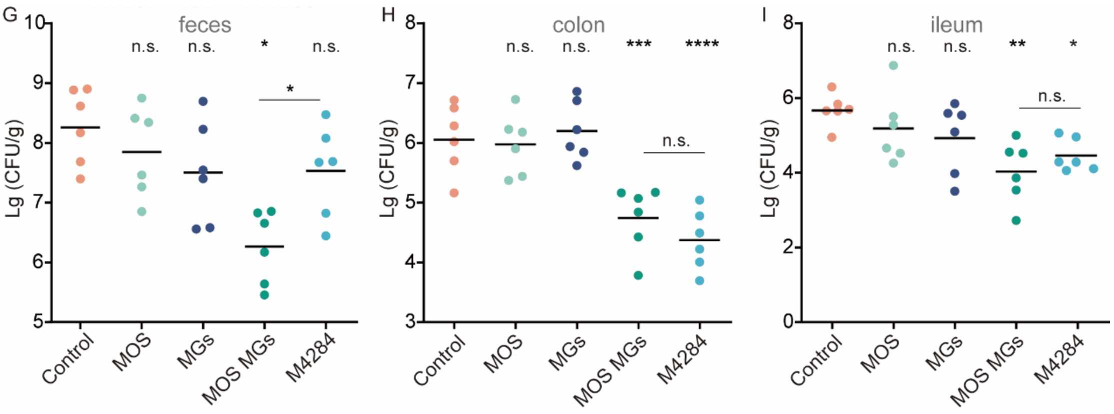

G–I) Colonization levels of WT in feces (G), colon (H), and ileum (I) after treatment with MOS, MGs, MOS MGs, and M4284 (n=6).

Fig S8

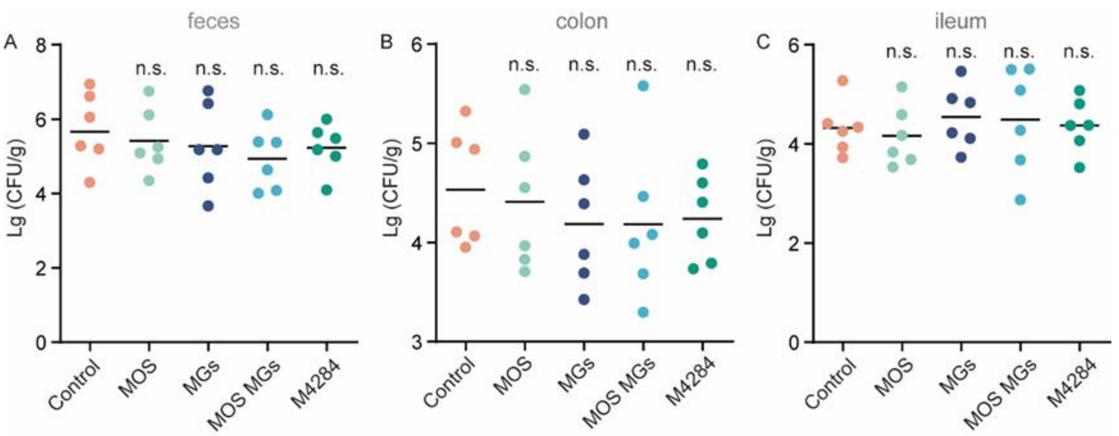

A–C) Colonization levels of ΔfimH in feces (A), colon (B), and ileum (C) after treatment with MOS, MGs, MOS MGs, and M4284 (n=6).

3. Did the authors examine the inflammatory response in the small intestine, as they did for the colonic mucosa? Including this analysis would provide a more comprehensive understanding of the microgel's effects.

Response:

We thank the reviewer for the valuable suggestion. We have now supplemented the analysis by examining the inflammatory response in ileum, as we did for the colonic mucosa. The results showed that treatment with MOS MGs led to a reduction in the gene expression levels of inflammatory cytokines, while the expression of ZO-1 was increased. This trend was consistent with observations in the colonic mucosa, indicating that MOS MGs treatment helps alleviate inflammation and promotes intestinal barrier repair. The relevant information has been added to the revised manuscript. (lines 331 and Extended Data Fig.S9 C-F of the revised manuscript). Relevant data have also been included in this response below for your reference.

Related content

*RT-PCR analyses of IL-6, IL-1β, TNF-α and ZO-1 expression (**Fig. S9C-F**) were performed. The results showed trends consistent with those observed in the colon.*

Fig S9 C-F

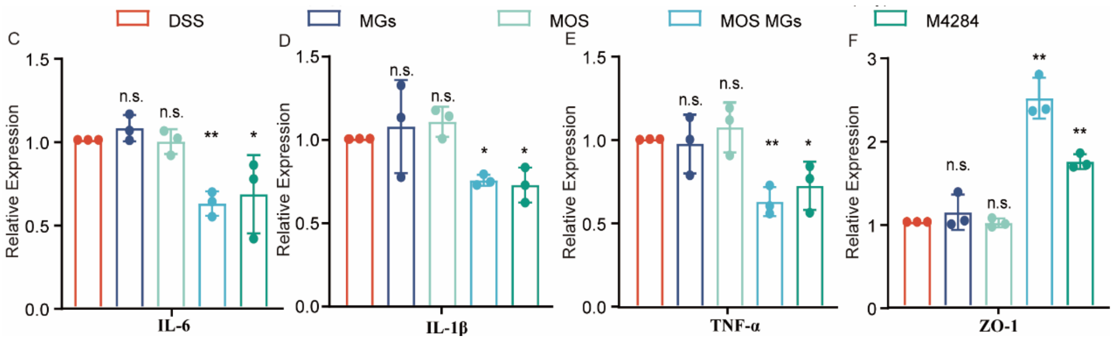

C-F) mRNA expression levels of IL-6, IL-1β, TNF-α, and ZO-1 in the ileum (n=3).

4. Figure 6 G-J: The authors performed RT-qPCR to quantify inflammatory gene expression, using Trizol for RNA extraction. It is well-documented that DSS significantly inhibits polymerase activity, and specific RNA purification methods should be employed to extract RNA from DSS-treated tissues (see PMID: 24010775). As such, I have reservations about the validity of the results presented in this figure.

Response:

We thank the reviewer for the valuable comment. Following your recommendation and the article (PMID: 24010775), we have modified the RNA extraction method to account for the potential inhibition of polymerase activity by DSS. We have re-performed the RT-qPCR experiments using the updated protocol. The results showed that, after treatment with MOS MGs, the expression levels of inflammatory cytokine genes were significantly reduced, while the expression of tight junction proteins was markedly increased, consistent with the original trend. The relevant information has been added to the revised manuscript. (lines 312-314; 322-325; 329; 622-624 and Fig.6I-L, Fig. S9 C-F of the revised manuscript). Relevant data have also been included in this response below for your reference.

Related content

lines 315-317

*Furthermore, mice treated with MOS MGs exhibited a significant upregulation of ZO-1 gene expression, indicating that MOS MGs effectively enhance intestinal epithelial barrier function (**Fig. 6I**).*

lines 325-327

Treatment with M4284 or MOS MGs significantly downregulated pro-inflammatory genes (IL-6, IL-1β, TNF-α) compared to the DSS group, with MOS MGs showing the most pronounced effect (Fig. 6J-L).

lines 331-332

*RT-PCR analyses of IL-6, IL-1β, TNF-α and ZO-1 expression (**Fig. S9C-F**) were performed.*

lines 655-657

RNA isolation and RT-PCR

To detect gene expression in vivo, collect mouse ileum and colon samples and gently scrape off contents. RNA was purified via precipitation with lithium chloride[54]

Fig 6 I-L

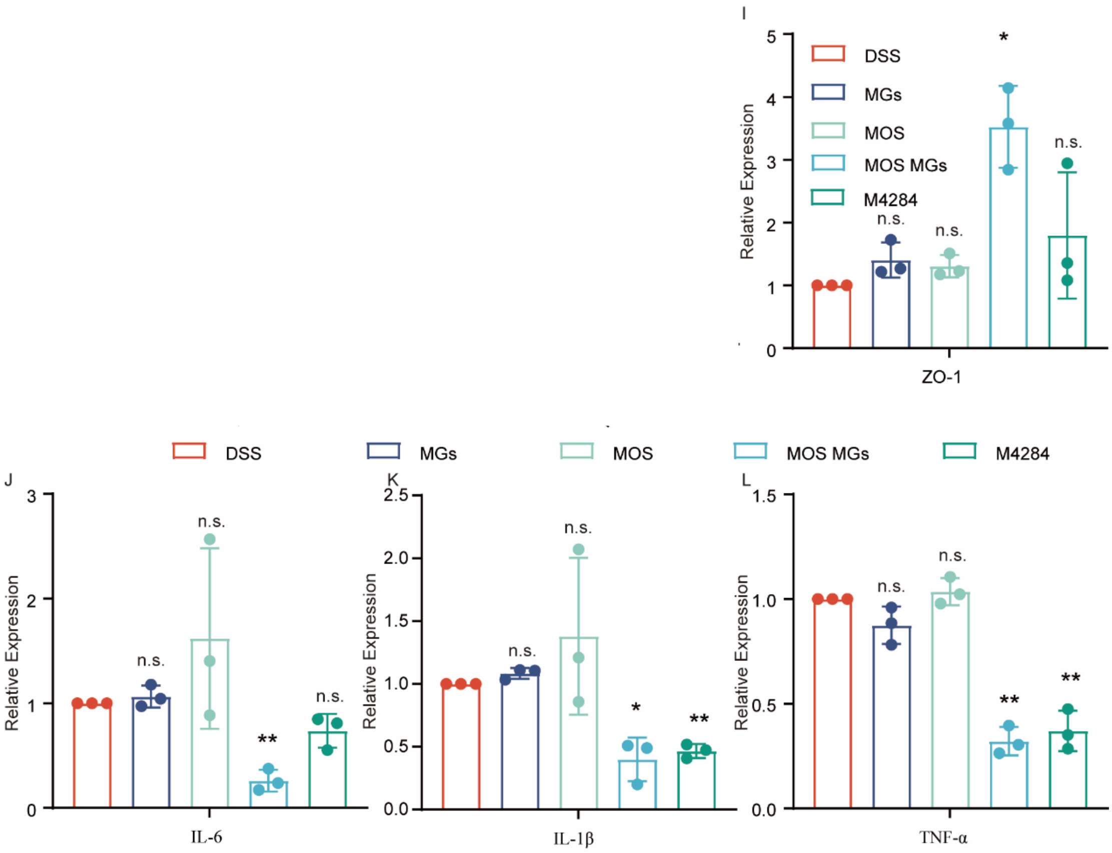

I-L) Colonic mRNA levels of ZO-1, IL-6, IL-1β, and TNF-α, (n=3).

Fig S9 C-F

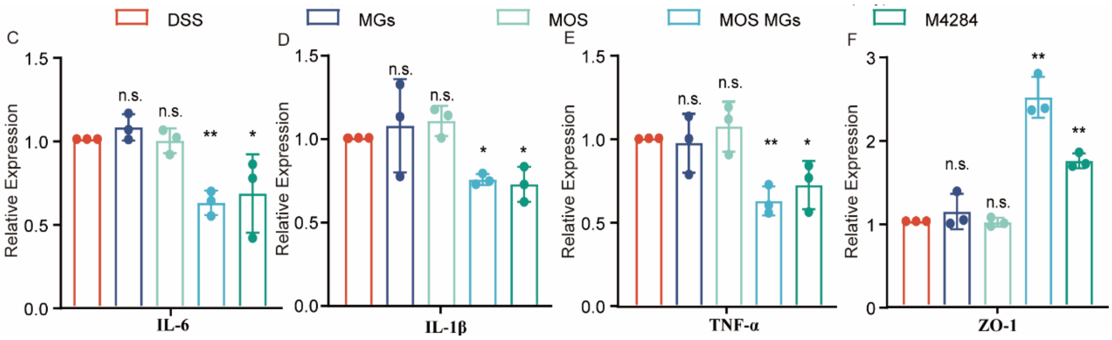

C-F) mRNA expression levels of IL-6, IL-1β, TNF-α, and ZO-1 in the ileum (n=3).

5. The authors report an increase in ZO-1 expression following microgel administration. Was this associated with an improvement in the intestinal epithelial barrier function? Measuring intestinal permeability using Dextran-FITC could provide valuable insight into this question.

Response:

We thank the reviewer for the valuable comment. Following your recommendation, we have supplemented the manuscript with data measuring intestinal permeability using Dextran-FITC. The concentration of Dextran-FITC in the blood was significantly reduced in the MOS MGs-treated group, indicating that MOS MGs treatment contributed to the restoration of the intestinal permeability. The relevant information has been added to the revised manuscript. (lines 306-315 and Fig. 6H of the revised manuscript). Relevant data have also been included in this response below for your reference.

Related content

*First, we evaluated whether MOS MGs could improve intestinal epithelial barrier function. To this end, we assessed intestinal permeability using Dextran-FITC and examined the expression of ZO-1. ZO-1 is a tight junction protein that forms tight connections between epithelial cells in the intestine, thereby regulating the permeability of the intercellular space. In patients with IBD, ZO-1 expression and function are often compromised, leading to disruption of the intestinal mucosal barrier and increased permeability to bacteria and toxins[25]. Compared to the DSS group, treatment with MGs led to a certain reduction in intestinal permeability. However, both the MOS MGs and M4284 treatment groups showed a marked decrease in permeability (**Fig. 6H**).*

Fig 6 H

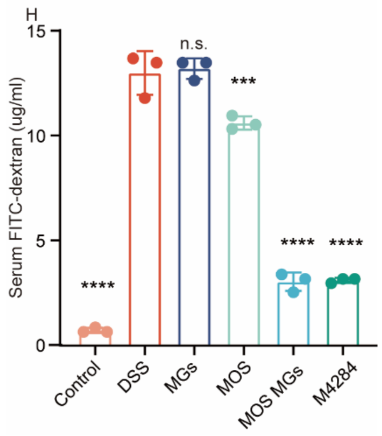

H) Intestinal permeability assessment using FITC-dextran (n=3).

6. Did the authors investigate the microbiota composition in stool or associated to the mucosa? Please clarify this point in the Results section and provide details in the Materials and Methods section. The analysis of mucosa-associated microbiota would be much more relevant to the study.

Response:

We thank the reviewer for the valuable comment. In the original manuscript, we indeed collected stool samples to analyze the microbiota composition. This information has now been clarified in the revised Materials and Methods section (lines 635-636), and in the Results section (lines 337-339).

Regarding the relevance of mucosa-associated microbiota, we acknowledge that the analysis of the microbiota directly associated with the mucosa could provide more insight into the local microbial environment. However, several studies investigating the impact of AIEC on the microbiota (PMID: 23896971, PMID: 28179509), as well as the influence of materials on gut microbiota (PMID: 38318229, PMID: 40184333), have primarily focused on stool samples.

We fully agree with your observation that analysis of the mucosa-associated microbiota would indeed be highly relevant for this study. In future research, we plan to extend our investigations to include mucosa-associated microbiota. Some studies that could be referenced in this regard include: PMID: 38124090 and PMID: 34812117.

We have acknowledged this in the Discussion section, where we outline plans to explore mucosa-associated microbiota in future studies. The relevant information has been added to the revised manuscript.

Related content

Lines 338-340

To determine whether MOS MGs treatment altered the microbiome, we performed high-throughput gene-sequencing analysis of 16S rRNA in fecal bacterial DNA isolated from the control group, DSS group, MOS group, MGs group, MOS MGs group mice.

Line 668-669

Total DNA was extracted from frozen stool samples using the FastPure Feces DNA Isolation Kit (MJYH, shanghai, China), following the manufacturer's protocols.

Minor Comments

1. Line 73: The authors state that there is a "68.7% increase in total death associated with IBD." As IBD is generally not considered a fatal disease, this statement appears inaccurate. Furthermore, the referenced source (Catch-bond mechanism of the bacterial adhesin FimH) does not support this claim. The reference should be replaced with a more appropriate one.

Response:

We thank the reviewer for the valuable comment. We acknowledge the inaccuracy in our previous statement regarding the mortality associated with IBD. To address this concern, we have revised the sentence to more accurately reflect the public health impact of IBD. (Lines 73-76)

Related content

The rapidly increasing worldwide prevalence of IBD, driven by rising incidence and improved survival, necessitates urgent implementation of preventive measures and therapeutic advancements to mitigate its expanding public health impact.

Additionally, we have replaced the original reference with a more appropriate one: PMID: 37231405. The relevant information has been added to the revised manuscript. (Line 713).

2. Line 95: References 1 and 16 are not suitable for this context. Instead, the authors should cite PMID: 23358328 and PMID: 17525800, which are more relevant to the sentence.

Response:

We thank the reviewer for the valuable comment. Following your recommendation, we have replaced References 1 and 16 with PMID: 23358328 and PMID: 17525800, which are more relevant to the context. The changes have been incorporated into the revised manuscript. (Lines 708,745).

3. Line 123: The phrase "ATPS is formed without oil-free and surfactant-free conditions" is unclear. It is not evident whether oil and surfactants are used in the formation of ATPS. This should be corrected for clarity and grammatical accuracy.

Response:

We thank the reviewer for the valuable comment. We have revised the sentence for clarity and grammatical accuracy.

Related content

Lines 110-113

However, traditional microfluidic fabrication often involves oils and surfactants, limiting biocompatibility. ATPS-based strategy offers an oil-free, surfactant-free alternative, enabling the biofabrication of microgels for applications in tissue engineering, drug screening, and disease modeling.

4. Line 160: The authors claim that "the adsorption of AIEC decreases their abundance without impacting beneficial probiotics." The term probiotics is not appropriate in this context. Please consider revising it to "beneficial microbes" or "beneficial bacteria."

Response:

We thank the reviewer for the valuable comment. We have revised the sentence as follows:

Lines 120-121

By lowering AIEC abundance while sparing ***beneficial microbes****, this approach helps restore gut microbiota balance and potentially alleviates IBD.*

5. Line 169: The manuscript is difficult to follow due to the excessive use of abbreviations. A comprehensive list of abbreviations should be included, and all abbreviations should be spelled out in full the first time they appear (e.g., MOS GelMA MGs on line 169 and in all figure legends).

Response:

We thank the reviewer for the valuable comment. Following your recommendation, we have compiled all abbreviations into a comprehensive abbreviation list (supp table 3) and ensured that each abbreviation is spelled out in full the first time it appears. These revisions have been incorporated into the manuscript.

Related content

Table S3. Abbreviations in this study.

Abbreviations	
AIEC	*Adherent–Invasive E. coli LF82 O83:H7 strain*
WT	*Wild type LF82 bacteria*
IBD	*Inflammatory bowel disease*
MOS	*Mannan oligosaccharides*
GelMA	*Gelatin methacryloyl*
MGs	*GelMA microgels*
MOS MGs	*GelMA microgels containing MOS.*
CFU	*Colony-forming unit*
MOI	*Multiplicity of infection*
FITC	*Fluorescein isothiocyanate isomer I*
PBS	*Phosphate buffered saline*
DSS	*Dextran sulfate sodium salt*
DAI	*Disease activity index*
IVIS	*In Vivo Imaging Systems*
FimH	*The adhesion portion of type 1 fimbriae in Escherichia coli*[1]
ATPS	*Aqueous two-phase system*
PEO	*Polyethylene oxide*
LAP	*Lithium Phenyl-2,4,6-trimethylbenzoylphosphinate*
SGF	*Simulated gastric fluid*
SIF	*Simulated intestinal fluid*
HUVECs	*human umbilical vein endothelial cells*
PCoA	*Principal coordinate analysis*
PLS-DA	*Partial least squares discriminant analysis*
OTU	*Operational taxonomic units*
LEfSe	*Linear discriminant analysis effect size*
LDA	*Linear discriminant analysis*
SPR	*Surface plasmon resonance*
H&E	*Hematoxylin and eosin*

IL-6	*Interleukin-6*
IL-1β	*Interleukin-1 beta*
TNF-α	*Tumor necrosis factor alpha*
ZO-1	*Zona occludens-1*

1 *Zhang, W. et al. Escherichia coli adhesion portion FimH functions as an adjuvant for cancer immunotherapy. Nat Commun* ***11****, 1187, doi:10.1038/s41467-020-15030-4 (2020).*

6. Line 183: Please clarify the meanings of LAP and PEO, as they are not defined. Similarly, define SGF and SIF on line 247.

Response:

We thank the reviewer for the valuable comment. We have included the definitions of "LAP, PEO, SGF, and SIF" in the abbreviation list to ensure clarity. These updates have been incorporated into the revised manuscript.

Related content

Line 129 *Polyethylene oxide (PEO)*

Lines 140-141 *Lithium phenyl-2,4,6-trimethylbenzoylphosphinate (LAP)*

Line 227 *simulated gastric fluid (SGF) and simulated intestinal fluid (SIF)*

7. Line 221: Specify which cell types were used in the experiments.

Response:

We thank the reviewer for the valuable comment. We have added the specified cell types.

Related content

Lines 187-189

Cell viabilities were characterized using Live/Dead staining in ***Caco-2 and HeLa*** *cells, with green fluorescence indicating live cells and red fluorescence indicating dead cells.*

Fig 3 A-B

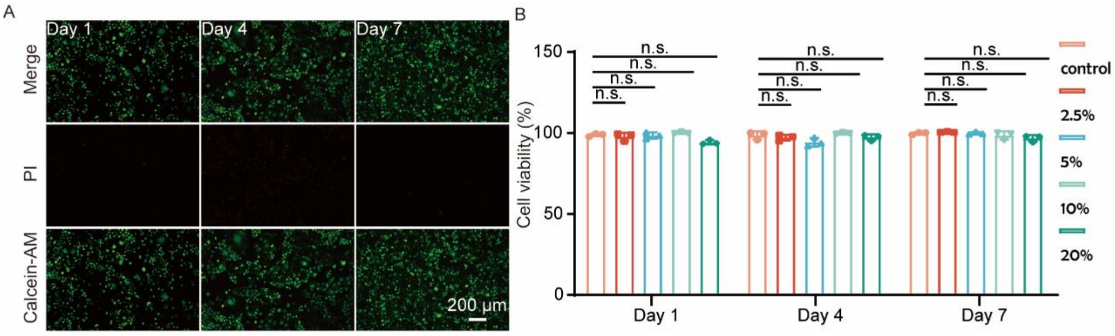

A, B) Biocompatibility assessment of MOS MGs using Caco-2 cells: Live/Dead cell staining (A) and CCK-8 assay (n=3) (B).

Fig S6 A-B

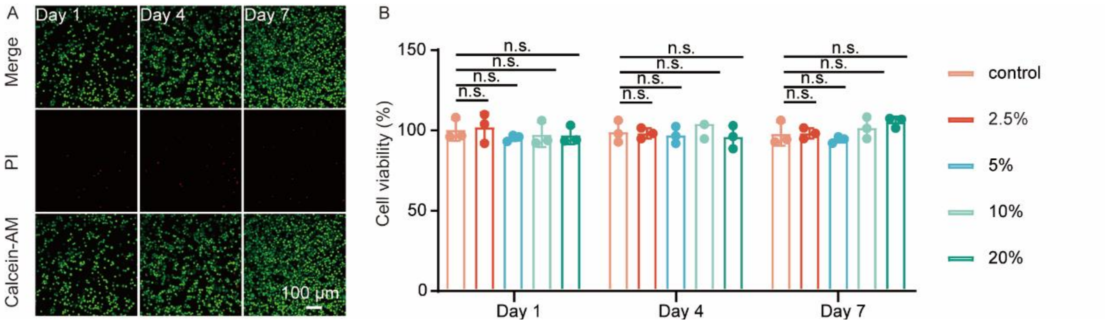

A, B) Biocompatibility assessment of MOS MGs using HeLa cells: Live/Dead cell staining (A) and CCK-8 assay (B) (n=3).

8. Line 266: The term WT is ambiguous. Does this refer to WT LF82 bacteria? The same clarification is needed for line 281.

Response:

We thank the reviewer for the valuable comment. We clarified the meaning of the abbreviation “WT” at its first appearance in lines 160-161, where it refers to wild-type LF82 bacteria. This definition is also included in the list of abbreviations for clarity.

Related content

lines 160-161

wild type AIEC strain LF82 (WT)

9. Line 280: Specify the animal model used in the study.

Response:

We thank the reviewer for the valuable comment. In response, we have supplemented the manuscript by adding the animal model. The revised sentence now reads:

Lines 266-267

We further conducted in vivo experiments for verification using a DSS-induced mouse model of colitis.

10. Line 298: The phrase "very low DAI" seems contradictory to the context. Did the authors mean "very high DAI"? Please revise.

Response:

We thank the reviewer for the valuable comment. As you pointed out, the correct term should be "very high DAI". We sincerely apologize for this oversight, and the manuscript has been corrected accordingly.

Related content

Lines 293-294

*Mice treated with dextran sulfate sodium salt (DSS) showed significant weight loss, reduced intestinal length, and **very high DAI**, as expected.*

11. Line 340: The statement "GelMa MG restores dysbiosis" should be replaced with "GelMa MG prevents dysbiosis" or "GelMa MG favors eubiosis."

Response:

We thank the reviewer for the valuable comment. In response, we have revised the manuscript and changed the statement to: prevents dysbiosis.

Related content

Lines 343-345

*The Chao1 index and Number of observed OTU index were significantly increased in MOS MGs group compared with the DSS group, indicating MOS MGs treatment effectively **prevents dysbiosis** in DSS-induced colitis in mice.*

12. Line 367: The conclusion that "Bifidobacteriaceae were the key type of bacteria contributing to gut microbiota recovery" is unclear. Please elaborate on

how this conclusion was reached based on the data presented.

Response:

We thank the reviewer for the valuable comment. To clarify the conclusion that "*Bifidobacteriaceae* were the key type of bacteria contributing to gut microbiota recovery," we would like to provide a more detailed explanation based on the data presented. The LEfSe (Linear discriminant analysis effect size) method was used to analyze the 16S rRNA sequencing data, which identifies bacterial biomarkers that significantly differentiate between experimental groups. This method helps to pinpoint species with the greatest impact on gut microbiota composition, while Linear Discriminant Analysis (LDA) quantifies their relative effect size. By applying these approaches, we identified *Prevotellaceae* and *Bifidobacteriaceae* as the most significantly altered families across experimental conditions, and the abundance changes of these taxa were strongly correlated with the observed recovery of the gut microbiota.

Bifidobacteriaceae, a well-established probiotic family, is widely recognized for its ability to restore the intestinal barrier and alleviate inflammation, supporting the therapeutic potential of MOS MGs[1,2]. In contrast, although *Prevotellaceae* is often associated with improved carbohydrate metabolism[3,4] and potential benefits for gut health[5-7], its role remains controversial, as certain strains have also been implicated in pro-inflammatory conditions depending on the host context and microbial interactions [8-10]. Therefore, the implications of the observed increase in *Prevotellaceae* abundance warrant further investigation.

In conclusion, the combination of LEfSe analysis and literature-supported functional relevance led us to emphasize *Bifidobacteriaceae* as the key bacterial family contributing to gut microbiota recovery in this study. The relevant information has been added to the revised manuscript.

References

1) PMID: 32319522 2) PMID: 28983638 3) PMID: 38095419 4) PMID: 32928123 5) DOI:10.1016/j.jff.2023.105820 6) PMID: 34258428 7) PMID: 28198087 8) PMID: 30924428 9) PMCID: PMC7790746 10) PMID: 33461110

Related content

Lines 367-384

*To confirm which bacterium was altered by MOS MGs treatment and in turn affected the disease progression against DSS-induced colitis, we performed high-dimensional class comparisons using the linear discriminant analysis (LDA) of effect size (LEfSe) that detected marked differences in the predominance of bacterial communities between five groups. Notably, the relative abundances of Bifidobacteriaceae and Prevotellaceae increased significantly. Bifidobacteriaceae, a well-established probiotic family, is widely recognized for its ability to restore the intestinal barrier and alleviate inflammation, supporting the therapeutic potential of MOS MGs[28,29]. In contrast, although Prevotellaceae is often associated to improved carbohydrate metabolism[30,31] and potential benefits for gut health[32-34], its role remains controversial, as certain strains have also been implicated in pro-inflammatory conditions depending on the host context and microbial interactions [35-37]. Despite this, the concurrent reduction in Enterobacteriaceae—a bacterial family closely linked to intestinal inflammation and dysbiosis—suggests that MOS MGs not only suppress pathogenic bacteria but also contribute to microbiome rebalancing and inflammation alleviation. (**Fig. 7K**).*

In conclusion, the treatment of MOS MGs can significantly enhance the relative abundance of Bifidobacteriaceae and reduce the relative abundance of Enterobacteriaceae, thereby mitigating inflammation in DSS-induced colitis.

13. Discussion Section: The discussion is underdeveloped and reads more like a conclusion. It should be expanded to compare the authors' findings with relevant studies in the literature that used strategies to clear AIEC bacteria. The authors should also discuss how their approach is advantageous or complementary to existing methods.

Response:

We thank the reviewer for the valuable comment. Based on these, we have revised the Discussion section as follows:

Lines 385-448

Discussion

The escalating incidence and mortality rates associated with IBD underscore the urgent need for effective therapeutic strategies. The multifactorial etiology of IBD, involving the interplay of genetic, environmental, and microbial factors creates a complex treatment landscape. Although significant progress has been made in the treatment of IBD, several challenges and limitations remain, which impact both treatment efficacy and patient quality of life. Traditional therapeutic approaches, such as immunosuppressants, corticosteroids, and biologics, while effective in alleviating symptoms, are associated with notable side effects[38]. For instance, immunosuppressants may suppress the immune system, increasing the risk of infections[39]; prolonged use of corticosteroids may lead to osteoporosis, weight gain, and hyperglycemia[40]; and biologics may trigger allergic reactions or the development of drug resistance[41]. These side effects not only increase the health burden on patients but may also affect their adherence to treatment. Furthermore, current therapies can disrupt the normal balance of the gut microbiome[42]. Long-term use of antibiotics or immunosuppressants can disrupt the gut microbiome balance, causing microbial dysbiosis and a reduction in beneficial bacteria, which leads to immune system dysregulation and intensifies inflammatory responses, ultimately exacerbating disease progression. Therefore, developing new therapeutic strategies, particularly those that are more precise, personalized, and capable of reducing side effects, remains a critical goal in the field of IBD treatment.

To address the limitations of current therapies, researchers have begun exploring more targeted approaches. Compelling evidence suggests that AIEC is closely associated with the pathogenesis of CD, and given its pivotal role in inflammation, FimH, a key adhesin of AIEC, has emerged as a novel therapeutic target for IBD, gaining increasing attention in the field. Some studies have attempted to develop inhibitors targeting FimH, such as small molecule antagonists or antibodies, aiming to block the interaction between FimH and gut cells. These studies suggest that inhibiting FimH activity could effectively alleviate symptoms in IBD patients and reduce common

side effects of current treatments, such as immune system suppression and dysbiosis[43,44]. *However, the pharmacokinetics of FimH inhibitors remain poorly understood, including their absorption, metabolic stability, and excretion. Moreover, as most FimH inhibitors are mannoside-based, they may be metabolized by the gut microbiota as a carbon source.*

To overcome the limitations of current FimH-targeted therapies, we developed a novel oral microgel-based engineering carrier for MOS delivery and immobilization. Microgels offer robust protection for MOS against degradation in the harsh gastric environment and prevent its premature metabolism by the gut microbiota as a carbon source, thereby reducing the risk of microbial dysbiosis. The microgels ensure that MOS is delivered intact to the intestinal tract, where it exerts its therapeutic effects and is ultimately excreted without systemic absorption. Importantly, MOS MGs demonstrated therapeutic efficacy comparable to commercially available FimH inhibitors such as M4284. However, unlike traditional inhibitors that merely block AIEC adhesion to intestinal epithelial cells, MOS MGs not only inhibit adhesion but also physically trap and remove adherent AIEC from the gastrointestinal tract, resulting in more effective bacterial clearance.

To implement this oral delivery strategy, we employed an innovative fabrication approach that integrates an ATPS-based strategy with microfluidic technology. In contrast to conventional oil-phase microfluidic methods, this ATPS-based strategy eliminates the use of organic solvents and surfactants, significantly enhancing the biosafety and in vivo applicability of the microgels. The resulting porous microgel exhibits stable structures, precise tunability, and high biocompatibility, providing an ideal platform for MOS encapsulation and targeted intestinal delivery, thereby enhancing its potential for clinical translation.

Moreover, the targeting mechanism of this microgel platform offers broad therapeutic potential, as FimH is a highly conserved adhesin found in a variety of pathogenic bacteria, including Enterobacter, Klebsiella, Salmonella, Shigella, and uropathogenic E. coli. By interfering with FimH-mediated adhesion, this system has significant potential as a localized, microbiota-friendly, and low-resistance therapeutic

alternative for a range of FimH-expressing bacterial infections. Future studies should aim to further optimize the structure of microgels for enhanced targeting efficiency, evaluate potential synergies with existing treatments, and comprehensively assess the effects of this system on mucosa-associated microbiota, ultimately contributing to the development of precise and personalized IBD therapies.

14. Line 471: The authors describe the strategy for overexpressing and purifying FimH but it is not clear in which figure this approach is presented. Please ensure this is clearly indicated, and revise the figure legends to include more methodological details to help readers understand what is shown in the panels.

Response:

We thank the reviewer for the valuable comment. The part related to FimH purification is presented in Fig 4 A-C. We have revised the figure legend to include more methodological details.

Related content

The figure legend of Fig 4 A-C:

The purified FimH protein was subjected to SPR experiments to detect its molecular interaction with mannose (A), MOS (B), and glucose (C).

15. Line 537: Clarify whether the microgels were administered to mice via drinking water or oral gavage. If the latter, specify the volume administered to each mouse.

Response:

We thank the reviewer for the valuable comment. We have clarified that the microgels were administered via oral gavage and specified the total volume per mouse (100 μL).

Related content

Lines 614-617

Starting from the first day of the DSS challenge, 0.5% MOS, 1% MGs, 1% MOS MGs (containing 0.5% MOS) or M4284 (100 mg/kg) along with AIEC, were administered to mice via oral gavage at the indicated dose (CFU = 2 × 10^9/mouse/day)

in a total volume of 100 μL per mouse.

Reviewer #2 (Remarks to the Author):

The manuscript entitled "Fishing for AIEC: Using Microgels to Targeted Attract FimH for Combating IBD" proposed the concept of competitive adsorption by leveraging the binding properties of FimH with mannose, prompting microgels competitively absorbing AIEC over intestinal epithelial cells for the treatment of IBD.

There are a lot of questions worth considering and addressing, so major revision should be conducted before further consideration.

Response:

We sincerely appreciate your time and effort in reviewing our manuscript entitled "Fishing for AIEC: Using Microgels to Target FimH for Combating IBD." Based on your valuable feedback, we have carefully revised our manuscript and addressed all concerns accordingly.

1. The role of Fig. 1 is very critical, but it still needs to be beautified and visualized by the authors.

Response:

We thank the reviewer for the valuable comment. We have further optimized the figure to enhance its clarity and visual appeal, ensuring better readability and comprehension. The relevant information has been added to the revised manuscript.

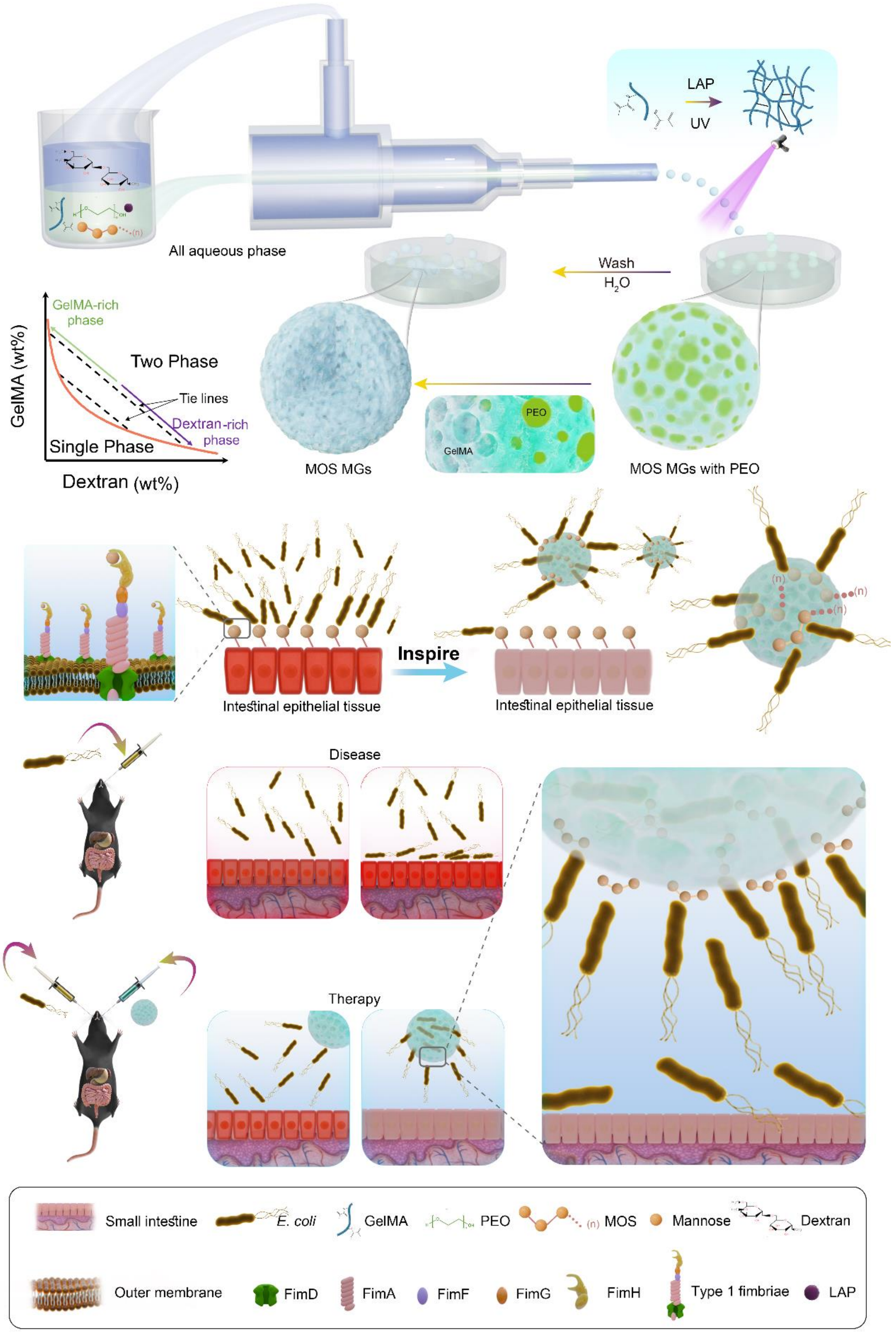

***Fig. 1** Schematic illustration of porous MOS MGs fabrication by merging all-aqueous and co-axial microfluidic technologies and prompting microgels competitively absorbing AIEC over intestinal epithelial cells for the treatment of IBD by leveraging the binding properties of FimH with mannose.*

2. The full name of AIEC does not appear in the abstract and introduction.

Response:

We thank the reviewer for the valuable comment. We have added the full name of AIEC (adherent-invasive *Escherichia coli*) in both the abstract and introduction to ensure clarity. The relevant information has been added to the revised manuscript.

Related content

Line 58

absorbing adherent-invasive Escherichia coli (AIEC)

3. In addition to AIEC, FimH is also present on the surface of other Enterobacteriaceae bacteria, including but not limited to Enterobacter, Klebsiella, Salmonella, Shigella, etc. The author need to discuss about this.

Response:

We thank the reviewer for the valuable comment. We agree that discussing the presence of FimH in other *Enterobacteriaceae* bacteria is important for highlighting the broader applicability of our system. The relevant information has been added to the discussion section of the revised manuscript. We hope this addition adequately addresses your concern.

Related content

Lines 439-448

Moreover, the targeting mechanism of this microgel platform offers broad therapeutic potential, as FimH is a highly conserved adhesin found in a variety of pathogenic bacteria, including Enterobacter, Klebsiella, Salmonella, Shigella, and uropathogenic E. coli. By interfering with FimH-mediated adhesion, this system has significant potential as a localized, microbiota-friendly, and low-resistance therapeutic alternative for a range of FimH-expressing bacterial infections. Future studies should aim to further optimize the structure of the microgels for enhanced targeting efficiency, evaluate potential synergies with existing treatments, and comprehensively assess the effects of this system on mucosa-associated microbiota, ultimately contributing to the development of precise and personalized IBD therapies.

4. The introduction section is too long. The author provides too much information on the background and synthesis of the material. The important role of the introduction is to effectively guide the readers into the theme of the paper and help the readers understand the importance and necessity of the research. Therefore, only a brief introduction to the design and synthesis methods is needed.

Response:

We thank the reviewer for the valuable comment. In response, we have removed unnecessary background information and condensed the introduction to focus on guiding readers into the core theme of the paper, emphasizing the importance and necessity of our research while providing only a brief overview of the design and synthesis methods.

Related content

Lines 70-121

Introduction

Inflammatory bowel disease (IBD) is an immune-mediated intestinal disorder with complex pathophysiological mechanisms involving genetic, environmental, microbial, and immunological factors[2]. The rapidly increasing worldwide prevalence of inflammatory bowel disease (IBD), driven by rising incidence and improved survival, necessitates urgent implementation of preventive measures and therapeutic advancements to mitigate its expanding public health impact[3]. The primary types of IBD include ulcerative colitis (UC), Crohn's disease (CD), and indeterminate colitis (IC). Unlike common intestinal inflammation, which is typically acute and curable with anti-infective treatment, IBD is characterized by a prolonged course with recurrent episodes and remains incurable to date[4,5]. The clinical manifestations of IBD are varied, encompassing gastrointestinal symptoms such as diarrhea, abdominal pain, bloody stools, and perianal abscesses[6]. The etiology of IBD is multifactorial, including psychological stress, autonomic nervous system dysfunction, dysbiosis of gut microbiota, and immune regulation associated with disease activity[7]. The primary characteristics of gut microbiota imbalance are an increase in mucosa-associated

bacteria and a decrease in overall biodiversity[8]*. Although no single pathogenic microorganism has been definitively identified, many studies have reported that alterations in pathogens and commensal bacteria may be involved in the disease's pathogenesis*[9]*.*

Among these bacteria, adherent-invasive Escherichia coli (AIEC) is increasingly recognized as a major player in the pathogenesis of CD[10-12]*. AIEC plays a crucial role in the pathogenesis of CD through its ability to adhere to and invade intestinal cells, survive within macrophages, induce pro-inflammatory responses, disrupt the intestinal barrier, and contribute to gut dysbiosis*[13]*. Upon entering the intestine, AIEC strains adhere and invade epithelial cells via type 1 pili adhesin FimH, which has important clinical significance in the pathogenesis of CD*[14]*. Specifically, FimH directly binds to mannose residues on carcinoembryonic antigen-related cell adhesion molecule 6 (CEACAM6) glycoproteins present on the surface of intestinal epithelial cells, which facilitates the initial attachment of AIEC to the host cells*[15,16]*.*

The treatment of IBD still faces significant challenges, as most current targeted strategies exhibit limited therapeutic efficacy and are often associated with adverse effects and the emergence of drug resistance[17]*. Recent studies have shown that disrupting the crucial interactions among CEACAM6, FimH, and mannose can effectively reduce the pathogenicity of AIEC, offering a promising approach for the management of CD*[18]*. However, these therapeutics often suffer from poor target selectivity, limited bioavailability, or the need for repeated administration, and their stability and safety in the complex intestinal environment remain insufficient*[20]*.*

Microgels, micron-scale granular hydrogels, have gained significant attention due to their small size, high flexibility, good injectability, porosity, and large specific surface area. Such unique characteristics, make them a promising option for use as carriers in drug delivery and cell therapy. However, traditional microfluidic fabrication often involves oils and surfactants, limiting biocompatibility. The ATPS-based strategy offers an oil-free, surfactant-free alternative, enabling the biofabrication of microgels for wide-ranging applications in tissue engineering, drug screening, and disease modeling.

Here, as schematically briefed in Fig. 1, we present a robust method for

fabricating porous microgels loaded with Oligosaccharides (MOS, an oligomer of mannose) by merging all-aqueous and co-axial microfluidic technologies. Instead of directly delivering drugs, which may lead to toxicity or resistance, we propose a competitive adsorption strategy, where microgels, by leveraging the specific binding affinity of FimH to mannose, selectively capture AIEC and reduce its colonization of intestinal epithelial cells. By lowering AIEC abundance while sparing beneficial microbes, this approach helps restore gut microbiota balance and potentially alleviates IBD.

5. The name of the material "MOS Gelma MGs" is too long and can be appropriately abbreviated.

Response:

We thank the reviewer for the valuable comment. We have revised the term “MOS GelMA MGs” to the abbreviated form “MOS MGs” throughout the manuscript. This abbreviation has also been added to the list of abbreviations for clarity.

6. Animal experiment require appropriate positive control groups to judge the actual efficacy of MOS Gelma MGs, which includes treatment with fimH inhibitors or first-line drug 5-ASA.

Response:

We thank the reviewer for the valuable comment. In response, we have included an appropriate positive control group by selecting M4284 as a FimH inhibitor (PMID: 28614296). The experimental results are presented in Fig. 4G-I, Fig. 5, and Fig. 6. The data showed that, compared to M4284, the MOS MGs treatment group exhibited a similar level of AIEC colonization reduction in both the ileum and colon. Notably, the MOS MGs group demonstrated even lower AIEC counts in fecal samples, which may be attributed to the dual function of MOS MGs—not only inhibiting AIEC adhesion, but also adsorbing and facilitating its removal from the gastrointestinal tract. Furthermore, in the DSS-induced colitis model, the MOS MGs group achieved comparable therapeutic efficacy to M4284, indicating that MOS MGs are effective in alleviating inflammation and restoring intestinal health. Relevant data have also been

included in this response below for your reference.

Related content

Lines 272-279

No significant differences in bacterial colonization were observed in the MOS or MGs treatment groups compared to the control group. In contrast, treatment with M4284 led to a significant reduction of AIEC colonization in the ileum and colon, but not in the feces. Notably, mice treated with MOS MGs exhibited a significant decrease in AIEC levels in the ileum, colon, and feces. These results indicate that MOS MGs, beyond mimicking the anti-adhesive function of M4284, also exert an active bacterial-trapping effect, thereby promoting the effective clearance of AIEC from the intestinal tract.

Fig.4 G-I:

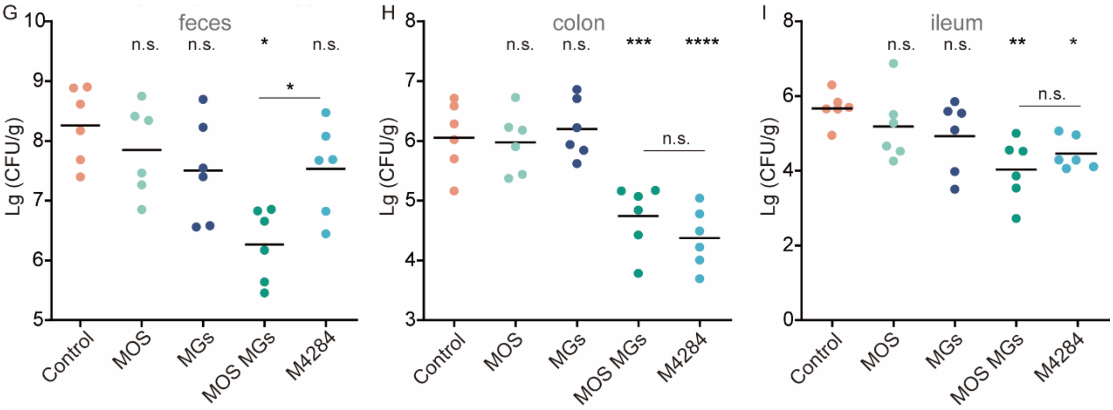

G–I) Colonization levels of WT in feces (G), colon (H), and ileum (I) after treatment with MOS, MGs, MOS MGs, and M4284 (n=6).

Lines 293-300

Mice treated with dextran sulfate sodium salt (DSS) showed significant weight loss, reduced intestinal length, and very high DAI, as expected[24]*, there was no significant difference between the MGs group, MOS group and the DSS group while both the M4284 group and the MOS MGs group showed varying degrees of therapeutic improvement. Of note, colitis model animals treated with MOS MGs or M4284 exhibited increased colon length than those treated with groups of MOS, MGs, and DSS (**Fig. 5B, C**) and significantly less weight loss (**Fig. 5D**), a lower DAI (**Fig. 5E**).*

Fig 5:

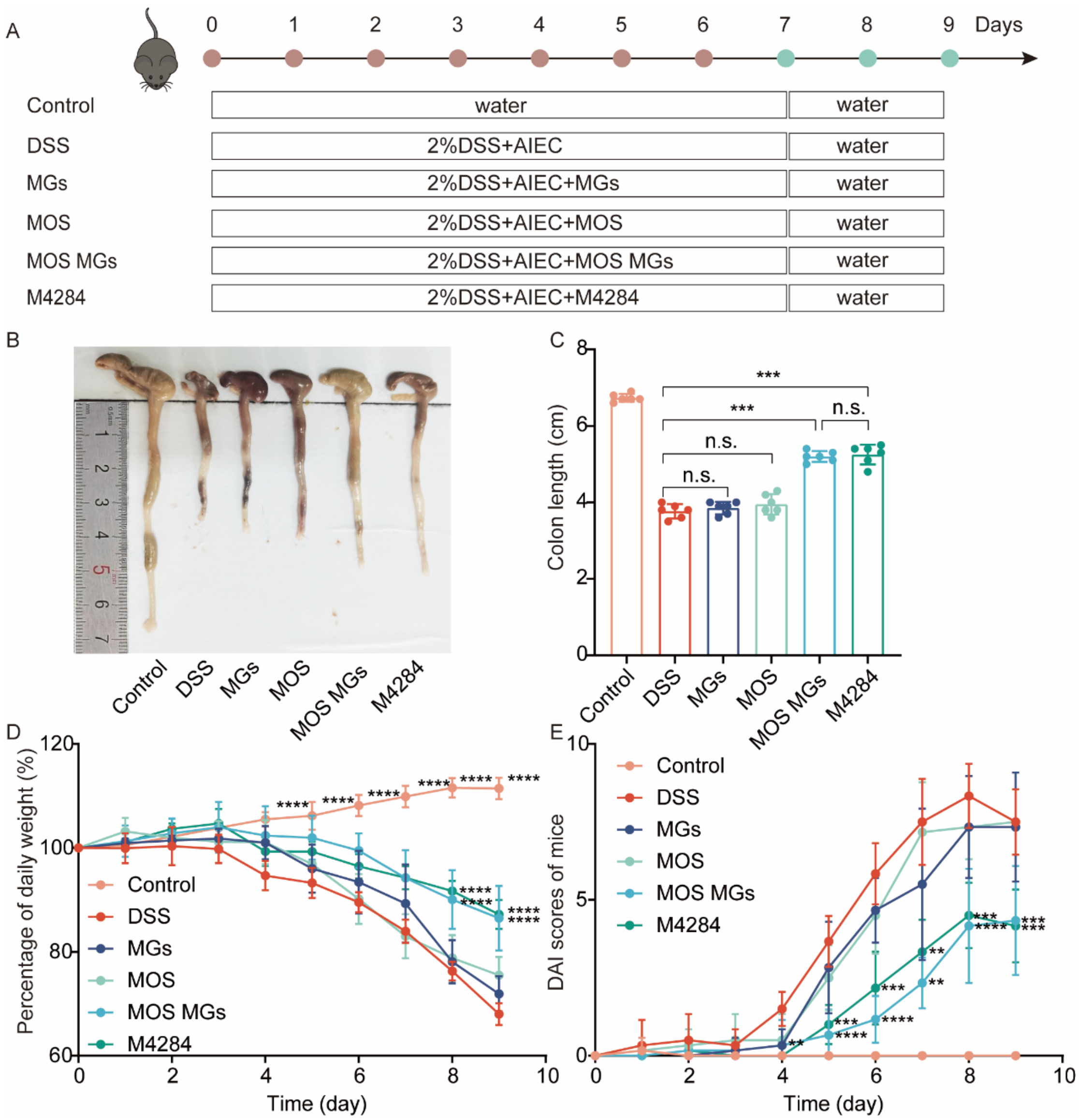

*A) Schematic illustration of model building and intervention. B) Macroscopic colon appearance of each group was shown (n=6). C) Colon length was measured and analyzed (n=6). D) Daily changes in body weight were recorded in detail and analyzed (n=6). E) Daily DAI scores were calculated and analyzed (n=6). Significance was determined by two-tailed unpaired Student's t test (C, D, E) and indicated as the P value; ns, not significant; * $p < 0.05$, ** $p < 0.01$, *** $p < 0.001$, **** $p < 0.0001$. Data are presented as mean ± s.d. (C, D, E).*

Lines 300-329

We further verified the efficacy of MOS MGs by hematoxylin and eosin (H&E) staining of colon tissue. According to the colon damage score from the H&E staining

*in **Fig. 6A-F**, treatment of MOS MGs also helped maintain the integrity of colon epithelium and lessen the infiltration of pro-inflammatory cells of the mucosa, exhibiting a therapeutic effect comparable to that of M4284.*

*Next, we subsequently conducted a detailed analysis to elucidate the mechanism by which MOS MGs protect mice from DSS-induced colitis. First, we evaluated whether MOS MGs could improve intestinal epithelial barrier function. To this end, we assessed intestinal permeability using Dextran-FITC and examined the expression of ZO-1. ZO-1 is a tight junction protein that forms tight connections between epithelial cells in the intestine, thereby regulating the permeability of the intercellular space. In patients with IBD, ZO-1 expression and function are often compromised, leading to disruption of the intestinal mucosal barrier and increased permeability to bacteria and toxins[25]. Compared to the DSS group, treatment with MGs led to a certain reduction in intestinal permeability. However, both the MOS MGs and M4284 treatment groups showed a marked decrease in permeability (**Fig. 6H**). Furthermore, mice treated with MOS MGs exhibited a significant upregulation of ZO-1 gene expression, indicating that MOS MGs effectively enhance intestinal epithelial barrier function (**Fig. 6I**).*

*Moreover, we selected IL-6, IL-1β, and TNF-α as targets for analysis and compared the gene expression levels of these three inflammatory factors in the small intestines of mice across four different treatment groups. IL-6, IL-1β, and TNF-α play crucial roles in intestinal inflammation by acting as pro-inflammatory cytokines that promote the initiation and persistence of inflammatory responses[26,27]. Dysregulated expression and modulation of these cytokines may contribute to the chronicity and exacerbation of inflammatory reactions. As expected, RT-PCR experimental results are consistent with our previous findings in vitro. Treatment with M4284 or MOS MGs significantly downregulated pro-inflammatory genes (IL-6, IL-1β, TNF-α) compared to the DSS group, with MOS MGs showing the most pronounced effect (**Fig. 6J-L**). In contrast, neither MOS nor MGs alone significantly altered these inflammatory factors relative to DSS controls.*

Fig. 6

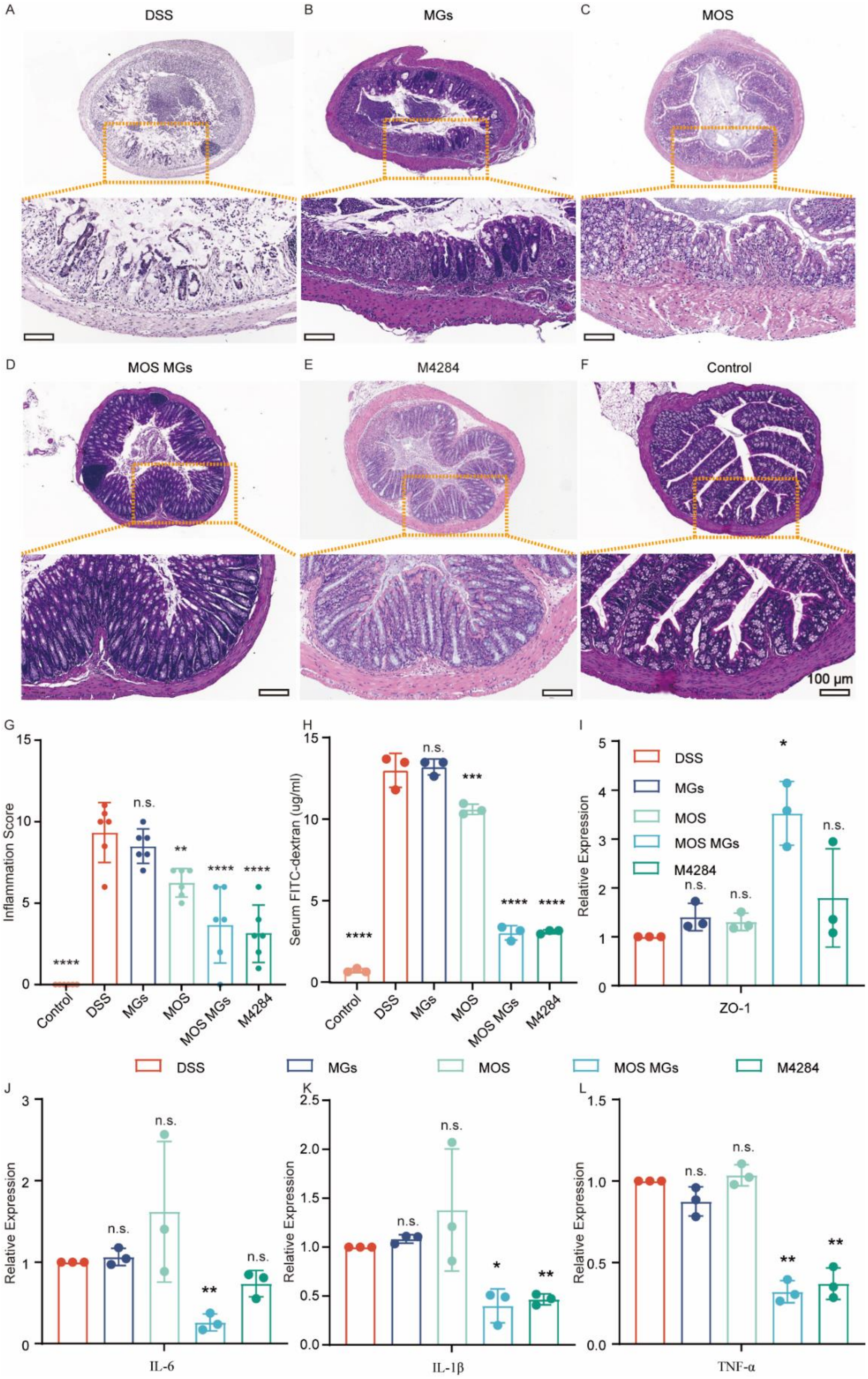

A-F) Representative hematoxylin and eosin (H&E) staining images of colon tissue of each group. G) Colonic damage scores according to H&E staining were analyzed in each group (n=6). H) Intestinal permeability assessment using FITC-dextran (n=3). I-

*L) Colonic mRNA levels of ZO-1, IL-6, IL-1β, and TNF-α, (n=3). Significance was determined by two-tailed unpaired Student's t test (G-L) and indicated as the P value; ns, not significant; * $p < 0.05$, ** $p < 0.01$, *** $p < 0.001$, **** $p < 0.0001$. Data are presented as mean ± s.d.*

7. Why was the biosafety experiment conducted using HeLa cells? It should be conducted on intestinal epithelial cell line.

Response:

We thank the reviewer for the valuable comment. Our initial choice of HeLa cells was intended to provide a general assessment of the material's cytocompatibility. HeLa cells were chosen for the biosafety experiment as they are commonly used in cytotoxicity studies to provide an initial assessment of material biocompatibility (PMID: 24980623, PMID: 32003926). However, we acknowledge that using an intestinal epithelial cell line would be more appropriate for evaluating the biosafety of MOS GelMA MGs in the context of IBD treatment. In response, we have repeated the experiment using Caco-2 cells, a well-established model for intestinal epithelium. The results were consistent with those obtained from HeLa cells, further confirming the excellent cytocompatibility of the material. (Figure 3A, B, and Figure S6A, B;)

Related content

Lines 186-195

Subsequently, we evaluated the biocompatibility of MOS MGs both in vitro and in vivo. Initially, an in vitro biocompatibility assessment was performed. Cell viabilities were characterized using Live/Dead staining in Caco-2 and HeLa cells, with green fluorescence indicating live cells and red fluorescence indicating dead cells. The cells were cultivated for up to 7 days. It was found that the cell viability remained as high as 90% during the entire course of culture, and the group of MOS MGs (Fig. 3A, Fig. S6A) showed no significant changes with other groups in cell viability over time. Colorimetric assays were conducted on varying concentrations of these MGs in the Caco-2 and HeLa cell lines. The results indicated no significant cellular toxicity across concentrations ranging from 0 to 20% for MOS MGs (Fig. 3B, Fig. S6B).

Fig 3 A-B

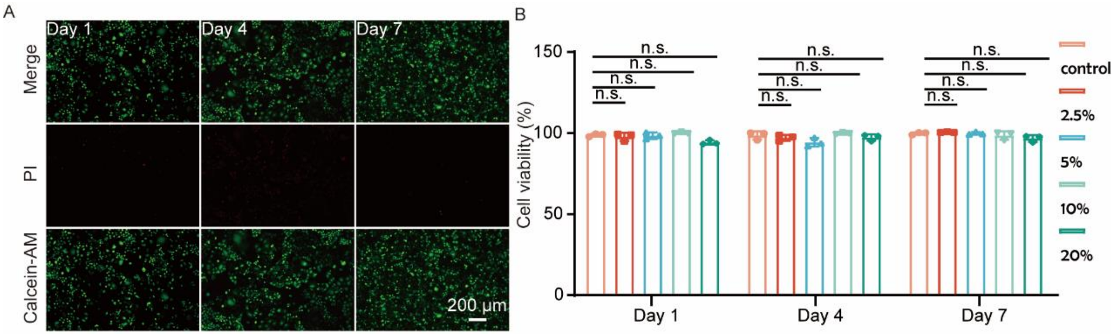

A, B) Biocompatibility assessment of MOS MGs using Caco-2 cells: Live/dead cell staining (A) and CCK-8 assay (n=3) (B).

Fig S6 A-B

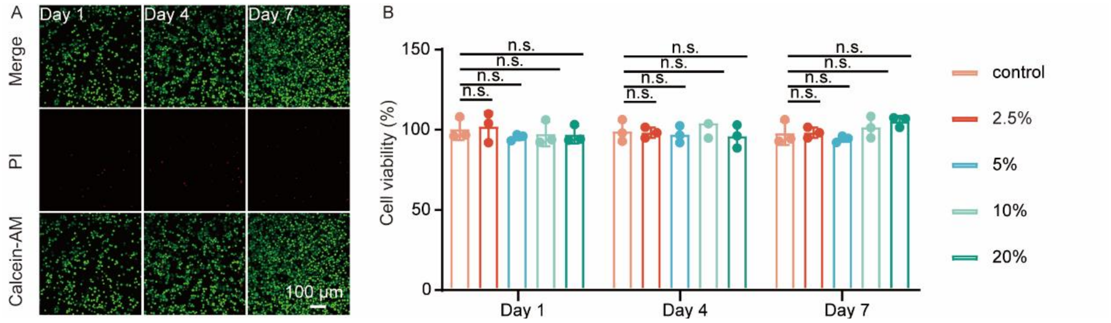

A, B) Biocompatibility assessment of MOS MGs using HeLa cells: Live/dead cell staining (A) and CCK-8 assay (B) (n=3).

8. According to the scale bar in Figure 4D, the diameter of the microgel is 100 micrometers, but in Figure 2, the diameter of the microgel is 500 micrometers, which is very confusing.

Response:

We thank the reviewer for the valuable comment. To provide a clearer visualization of the porous structure, we selected larger microgels in Figure 2. However, for our experiments, we used microgels with a diameter of approximately 100–150 μm to facilitate oral gavage. To ensure consistency, both the cell and animal experiments were conducted using microgels of the same size.

Related content

Lines 247-249

Considering the injectability required for subsequent in vivo experiments and to

ensure consistency between in vitro and in vivo studies, microgels with a particle size of 100–150 μm were selected for all subsequent experiments.

9. The author needs to provide more evidence to prove the competitive adsorption of the microgel on AIEC. In vitro experiments should co-incubate AIEC, intestinal epithelial cells, and MOS Gelma MGs, and then analyze the proportion of AIEC adhering to intestinal epithelial cells and MOS Gelma MGs.

Response:

We thank the reviewer for the valuable comment. In the revised manuscript, we have conducted additional in vitro competitive adsorption experiments. Specifically, we co-incubated Caco-2 cells with AIEC in the presence of MGs and MOS MGs. We then assessed bacterial adhesion by performing plate counting assays and confocal microscopy to evaluate changes in the number of bacteria adhering to the cell surface after microgel addition. The results showed that the number of AIEC adhering to the cell surface was significantly reduced following the addition of MOS MGs. These results further support the competitive adsorption ability of the microgels toward AIEC. The relative information has been added to the revised manuscript.

Related content

Lines 260-265

*To evaluate competitive adhesion inhibition, Caco-2 cells were co-incubated with AIEC in the presence of MOS MGs or MGs. Results from both plate counting (**Fig. 4E**) and confocal microscopy (**Fig. 4F**) consistently demonstrated that the number of bacteria adhering to the cell surface was significantly reduced in the MOS MGs group compared to the control group.*

Fig 4 E-F

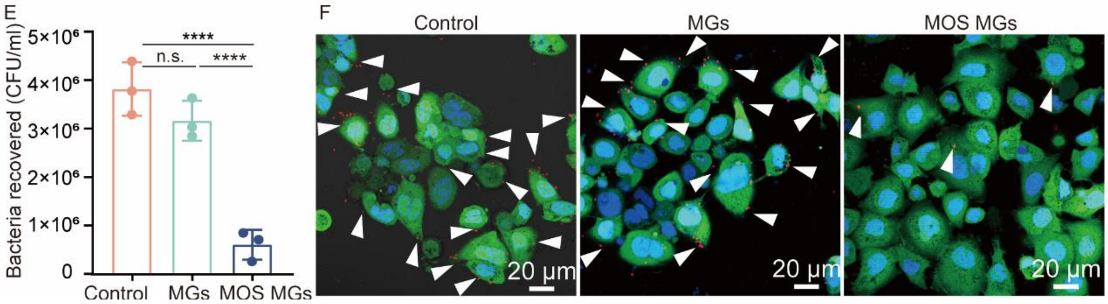

E) Competitive adhesion assay of AIEC between MOS MGs and Caco-2 cells, visualized by colony-forming unit (CFU) plating (n=3). F) Confocal imaging of competitive adhesion of AIEC between MOS MGs and Caco-2 cells. Red: AIEC; blue: DAPI-stained nuclei; green: Calcein-AM-stained cells; white arrows indicate AIEC.

10. In Figure 4G, how did the author detect the colonization of AIEC in the intestinal epithelium? Was the sample feces or intestinal mucosal tissue? The research method for this data was not written in the methods section.

Response:

In our previous version, the colonization analysis was indeed limited to fecal samples. However, in the revised manuscript, we have expanded the evaluation to include bacterial colonization levels in feces, colon, and ileal mucosal tissues, as shown in Figures 4G–I and Figure S8. These additions provide a more comprehensive assessment of AIEC colonization in the gastrointestinal tract. To address this concern, we have now added the corresponding description to the Methods section for clarity.

Related content

Lines 602-609

Mouse infection

Seven to eight-week-old SPF female C57BL/6J mice were orally pretreated with streptomycin (20 mg intragastric per mouse) to disrupt intestinal microbiota. Twenty-four hours later, the mice were challenged with 10^9 AIEC bacteria. After 48h, fresh feces and intestinal tissues were collected and homogenized in PBS. Bacteria were numbered by plating appropriate dilutions on LB agar medium containing the appropriate antibiotics to select and isolate AIEC bacteria and incubated overnight at 37℃.

Fig 4G-I

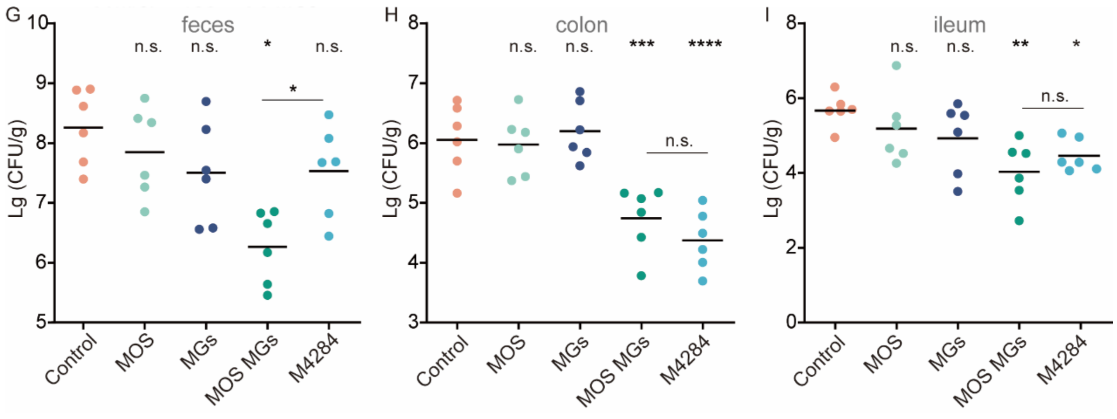

G–I) Colonization levels of WT in feces (G), colon (H), and ileum (I) after treatment with MOS, MGs, MOS MGs, and M4284 (n=6).

Fig S8

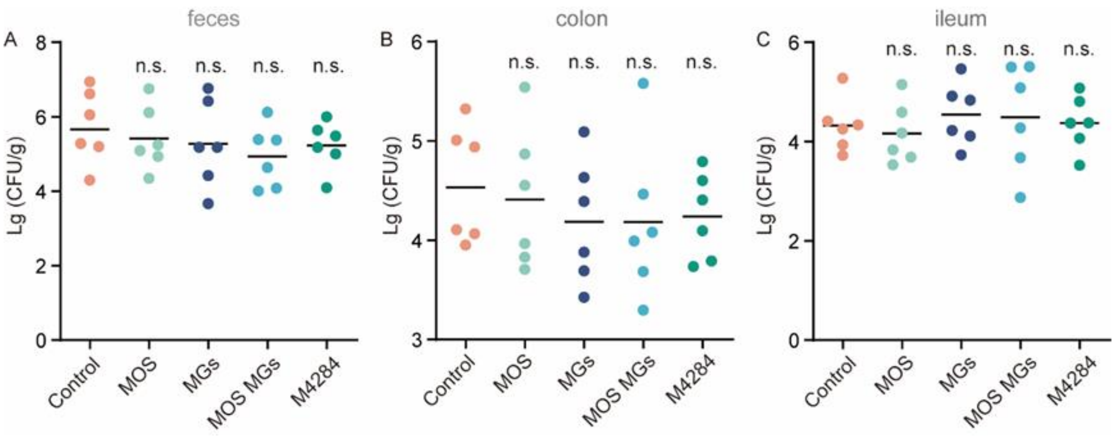

A–C) Colonization levels of ΔfimH in feces (A), colon (B), and ileum (C) after treatment with MOS, MGs, MOS MGs, and M4284 (n=6).

11. In the animal model, oral gavage of a large amount of AIEC (2×10^9/mouse/day) daily was planned. Is the gavage amount of MOS Gelma MGs sufficient to adsorb all the AIEC?

Response:

We thank the reviewer for the valuable comment. As shown in Fig. 4G–I, the oral gavage dose of MOS MGs used in our study was not sufficient to adsorb the entire inoculated AIEC population. However, the experimental results indicate that approximately 90% of AIEC could be adsorbed and subsequently cleared from the

gastrointestinal tract. Moreover, compared to the positive control group, the selected dosage of MOS MGs achieved comparable therapeutic outcomes, demonstrating its effectiveness at the administered level. Relevant data have also been included in this response below for your reference.

Fig 4G-I

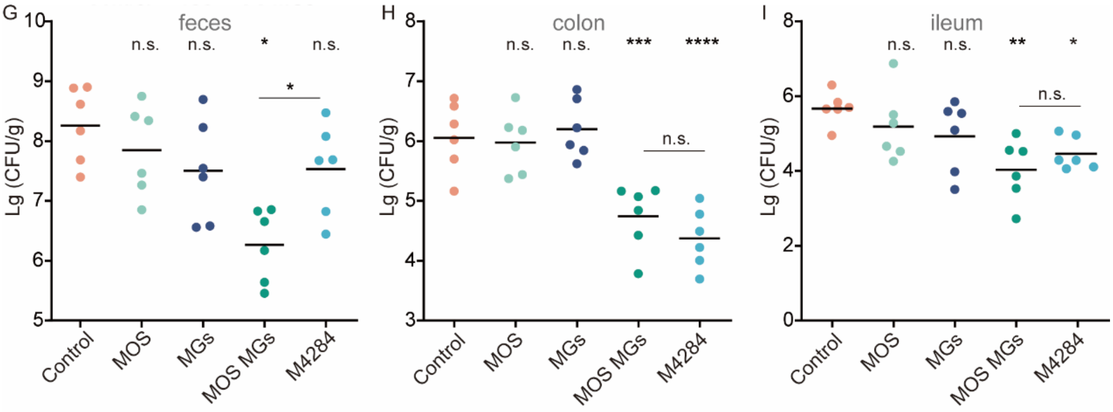

G–I) Colonization levels of WT in feces (G), colon (H), and ileum (I) after treatment with MOS, MGs, MOS MGs, and M4284 (n=6).

12. Has the author ever detected the baseline abundance of AIEC in the intestine, as the survival rate of AIEC is theoretically significantly reduced after passing through gastric fluid?

Response:

We thank the reviewer for the valuable comment. In response to your comment, we have now included an assessment of the baseline abundance of AIEC in the intestine to account for potential reductions in AIEC survival after exposure to gastric fluid. The corresponding mouse data can be found in Figure 4G-I, the bacterial load after passage through the stomach was approximately 1×10^8 CFU/g. Relevant data have also been included in this response below for your reference and marked with red boxes.

Fig 4 G-I

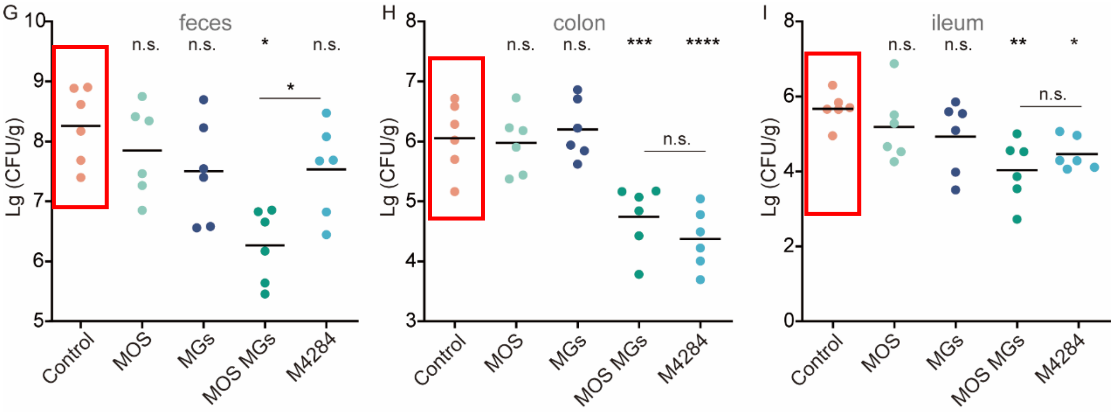

G–I) Colonization levels of WT in feces (G), colon (H), and ileum (I) after treatment with MOS, MGs, MOS MGs, and M4284 (n=6).

Reviewer #3 (Remarks to the Author):

This manuscript by Wu et al designed the porous microgels in order to 'fishing for AIEC' and investigated their advantages and mechanism in inflammatory bowel disease treatment. However, competitively selective bacterial capture is not new, which have been reported in many other papers (ACS Appl. Mater. Interfaces 2022, 14, 42, 47323–47344). More importantly, this paper requires more solid evidence and improvement to show the fate of microgels after absorbing AIEC. In my opinion, this paper is not suitable for publication in Nature Communications. Please refer to the detailed comments below:

Response:

We sincerely thank the reviewer for the time and effort devoted to evaluating our manuscript and for providing constructive feedback. We respectfully address the concerns raised and clarify the novelty and scientific contributions of our work. While we acknowledge the reviewer's opinion regarding the manuscript's suitability for *Nature Communications*, we respectfully argue that our work offers a mechanistically distinctive and translationally relevant approach to modulating host–microbe interactions in IBD via engineered materials. We believe these contributions will be of broad interest to the materials and biomedical community.

We hope the additional data and clarifications adequately address the reviewer's concerns and help reframe the novelty and significance of our work.

1. The porous microgels were fabricated via an aqueous two-phase system. Please give more evidence to confirm its advantages. And the size and distribution of the microgels' porosity should be estimated.

Response:

We thank the reviewer for their valuable comment. Conventional microfluidic methods for microgel fabrication typically require oil and surfactants to achieve droplet

formation. However, these components introduce concerns regarding residual toxicity, as remaining oil or surfactants can cause cytotoxicity (PMID: 34123409). Furthermore, employing oil makes its subsequent removal troublesome, not to mention that the washing step will cause cell damage and may adversely affect cell viability. In contrast, our ATPS-based approach entirely eliminates the need for both an oil phase and surfactants, circumventing these critical issues. To highlight the advantages of our ATPS-based approach, we conducted comparative experiments between microgels fabricated using the traditional oil-phase microfluidics method and those fabricated via the ATPS-based approach. The experimental results showed that the microgels fabricated using the ATPS-based approach exhibited significantly higher cell compatibility compared to those fabricated using the oil-shearing method. (Fig S6C, D). We have incorporated new data demonstrating the precise control over microgel porosity achievable by adjusting the concentration of the discontinuous pore-forming phase within the ATPS. These data, which include size distribution analyses and quantitative porosity measurements, are now included in the revised manuscript.
(Fig S3I, J).

Relevant content

Lines 196-207

To further emphasize the versatility and superiority of our proposed method, we compared cell culture performance using different types of microgels: conventional homogeneous microgels prepared by traditional oil-phase microfluidics, and microgels prepared by ATPS-based strategy. As shown in Fig. S6C, D, the microgels fabricated via our ATPS-based strategy exhibited significantly higher cell viability on days 1, 4, and 7 compared to those produced by conventional oil-shearing microfluidics. Additionally, the expression of functional proteins was markedly enhanced. Furthermore, when porous microgels generated via an ATPS-based strategy were used as carriers, both cell viability and functional protein expression were further improved. These results clearly demonstrate that, compared to conventional approaches, our method offers substantial advantages in terms of cytocompatibility and cellular functionality.

Fig S6 C, D

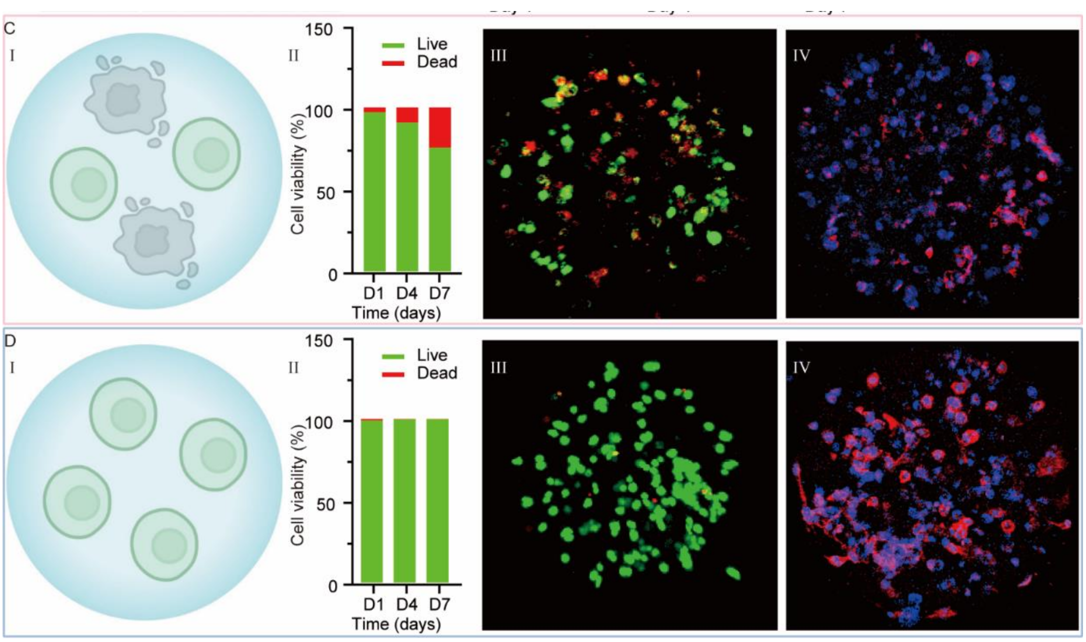

C–D) Comparative biocompatibility analysis of different cell-laden microgels: homogeneous microgels prepared via conventional oil-phase microfluidics (C), and homogeneous microgels prepared by ATPS-based microfluidics (D). For each group: I. Schematic illustration; II. Quantification of live/dead staining at days 1, 4, and 7; III. Confocal images of live/dead staining on day 7; IV. Confocal images of F-actin staining.

Line155-156

We successfully achieved tunable control over the porosity of the microgels (Fig. S3I, J)

Fig S3 I, J

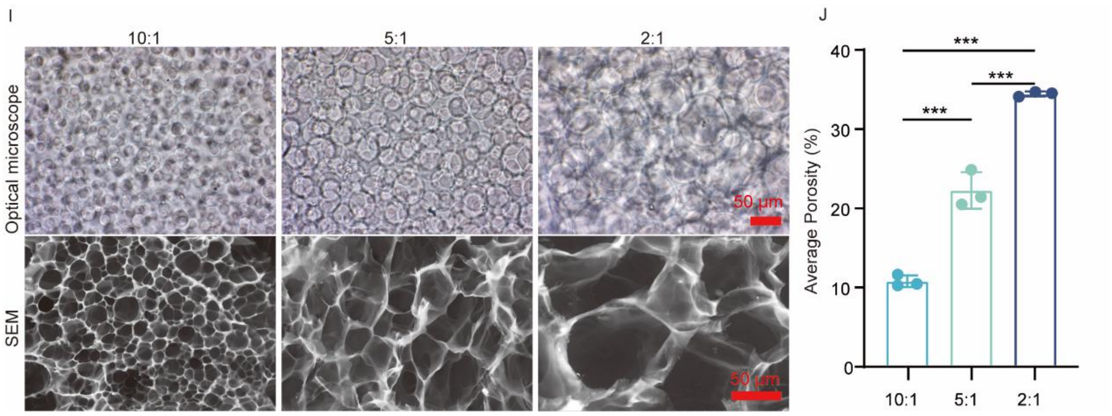

I–J) Bright-field and SEM images (I) of porous GelMA MGs with different porosities obtained by varying the volume ratio of GelMA to PEO, and the corresponding porosity quantification graph (J) (n = 3).

2. The mechanism of interaction between FimH and mannan oligosaccharides has been reported in the previous work. What's the promotion of microgels in this work?

Response:

We thank the reviewer for their valuable comment. Indeed, the interaction between FimH and MOS has been previously reported. However, in our study, the porous microgels act as an advanced multifunctional carrier system, which significantly enhances the therapeutic efficacy and flexibility of MOS-based strategies through the following key advantages:

Protection and targeted delivery: The microgels physically encapsulate MOS, shielding it from harsh gastric conditions and enzymatic degradation. This ensures its bioavailability specifically in the inflamed intestinal region, where AIEC colonization occurs.

Bacterial sequestration and clearance: The microgels demonstrate a high capacity for capturing AIEC and enabling its subsequent elimination via natural intestinal transit. This approach facilitates a direct reduction in the intraluminal bacterial burden, representing a mechanistic advancement over free MOS, which merely inhibits AIEC adhesion to epithelial surfaces without effectively decreasing its abundance within the gut environment.

Preservation of microbial homeostasis: In contrast to free MOS, which may be metabolized as a carbon source and disturb the gut microbiota balance, the microgel-conjugated MOS remains localized and functionally targeted, minimizing off-target effects and maintaining microbiome stability.

Versatile drug-loading platform: Importantly, our microgel system is not limited to MOS. It is a modular platform capable of loading various bioactives, such as anti-inflammatory agents, antimicrobial peptides, or other oligosaccharides, allowing customized treatment strategies tailored for different stages or types of gut disorders.

In summary, the microgels in our work do more than enhance MOS functionality—they represent a broadly applicable therapeutic scaffold with integrated protection, targeting, and clearance mechanisms for effective and safe IBD treatment.

3. In the in vitro co-culture system of microgels and AIEC, what effects does the adsorption of AIEC onto microgels have on their physicochemical properties?

Response:

We thank the reviewer for their valuable comment. In response, we have supplemented additional data analyzing the physicochemical properties of the microgels after co-incubation with AIEC in the in vitro co-culture system (Fig. S5 H). We characterized the morphology and assessed the mechanical properties of selected microgels pre- and post-adsorption, with corresponding images and quantitative data incorporated in the revised manuscript. In addition to the previous conclusion that the microgels showed no significant changes (as shown in Figure 4), The further results indicate that: there was no significant change in the stress-strain curves before and after incubation

Relevant content

Lines 178-180

No significant difference was observed compared to the control group without AIEC (Fig. S5H), suggesting that the adsorption process did not compromise microgel mechanics.

Fig S5 H

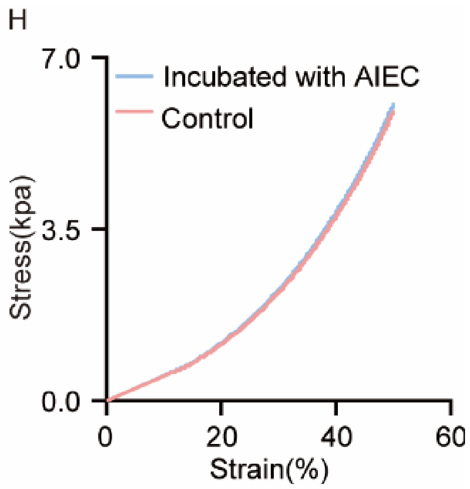

H) Stress–strain curves of MOS MGs before and after 24 h co-incubation with AIEC.

4. In the in vivo experiment, the fate of microgels that have already absorbed AIEC was important but not investigated. After scavenging the AIEC, the alteration of the microgel complex and how it was drawn out were not fully understood.

Response:

We thank the reviewer for their valuable comment. To investigate the fate of microgels after scavenging AIEC in vivo, we treated mice with MOS-GelMA-FITC microgels and collected fecal samples for analysis. The samples were vortexed to form suspensions and examined using fluorescence microscopy. Intact fluorescent microgels were clearly observed in the fecal suspensions, indicating that the microgels maintained their structural integrity and could be excreted intact. (Fig. S5I)

Relevant content

Lines 180-184

To determine whether the AIEC-bound microgels could be excreted from the body, we collected fecal samples from treated mice and successfully isolated the microgels (Fig. S5I). This shows that MOS MGs can maintain their spherical form during intragastric administration, which is an ideal carrier for drug delivery.

Fig S5 I

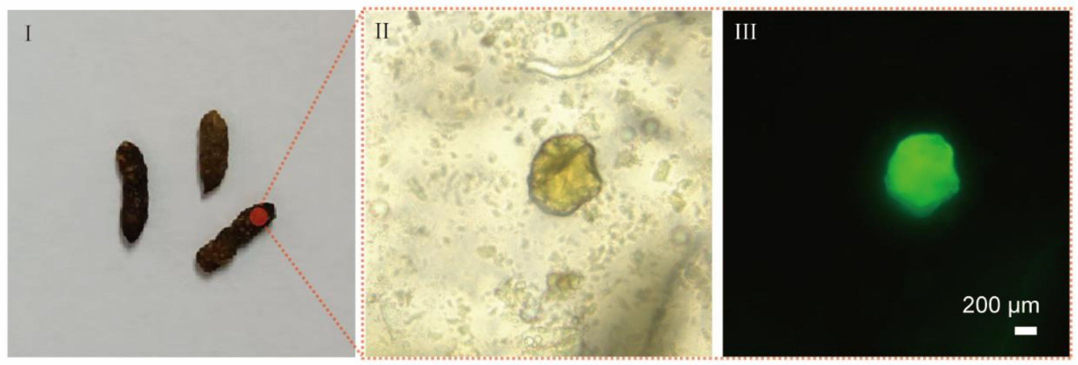

I) Bright-field and fluorescence images of MOS MGs recovered from mouse feces after administration.

5. The microgels contribute to the recovery via the competitive interaction with AIEC when compared with epithelial glycoproteins. Consequently, in vitro experiments probing such competitive interactions, such as co-culturing microgels with AIEC in the presence of colonic epithelial, should be conducted.

Response:

We thank the reviewer for the valuable comment. In the revised manuscript, we have

conducted additional in vitro competitive adsorption experiments. Specifically, we co-incubated Caco-2 cells with AIEC in the presence of MGs and MOS MGs. We then assessed bacterial adhesion by performing plate counting assays and confocal microscopy to evaluate changes in the number of bacteria adhering to the cell surface after microgel addition. The results showed that MOS MGs significantly reduced AIEC adhesion to Caco-2 cells, further supporting their competitive adsorption ability. The relative information has been added in the revised manuscript.

Related content

Lines 260-265

*To evaluate competitive adhesion inhibition, Caco-2 cells were co-incubated with AIEC in the presence of MOS MGs or MGs. Results from both plate counting (**Fig. 4E**) and confocal microscopy (**Fig. 4F**) consistently demonstrated that the number of bacteria adhering to the cell surface was significantly reduced in the MOS MGs group compared to the control group.*

Fig 4 E-F

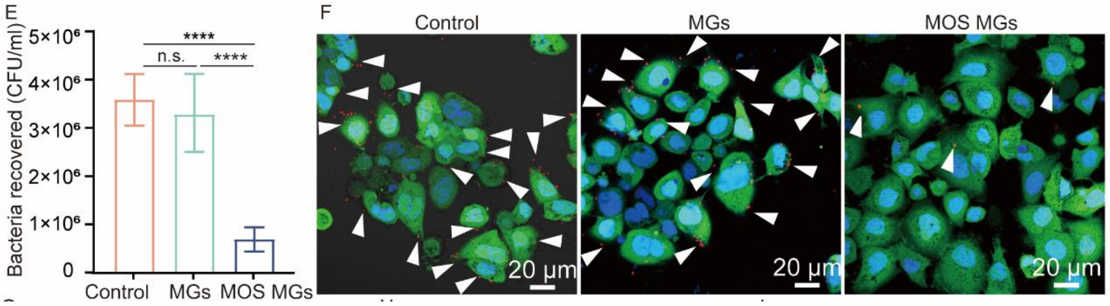

E) Competitive adhesion assay of AIEC between MOS MGs and Caco-2 cells, visualized by colony-forming unit (CFU) plating (n=3). F) Confocal imaging of competitive adhesion of AIEC between MOS MGs and Caco-2 cells. Red: AIEC; blue: DAPI-stained nuclei; green: Calcein-AM-stained cells; white arrows indicate AIEC.

6. In this work, the authors only did the experiment in ulcerative colitis model but IBD includes both ulcerative colitis and Crohn's disease. So, the claim that the strategy for managing IBD is not accurate enough.

Response:

We thank the reviewer for their valuable comment. We agree that IBD includes both

ulcerative colitis (UC) and Crohn’s disease (CD). While the DSS-induced mouse model is widely considered representative of UC, our study used AIEC, a microorganism associated with CD, as the pathogenic strain. Notably, DSS-induced colitis is also commonly employed in AIEC-related studies as an animal model to mimic the compromised intestinal barrier observed in CD. (PMID: 38127978, PMID: 33769191, PMID: 37800577) To avoid overgeneralization, we have carefully revised the manuscript to refer to “DSS-induced colitis” instead of “IBD” where appropriate (Lines 303).

Response to Referees

Reviewer Comments:

Reviewer #1 (Remarks to the Author):

The authors have adequately addressed my previous comments and revised the manuscript accordingly.

Response:

We sincerely thank the reviewer for their positive evaluation. We are grateful for your constructive comments and suggestions throughout the review process, which have significantly improved the quality and clarity of our manuscript.

1. I have only a few minor suggestions:

Figures 4G–I and S8: Please indicate in the figure legend the specific time point post-infection at which the samples were analyzed.

Response:

We appreciate the reviewer's careful comment. The samples in Figures 4G–I and S8 were collected at 48 hours post-infection. We have now revised the figure legends accordingly to clearly indicate this time point.

Figures 4G–I

G–I) Colonization levels of WT in feces (G), colon (H), and ileum (I) at 48 hours post-treatment with MOS, MGs, MOS MGs, and M4284. (n=6).

Supplementary Figure 8

A–C) Colonization levels of ΔfimH in feces (A), colon (B), and ileum (C) at 48 hours post-treatment with MOS, MGs, MOS MGs, and M4284 (n=6).

2. The figure legends state that statistical analyses were performed using a t-test. However, when comparing multiple conditions, a one-way ANOVA should be used. Additionally, for all figures comparing more than two experimental conditions, ANOVA should be used. Please indicate which groups were compared by adding connecting lines to the graphs.

Response:

Thank you for the suggestion. As advised, we have replaced the t-test with one-way ANOVA for Figures 3B, 3E; 4E; 5C–E; 6G–L; S3J; S6B; and S9A–F, M, where multiple groups were compared. The statistical results remain consistent with our

previous findings. We have also updated the figure legends to reflect the use of one-way ANOVA accordingly.

3. I do not have access to the supplemental tables. If not already included, please provide a table listing the sequences of the primers used for RT-qPCR.

Response:

We appreciate your careful review. The sequences of the primers used for RT-qPCR have been included in the supplementary data tables as requested.

Primers for qRT-PCR	
F	ATGGCCTTCCGTGTTCCTAC
R	CAGATGCCTGCTTCACCAC
F	ATGGCAACTGTTCCTGAACTCAACT
R	CAGGACAGGTATAGATTCTTTCCTTT
F	CTAGGTTTGCCGAGTAGATCT
R	CACAAAGCCAGAGTCCTTCAGAGA
F	CAACATACAGTGACGCTTCACA
R	CACTATTGACGTTTCCCCACTC
F	GAGGCCAAGCCCTGGTATG
R	CGGGCCGATTGATCTCAGC

Reviewer #2 (Remarks to the Author):

The authors have carefully considered the reviewers' comments in the revised version of the manuscript, adding new experimental data and references to support their conclusions and improving the methods and discussion sections. Therefore, I recommend publishing the manuscript in Nature Communications.

Response:

We sincerely thank the reviewer for the positive feedback and thoughtful recommendation. We truly appreciate your valuable comments and support throughout the review process, which have helped us improve the quality and clarity of our manuscript.

Reviewer #3 (Remarks to the Author):

I have reviewed the authors' responses and the revisions made to the manuscript. I have no further questions with the technical and experimental aspects of the paper. However, I remain unconvinced about the novelty of the work. The fabrication method and materials used to prepare the porous microgels lack novelty. Crucially, the key design of competitively selective bacterial capture has been previously reported. The only significant contribution appears to be the in vivo therapeutic study, which is insufficient to warrant publication in a high-impact journal of Nature Communications.

Response:

We sincerely thank the reviewer for the time and effort dedicated to evaluating our manuscript. We respect your opinion regarding the assessment of novelty. However, we would like to clarify that while some aspects of the fabrication method and materials are based on established techniques, our work introduces a strategically integrated system combining aqueous two-phase microfluidics to enable scalable production of monodisperse microgels without requiring surfactants or oils and competitive bacterial capture with targeted in vivo delivery—specifically tailored for AIEC clearance in IBD treatment.We believe that this integration of bacterial adhesion biology, biomaterial engineering, and disease-specific animal models represents an interdisciplinary advancement, moving beyond isolated technique development. That said, we appreciate

your perspective and will continue to refine our approach and presentation in future work.
